# Supplementary material for: Active case-finding for TB in India: Assessment of scale and quality benchmarks, time taken and use of rapid molecular diagnostic tests
Source: PLOS Glob Public Health. 2025 Oct 30;5(10):e0005103. doi: 10.1371/journal.pgph.0005103 (PMC12574901; doi:10.1371/journal.pgph.0005103)
Supplement: S1 File — (PDF) [file pgph.0005103.s001.pdf]

ACF tool to be used in the field for individual level data collection of those screened - Daily ACF field activity form

**State:**	**District Name:**	**TU Name:**	**PHI Name:**
**Exact address of mapped area:**	**District Code:**		
**Date of ACF activity(dd/mm/yyyy):**	**Type of marginalized / vulnerable pop**	**TU Code:**	

						**Symptoms (in days)**														
**S.no**	**Name**	**Mobile Number**	**Age in year**	**Sex (M/F/O)**	**Address**	**Cough**	**Fever**	**Weight loss**	**Haemoptysis**	**Chest pain**	**X-ray taken for screening? (Yes / No)**	**If yes, any abnormality present in X-ray? (yes / No)**	**Presumptive TB? (Yes/No)**	**Date of sputum collection (dd/mm/yy)**	**Date of sputum examination (dd/mm/yy)**	**Whether rapid diagnostic test used upfront? (Yes/No)**	**TB diagnosed yes / No**	**Date of treatment start (dd/mm/yy)**	**Date of notification (dd/mm/yy)**	**NIKSHAY ID IF TB DIAGNOSED**
1																				
2																				
3																				
4																				
5																				
6																				
7																				
8																				
9																				
10																				
11																				
12																				
13																				
14																				
15																				

						Symptoms														
**S.no**	**Name**	**Mobile Number**	**Age in year**	**Sex (M/F/O)**	**Address**	**Cough**	**Fever**	**Weight loss**	**Haemoptysis**	**Chest pain**	**X-ray taken for screening ? (Yes / No)**	**If yes, any abnormality present in X-ray ? (yes / No)**	**Presumptive TB? (Yes/No)**	**Date of sputum collection (dd/mm/yy)**	**Date of sputum examination (dd/mm/yy)**	**Whether rapid diagnostic test used upfront ? (Yes/No)**	**TB diagnosed yes / No**	**Date of treatment start (dd/mm/yy)**	**Date of notification (dd/mm/yy)**	**NIKSHAY ID IF TB DIAGNOSED**
16																				
17																				
18																				
19																				
20																				
21																				
22																				
23																				
24																				
25																				
26																				
27																				
28																				
29																				
30																				
