# Supplementary material for: Active case-finding for TB in India: Assessment of scale and quality benchmarks, time taken and use of rapid molecular diagnostic tests
Source: PLOS Glob Public Health. 2025 Oct 30;5(10):e0005103. doi: 10.1371/journal.pgph.0005103 (PMC12574901; doi:10.1371/journal.pgph.0005103)
Supplement: S1 Fig — * Map created using QGIS software using publicly available and free of cost shape file of districts of India (basefile) from Survey of India website (https://onlinemaps.surveyofindia.gov.in/). (DOCX) [file pgph.0005103.s002.docx]

**S1 Fig.** Thirty randomly sampled NTEP districts^a^ from nine states of India, phase three of central TB division commissioned national level TB ACF evaluation project, India.


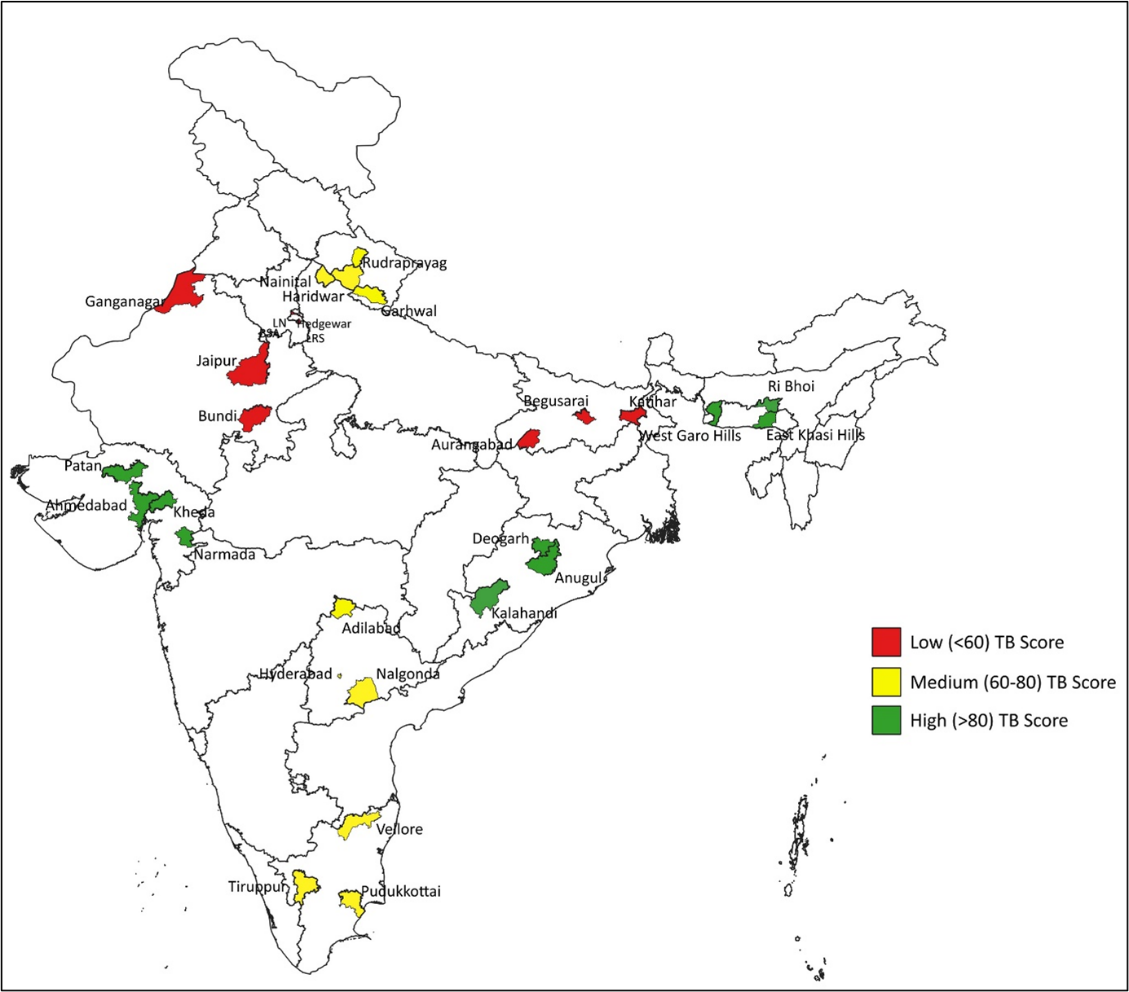


Abbreviations: ACF, active case-finding; TB, tuberculosis; NTEP, national TB elimination program

^a^States were stratified based on the routinely available composite TB score measuring NTEP performance and TB burden for every state (>80 classified as high, 60 to 80 as medium and <60 as low performance). Three states were selected from each stratum by simple random sampling. From the nine states, 30 NTEP districts were selected based on probability proportionate to size sampling (based on TB notification)
