# Supplementary material for: Active case-finding for TB in India: Assessment of scale and quality benchmarks, time taken and use of rapid molecular diagnostic tests
Source: PLOS Glob Public Health. 2025 Oct 30;5(10):e0005103. doi: 10.1371/journal.pgph.0005103 (PMC12574901; doi:10.1371/journal.pgph.0005103)
Supplement: S1 Table — (DOCX) [file pgph.0005103.s003.docx]

**S1 Table.** The three revised^a^ ACF scale and quality indicators at national level (2021) and their cut-offs used in the first phase of TB ACF evaluation project [17]

| **Revised TB ACF indicators** | **Targets for first phase (rationale)** [17] | **Observed (national)** |
| --- | --- | --- |
| Percentage screened among the population^b^ | **at least 10%** (at least 11% of the district population to be mapped as marginalized/vulnerable * at least 90% of the mapped population should be screened = 10%) | 9.3% |
| Percentage tested among screened^c^ | **at least 4.75%** (at least 5% of screened to be identified as presumptive TB * at least 95% of presumptive should be tested = 4.75%) | 1% |
| Percentage diagnosed (microbiologically / clinically) among tested^c^ | at least 5 % (no change) | 3.7% |

Abbreviations: TB, tuberculosis; ACF, active case-finding

^a^data on high-risk populations and presumptive TB detected were not consistently available across districts to calculate all the ACF quality indicators as recommended by the program and shown in Fig 1 [17]

^b^ACF scale

^c^ACF quality indicator
