# Supplementary material for: Active case-finding for TB in India: Assessment of scale and quality benchmarks, time taken and use of rapid molecular diagnostic tests
Source: PLOS Glob Public Health. 2025 Oct 30;5(10):e0005103. doi: 10.1371/journal.pgph.0005103 (PMC12574901; doi:10.1371/journal.pgph.0005103)
Supplement: S2 Table — (DOCX) [file pgph.0005103.s004.docx]

**S2 Table.** Time taken in the care cascade in the TB ACF cycle conducted from January to September 2023 in 30 randomly sampled NTEP districts of India

| **Time taken (days)** | **Number eligible**^a^ | **Number both dates available** | **Median** | **(IQR)** |
| --- | --- | --- | --- | --- |
| Among people with presumptive TB whose sputum specimen was collected, from ACF activity to sputum specimen collection | 8541 | 8471 | 0 | (0,1) |
| Among people with presumptive TB whose sputum specimen was tested, from sputum specimen collection to testing | 7527 | 7456 | 1 | (0,2) |
| Among people with presumptive TB whose sputum specimen was tested, from ACF activity to testing | 7527 | 7520 | 1 | (0,2) |

Abbreviations: TB, tuberculosis; ACF, active case-finding; NTEP, national TB elimination program; IQR, interquartile range

^a^of 581 633 screened, 11 357 were identified as presumptive TB
