# Supplementary material for: Active case-finding for TB in India: Assessment of scale and quality benchmarks, time taken and use of rapid molecular diagnostic tests
Source: PLOS Glob Public Health. 2025 Oct 30;5(10):e0005103. doi: 10.1371/journal.pgph.0005103 (PMC12574901; doi:10.1371/journal.pgph.0005103)
Supplement: Attachment — Submitted filename: PGPH-D-25-01505_R1_Comments PK.pdf [file pgph.0005103.s008.pdf]

PLOS Global Public Health

Active case-finding for TB in India: Assessment of scale and quality benchmarks, time taken and use of rapid molecular diagnostic tests

--Manuscript Draft--

**Manuscript Number:**	PGPH-D-25-01505R1
**Article Type:**	Research Article
**Full Title:**	Active case-finding for TB in India: Assessment of scale and quality benchmarks, time taken and use of rapid molecular diagnostic tests
**Short Title:**	ACF for TB in India (2023)
**Corresponding Author:**	Hemant Deepak Shewade, MBBS MD PhD<br>National Institute of Epidemiology<br>Chennai, INDIA
**Order of Authors:**	Hemant Deepak Shewade, MBBS MD PhD
	S Kiran Pradeep, MBBS MD
	Prabhadevi Ravichandran
	G Kiruthika
	Amar N Shah
	Bhavin Vadera
	R Sabarinathan
	Venkatesh Roddawar
	Sanjay K Mattoo
	Swati Iyer
	Dheeraj Tumu
	Aniket Chowdhury
	Shanmugasundaram Devika
	Joshua Chadwick
	Rakesh R Vaidya
	Prasanta Kumar Hota
	Asha Frederick
	Pankaj Singh
	Bayarilin Shanpru
	Gunjan Khunger
	Subrata Kumar Panda
	Mohamed Arif Baig
	K V Suma
	Mahendran Suleka
	Amit Kumar Digal
	Debobrata Banerjee
	Machupalli Lakshmi Prasanna
	Divya Yashpal Waghela
	A Krishnaraj

Powered by Editorial Manager® and ProduXion Manager® from Aries Systems Corporation

	Pratibha Kashyap
	Jayeshbhai Sendhabhai Parmar
	Sushmita Das
	Ajit Kumar
	Ajay Kumar
	Sunny Yadav
	Shradha Chetri
	Subhashree Suchismita Mahapatra
	Amit Kumar
	Monu Pathak
	Sachin Singh
	Shams Tabrez
	Piyush Mehra
	Bipra Bishnu
	G Mahesh
	Biswabihari Mohanty
	A Rajesham
	Bal Krishna Mishra
	Dinesh N Barot
	Umesh Chandra Tripathi
	Khalid Umer Khayyam
	Kiran Rade
	Raghuram Rao
	Manoj V Murhekar

Keywords: India; Operational research; TB ACF cycle; TB ACF quality indicators; high-risk groups; number needed to screen

Abstract: Since 2017, tuberculosis active case-finding (TB ACF) in India is being implemented in routine national TB elimination program settings. Symptom screen of high-risk population followed by confirmation of TB among symptom-screen positive is the TB ACF algorithm. TB ACF scale and quality assessments hitherto were predominantly local or based on aggregate program data with limited details on all the indicators, the extent of use of rapid molecular diagnostic tests and the time taken. As a part of the national TB elimination program commissioned evaluation, we assessed one ACF cycle (intention to screen the high-risk population once) between January and September 2023 using nationally representative prospectively collected individual level data. In this cohort study from high-risk populations in 30 randomly sampled districts (nine states), 581 633 high-risk individuals were screened by the program utilizing existing workforce and resources. The two scale indicators (target, observed) were: percentage of i) population mapped as high-risk (≥11%, 18.3%) and ii) mapped population screened (≥90%, 7.4%). The four quality indicators (target, observed) were: percentage of i) screened identified as presumptive TB (≥5%, 2%) ii) presumptive TB tested (≥95%, 66.3%) iii) tested diagnosed as TB (≥5%, 1.6%) and iv) diagnosed put on treatment (≥95%, 100%). The number needed to screen (target) to detect one person with TB was 4971 (≤1538 considering the algorithm). This was observed across most of the high-risk groups, with few exceptions. The extent of using rapid molecular diagnostic tests was 26.4% and the median time taken from screening to sputum collection and testing was one day. To conclude, ACF scale and quality were grossly below the benchmark, lower than previously reported (2021) based on retrospective

Powered by Editorial Manager® and ProduXion Manager® from Aries Systems Corporation

	aggregate program data. Effective planning, resource allocation including use of rapid molecular diagnostic tests and individual data recording among those screened to facilitate implementation monitoring are recommended.
**Additional Information:**	
**Question**	**Response**
**Financial Disclosure**<br><br>Enter a financial disclosure statement that describes the sources of funding for the work included in this submission and the role the funder(s) played. This includes grants and any commercial funding of the work or authors.<br><br>This statement will be typeset if the manuscript is accepted for publication.<br><br>*Please review the submission guidelines and the instructions link below for detailed requirements and guidance.*	This study was funded by USAID through John Snow International under the TB Implementation Framework Agreement (TB commitment grants 0011-0549-1024 and 0011-0549-1025 to MVM) . All authors had full access to all the data and had final responsibility for the decision to submit for publication.
**Competing Interests**<br><br>On behalf of all authors, disclose any competing interests that could be perceived to bias this work.<br><br>This statement will be typeset if the manuscript is accepted for publication.<br><br>*Please review the instructions link below and PLOS Global Public Health's competing interests policy to determine what information must be disclosed at submission.*	There are no undeclared conflicts of interest
**Data Availability**<br><br>Before publication, Authors are required to make fully available and without restriction all data underlying their findings. Please see our PLOS Data Policy page for detailed information on this policy.	All the data are fully available without restriction<br>Figshare: The link contains a collection of the dataset (EpiData REC file), the codebook/data dictionary for dataset (MS Excel) and the programme file for analysis of dataset (EpiData PGM file) pertaining to this study.<br>https://doi.org/10.6084/m9.figshare.c.7048385.v1<br>Data are available under the terms of the Creative Commons Attribution 4.0 International License (CC BY 4.0)

A **Data Availability Statement**, detailing where the data can be accessed, is required at first submission. Insert your Data Availability Statement in the box below.<br><br>Please see the data reporting section of our submission guidelines for instructions on what you need to include in your Data Availability Statement.<br><br>This statement will be typeset if the manuscript is accepted for publication.<br><br>*PLOS allows rare exemptions to address legal and ethical concerns. If you have legal or ethical restrictions, please use the box below to detail these in full sentences for the Journal team to consider.*	

Powered by Editorial Manager® and ProduXion Manager® from Aries Systems Corporation

Revised Manuscript Clean
Click here to access/download;Manuscript;Manuscript.docx

ORIGINAL ARTICLE

Active case-finding for TB in India: Assessment of scale and quality benchmarks, time taken and use of rapid molecular diagnostic tests

Hemant Deepak Shewade[a,†,*], S Kiran Pradeep[a,†], Prabhadevi Ravichandran[a], G Kiruthika[a], Amar N Shah[b], Bhavin Vadera[b], R Sabarinathan[a], Venkatesh Roddawar[c], Sanjay K Mattoo[d], Swati Iyer[e], Dheeraj Tumu[e], Aniket Chowdhury[e], Shanmugasundaram Devika[a], Joshua Chadwick[a], Rakesh R Vaidya[f], Prasanta Kumar Hota[g], Asha Frederick[h], Pankaj Singh[i], Bayarilin Shanpru[j], Gunjan Khunger[k], Subrata Kumar Panda[e], Mohamed Arif Baig[e], K V Suma[e], Mahendran Suleka[a], Amit Kumar Digal[a], Debobrata Banerjee[a], Machupalli Lakshmi Prasanna[a], Divya Yashpal Waghela[a], A Krishnaraj[a], Pratibha Kashyap[a], Jayeshbhai Sendhabhai Parmar[a], Sushmita Das[a], Ajit Kumar[a], Ajay Kumar[a], Sunny Yadav[a], Shradha Chetri[a], Subhashree Suchismita Mahapatra[a], Amit Kumar[a], Monu Pathak[a], Sachin Singh[a], Shams Tabrez[a], Piyush Mehra[a], Bipra Bishnu[e], G Mahesh[e], Biswabihari Mohanty[l], A Rajesham[m], Bal Krishna Mishra[n], Dinesh N Barot[o], Umesh Chandra Tripathi[e], Khalid Umer Khayyam[p], Kiran Rade[e,@], Raghuram Rao[d,@], Manoj V Murhekar[a,@]

[a]ICMR-National Institute of Epidemiology (ICMR-NIE), Chennai, India

[b]USAID India, New Delhi, India

[c]John Snow India Private Limited, New Delhi, India

[d]Central TB Division, Ministry of Health and Family Welfare, New Delhi, India

[e]Office of the World Health Organization (WHO) Representative to India, WHO Country Office, New Delhi, India

[f]State TB Cell, Government of Gujarat, Ahmedabad, India

[g]State TB Cell, Government of Odisha, Bhubaneshwar, India
[h]State TB Cell, Government of Tamil Nadu, Chennai, India
[i]State TB Cell, Government of Uttarakhand, Dehradun, India
[j]East Khasi Hills District TB Cell, Government of Meghalaya, Shillong India
[k]Ganganagar District TB Cell, Government of Rajasthan, Ganganagar, India
[l]Anugul District TB Cell, Government of Odisha, Anugul ,India
[m]State TB Cell, Government of Telangana, Hyderabad, India
[n]State TB Cell, Government of Bihar, Patna, India
[o]Kheda District TB Cell, Government of Gujarat, Nadiad, India
[p]National Institute of Tuberculosis and Respiratory Diseases, Government of India, New Delhi, India
[†]joint primary authors; [@]joint senior authors

**Correspondence to:* Dr Hemant Deepak Shewade, MBBS MD PhD, Scientist E, Division of Health Systems Research, ICMR-National Institute of Epidemiology (ICMR-NIE), Chennai 600077, India. E-mail: hemantjipmer@gmail.com)

Short title: ACF for TB in India (2023)

Key words: India; Operational research; TB ACF cycle; TB ACF quality indicators; high-risk groups; number needed to screen.

Manuscript details

Word count: Abstract (299 words); main text (4273 words); 2 figures; 3 tables; 1 supplementary figure; 2 supplementary tables; 1 supplementary annex; 36 references

ABSTRACT

Since 2017, tuberculosis active case-finding (TB ACF) in India is being implemented in routine national TB elimination program settings. Symptom screen of high-risk population followed by confirmation of TB among symptom-screen positive is the TB ACF algorithm. TB ACF scale and quality assessments hitherto were predominantly local or based on aggregate program data with limited details on all the indicators, the extent of use of rapid molecular diagnostic tests and the time taken. As a part of the national TB elimination program commissioned evaluation, we assessed one ACF cycle (intention to screen the high-risk population once) between January and September 2023 using nationally representative prospectively collected individual level data. In this cohort study from high-risk populations in 30 randomly sampled districts (nine states), 581 633 high-risk individuals were screened by the program utilizing existing workforce and resources. The two scale indicators (target, observed) were: percentage of i) population mapped as high-risk (≥11%, 18.3%) and ii) mapped population screened (≥90%, 7.4%). The four quality indicators (target, observed) were: percentage of i) screened identified as presumptive TB (≥5%, 2%) ii) presumptive TB tested (≥95%, 66.3%) iii) tested diagnosed as TB (≥5%, 1.6%) and iv) diagnosed put on treatment (≥95%, 100%). The number needed to screen (target) to detect one person with TB was 4971 (≤1538 considering the algorithm). This was observed across most of the high-risk groups, with few exceptions. The extent of using rapid molecular diagnostic tests was 26.4% and the median time taken from screening to sputum collection and testing was one day. To conclude, ACF scale and quality were grossly below the benchmark, lower than previously reported (2021) based on retrospective aggregate program data. Effective planning, resource allocation including use of rapid molecular diagnostic tests and individual data recording among those screened to facilitate implementation monitoring are recommended.

AUTHOR SUMMARY

In India, tuberculosis active case-finding scale and quality assessments hitherto were predominantly local or based on aggregate program data with limited details on all the indicators and the extent of use of rapid molecular diagnostic tests for confirmation of tuberculosis. We used nationally representative prospectively collected individual level data. In 2023, scale and quality of active case-finding for tuberculosis was below the benchmark, lower than previously reported (2021) based on retrospective aggregate program data. Among active case-finding-detected presumptive tuberculosis, rapid molecular diagnostic tests were used in one-fourth (similar extent of use in passive case-finding). We recommend effective planning and resources (including access to rapid molecular diagnostic tests) to improve scale and quality. Individual data recording among those screened should be routinely done to facilitate implementation monitoring.

INTRODUCTION

Globally, we have made significant progress in reducing the missing people with tuberculosis (TB) [1,2]. To detect people with TB, national TB programs have relied heavily on passive case-finding (PCF) [3–5]. To detect the missing people with TB (3.1 million in 2022) and put them on treatment, other case-finding strategies are employed [2,6]. Active case-finding (ACF) is systematic screening for TB disease implemented outside of health facilities. This includes household contact investigation and community-based systematic screening of high-risk populations [3]. World Health Organization recommends conducting ACF in high-risk populations that have at least 0.5% prevalence of undetected TB and not in general population [6].

Many systematic reviews have synthesized information regarding effectiveness of ACF in high-risk populations overall and also in migrants, household contacts and prisons [7–10]. Even in low TB incidence countries, targeting high-risk populations has been found to be a cost-effective strategy [11]. The effectiveness of ACF on TB transmission depends on the frequency of ACF cycles per year (number of times high-risk population are screened in a year) and yield of an ACF cycle (extent of estimated undetected TB in the high-risk population that is detected because of ACF). Yield of an ACF cycle depends on the choice of high-risk populations, the scale (proportion of high-risk population in a geographic area screened) and quality (NNS - number needed to screen) of an ACF cycle, and use of sensitive screening algorithms [12,13]. Most studies assessing ACF care cascade focus on quality (NNS) and number needed to test (test-positivity). There are limited studies that describe frequency, scale and yield.

Globally, India has the highest TB burden with 0.5 million missing TB in 2022 [2,14]. A systematic review (2022) revealed that ACF implementation is highly variable in India and

current literature is sparse for many important risk groups [15]. Additionally, studies focussing on ACF care cascade from India have three limitations; either they are local with limited scope for extrapolating the findings nationally [16–23], or are based in project settings [22–25], or based on aggregate retrospective secondary data [26,27]. We have limited data on all the scale and quality indicators, the time taken, and the extent of use of rapid molecular diagnostic tests in the care cascade [16–27].

In 2022, India's National TB Elimination Program (NTEP) commissioned a national level TB ACF evaluation project (August 2022 to February 2024) to guide evidence-based strategic planning. Under the first phase of this project, we assessed the frequency, scale and quality of ACF at district, state and national level for the year 2021 using retrospective secondary aggregate ACF data from *Ni-kshay* (a case-based, web-based electronic TB information management system under NTEP) [26]. Data on high-risk populations, ACF-detected presumptive TB and ACF-detected TB initiated on treatment were not available to calculate all the scale and quality indicators (see Figure 1 for all program-recommended indicators). Hence, we used three revised indicators based on the available data (see Supplementary Table 1) [26]. The findings revealed that most of the states implemented one ACF cycle, with suboptimal scale and quality (Supplementary Table 1) [26]. Though ≈9% (revised target 10% for scale) of district population was reported to be screened, we were not sure whether these were high-risk populations [26].

A nationally representative prospective study involving individual-level data from ACF cycle with reliable estimates of all scale and quality indicators (see Figure 1) was required. Recording individual data of those screened allows random checks of the aggregate numbers reported in ACF care cascade (facilitates implementation monitoring). This would add to our existing knowledge and suggest modifications required in the national ACF guidance [28,29].

Hence, under the third phase of the NTEP commissioned national level TB ACF evaluation project, we estimated at national level (2023), i) all ACF scale and quality indicators, overall and stratified by key high-risk populations ii) the time taken in the care cascade and iii) the extent of use of rapid molecular diagnostic tests among ACF-detected people with presumptive TB.

METHODS

Study design

This was a cohort study using individual level secondary data.

Study setting

India has 36 state-level TB cells and 768 NTEP districts. At the national level, the central TB division provides guidance and leadership to state and district TB cells [14]. A district programme coordinator supports the district TB officer. In public sector, the lowest level of peripheral health institution (PHI) facilitating TB diagnosis is the primary health centre that has at least one medical doctor and laboratory technician for sputum examination. Paper-based registers maintained at PHIs and sub-district level administrative units (TB units) have a line-list of people with TB notified, their management, and the treatment outcomes. Each TB unit has a senior TB treatment supervisor who transcribes these details in *Ni-kshay* [30]. People with TB receive daily ambulatory directly observed treatment by a health care provider, community volunteer or a family member.

Since 2017, India is implementing ACF in high-risk populations of all the districts [29]. Budgets (including incentives) are accounted for in the program implementation plans of the districts. All high-risk populations are to be identified (mapped) at the start of the year and added to *Ni-kshay* [29]. High risk populations are broadly classified into clinically, socio-economically and geographically high risk (see Table 1). Against each mapped population, aggregate numbers linked to each ACF activity day are captured: date of ACF activity, number screened, number tested and number diagnosed (number of ACF-detected presumptive TB is not captured). ACF activity days are the days identified by the district in the year when ACF will be done, irrespective of the mode (campaign mode, in multiple rounds or on fixed days of a month) or scale.

The algorithm used for ACF under NTEP involves symptom-screen (among high-risk populations) followed by sputum microscopy or rapid molecular test (Xpert MTB/Rif or TrueNat) among symptom-screen positive, also called as ACF-detected presumptive (pulmonary) TB. Symptom-screen positive is defined as the presence of cough or fever of two or more weeks or unexplained weight loss or chest pain or hemoptysis. Among clinically vulnerable, cough or fever of any duration is considered [28,29].

The national ACF guidance provides benchmarks for ACF scale and quality (Figure 1) but not for time taken and extent of use of rapid molecular diagnostic tests in the care cascade [28,29]. The 2017 ACF guidance recommended the use of a paper-based ACF tool in the field to collect individual level information of those screened, based on which aggregate numbers are to be filled in *Ni-kshay* [29]. The ACF tool includes population level information regarding the address and the specific type of high-risk population and individual level information like name, age, gender, mobile number, symptom details, presumptive pulmonary TB (yes/no) and details of testing and TB diagnosis if found to be presumptive

TB. The 2019 ACF guidance did not mention the use of this paper-based ACF tool in the field [28]. Most districts and states are implementing one ACF cycle per year with limited use of the paper-based ACF tool in the field [26,31].

Study population

In 30 randomly sampled NTEP districts (nine states) across India (see Supplementary Figure), we included all persons belonging to high-risk populations (not general population) who were screened between January and September 2023 and whose information were collected in a paper-based ACF tool used in the field (mobile-based in three districts belonging to one state). Aggregate data directly entered in *Ni-kshay* without the use of ACF tool was not verifiable and therefore, not considered.

We selected nine states by stratified random sampling based on the routinely available composite TB score measuring NTEP performance and TB burden for every state (>80 score classified as high, 60 to 80 as medium and <60 as low performance). Three states were selected from each stratum by simple random sampling. From the nine states, 30 NTEP districts were selected based on probability proportionate to size sampling (based on TB notification). Three NTEP districts each were selected from six states (Tamil Nadu, Telangana, Rajasthan, Bihar, Odisha, Meghalaya), except Gujarat, Uttarakhand and Delhi, from which four districts were selected (see Supplementary Figure).

Variables, source of data and data collection

During the national/ state/ district level orientation and re-orientation meetings and in writing, it was clarified by the investigator team that individual level data available from the ACF tool used in field (see Supplementary Annex) will be considered as ACF data. The ACF tool used was similar to the one recommended in the 2017 ACF guidance [29], with the addition of two questions: whether chest radiograph was used for screening (yes/no) and whether rapid molecular test used for TB diagnosis (yes/no). Use of the ACF tool provided an opportunity for cross-checking whether ACF happened in mapped high-risk populations, verify a certain proportion of those in the care cascade and provide quality control-linked incentives [31]. This provided a certain sense of reliability to the aggregate numbers generated for the ACF care cascade.

ACF was implemented in routine settings by the health system. The district NTEP team provided overall guidance in planning, training, implementation (including mapping), and monitoring. One project research assistant per district facilitated the use of ACF tool. Within a district at the beginning of the year, in each sub-district level administrative unit (block), high risk populations were mapped (population ID, address, nearest landmark, high-risk population type and high-risk population numbers). Then this information was aggregated at district level to generate total high-risk population: broad and specific high-risk populations type (Table 1). For each ACF activity day, the project research assistant captured information on whether ACF tool was used in the field and compiled the ACF tool at district level. If there was any missing field or data inconsistency, they got it corrected. Beyond this, they did not interfere with the implementation and data quality control mechanism put in place by the district NTEP team.

Data management and analysis

The project research assistants single-entered (with adequate data entry checks) the paper-based ACF tool data (except name and contact number) into a web application with locally stored server at ICMR-National Institute of Epidemiology (ICMR-NIE), Chennai (except three districts belonging to one state that directly used mobile-based version of the ACF tool in the field). Data collection and entry were monitored centrally by the project management team from ICMR-NIE. Descriptive and analytical statistics were used as appropriate using EpiData Analysis software v 2.2.2.183 (EpiData Association, Odense, Denmark).

As we captured ACF data from January to September 2023 and the districts aimed to implement one ACF cycle in 2023, 75% of the mapped high-risk population (aggregate number) was considered as intended for screening (denominator) during the study period.

The two ACF scale indicators (target) were: i) percentage of population mapped as high-risk (≥11%) and ii) percentage of mapped population screened in the ACF cycle (≥90%). The four ACF quality indicators (target) were: i) percentage of screened identified as presumptive TB (≥5%) ii) percentage of presumptive TB tested (≥95%) iii) percentage of tested diagnosed as TB (test positivity ≥5%) and iv) percentage of diagnosed put on treatment (≥95%). These were (see Figure 1) in line with national ACF guidance [28]. NNS (number screened divided by number diagnosed) cut-off (≤1538) for the algorithm summarized ACF quality indicators into one [26]. We calculated the six indicators and NNS, overall and stratified by high-risk populations. It was not possible to provide the two ACF scale indicator estimates by broad high-risk population types. The reason being there were many instances where a mapped population could be classified into multiple specific high-risk population types and these specific high-risk population types could be from more than one broad high-risk population types (see Table 1).

A meaningful difference of at least 10% for proportion of presumptive tested and extent of use of rapid molecular diagnostic test, and at least 2% for other proportions was considered significant if it also achieved statistical significance ($p<0.05$ using chi squared test).

Ethics

This study was part of the third phase of India central TB division commissioned national level TB ACF evaluation project. The evaluation project was approved by ICMR-NIE's ethics committee (NIE/IHEC/202201-10 dated 09 Feb 2022, renewal on 25 Jan 2023). As this study involved routinely collected secondary data (anonymous), waiver of written informed consent was sought and approved by the ethics committee.

RESULTS

The total population of 30 districts was 57 211 032. A total of 10 449 555 (18.3%) were mapped as high-risk, 75% of which (n= 7 837 170) were considered as denominator for further analysis.

There were a total of 1467 ACF activity days. ACF tool was used in the field in 1031 (70.3%) days. During the 1031 ACF activity days considered in the analysis, of the 7 837 170 high-risk population, 581 633 (7.4%, 95% CI: 7.42. 7.44) were screened (an average of 565 people screened per district per ACF activity day). Of these, 11 357 (1.95%, 95% CI: 1.92, 1.99) were identified as presumptive TB. Among those with presumptive TB, the prevalence estimates of symptoms [duration of symptoms] were as follows: cough in 70.3% [median 9 days (interquartile range: 1,15)], fever in 28.7% [median 5 days (interquartile range: 2,7)],

weight loss in 8%, haemoptysis in 2.7% and chest pain in 9.2%. Of 11 357, sputum specimen was collected for 8541 (75.2%) and 7527 (66.3%, 95% CI: 65.4, 67.2) were tested, of which 117 (1.6%, 95% CI: 1.3, 1.9) were diagnosed with TB. All the ACF-detected TB were initiated on treatment. The NNS was 4971. The ACF scale and quality indicators are depicted in Figure 2 and Table 2.

Half of those screened were male, 11% were elderly and 60% resided in rural areas (see Table 2). The most common type of high-risk population (by broad categories) screened was geographically high-risk (41%). Ten percent of high-risk individuals were screened at facilities (see Table 2). Higher percentage of presumptive TB among screened was observed in elderly (5.2% versus 1.6% in ≤60 years) and clinically high-risk (4.1% versus 1.3% in geographically high-risk). The percentage of testing among presumptive TB was low across all sub-groups and ranged from 64% to 71%. Higher test positivity was observed in socioeconomically high-risk (2.9% versus 0.6% in geographically high-risk). NNS was >1538 for all subgroups. It was as high as 18 331 for geographically high-risk (see Table 2).

The distribution of specific high-risk populations that were screened is as follows (those with 2.5% or higher contribution have been mentioned): areas with high notifications (34.1%), slums (20.6%), occupational risk (9.5%), unorganized labour (8.6 %), difficult to reach areas (6.9%), tribal areas (2.9%) and noncommunicable disease and/or its risk factors and/or COVID19 recovered (2.7%). The percentage screened among the mapped population was the highest for unorganized labour (75.3%) with an NNS of 2495. The NNS was below or around the cut-off in prisons (NNS 882), contact tracing (NNS 1583) and noncommunicable disease and/or its risk factors and/or COVID19 recovered (NNS 1288) (Figure 2).

Of 7527 presumptive tested, 1989 (26.4%, 95% CI: 25.4, 27.4) underwent rapid molecular diagnostic test. High-risk populations in rural areas (18.3% versus 42.9% in urban),

population-based high-risk groups (22.7% versus 43.2% for facility-based), and geographically high-risk (5.4% versus 41.1% for socioeconomically) had a significantly lower use (Table 3). The median time taken in the various steps of the care cascade ranged from zero to one day (Supplementary Table 2).

Of the high-risk population screened, a total of 9321 (1.6%) underwent upfront chest radiograph as a screening test and most of these people (n=8854, 95%) were from two districts of a state. Of these 9321, a total of 1087 (11.7%) had an abnormality in chest radiograph, of whom 679 (62.5%) did not have any symptoms.

DISCUSSION

This study on TB ACF care cascade (2023) from India is possibly the first ever nationally representative study using individual level data that assessed the scale and quality of an ACF cycle. During the ACF cycle, less than 10% of high-risk population was screened along with NNS of ≈5000. Time taken in the care cascade was minimal and use of rapid molecular diagnostic tests was observed in less than one-third of those tested. But for the percentage of population mapped as high-risk and percentage ACF-detected TB initiated on treatment, none of the other four scale and quality indicators were above the benchmark. The percentage of mapped high-risk population screened fared the worst and percentage of presumptive TB tested fared the best.

Strengths and limitations

A systematic review assessing the effect of ACF on TB epidemiology recommended that ACF projects should incorporate a well-designed, robust evaluation to contribute to the evidence base [9]. This is one such example. Similar to our 2021 analysis [26], we analysed the ACF scale and quality indicators for an ACF cycle starting from high-risk populations; we did not merely start our analysis from those screened. This provided us with a holistic understanding of low yield, so that corrective actions can be taken. We mapped high-risk populations and prospectively documented ACF care cascade, making it more reliable than previous secondary retrospective aggregate data-based analysis [26,27].

There were three limitations. First, we captured the extent of use of ACF tool by ACF activity days (1031 of 1467 ACF activity days) and not based on what proportion of *Ni-kshay* reported ACF care cascade data were captured by the ACF tool. The reason was that most districts did not update their *Ni-kshay* ACF data for the study period till the completion of our analysis. Second, as we had project research assistants posted in the study districts, these findings could present a best-case scenario with the true picture being worse. Finally, this being analysis of routinely captured data, data collection errors cannot be ruled out. The research assistants tried to minimize this when they compiled the forms at district level.

Inference from our findings

To begin with, let us compare our findings with 2021 [26]. First, in 2021, based on secondary aggregate *Ni-kshay* data, nearly nine percent (cut-off 10%) of the population was reported to be screened indicating acceptable scale (Supplementary Table 1) [26]. However, the limitation here was that we were not sure whether those screened were from high-risk population [26]. Our estimates from this study, which are more reliable, suggest that only 7%

of mapped high-risk population were screened (cut-off 90%). Even if the ACF tool was used in all ACF days and if the mapped high-risk population was reduced to 11% (instead of 18.3% observed), the estimated scale of ACF will increase only to 17.5%, way lower than the target of 90%.

Second, in 2021, 1% of screened were tested (target 4.75%) [26]. Our findings in 2023 that provide information on all ACF quality indicators suggest that it is due to a combination of sub-optimal screening in the field (low percentage of presumptive among those screened) and losses between presumption, sputum collection and testing. Not capturing aggregate number of presumptive among screened in *Ni-kshay* could be the reason for identifying sub-optimal screening in the field during implementation monitoring.

Third, our findings estimate a low test positivity when compared to 2021 [26]. We speculate that due to non-capture of number of presumptive TB in *Ni-kshay*, percentage presumptive among screened and percentage tested among presumptive cannot be generated. Hence, all the focus in the field in 2021 might have been on test positivity. In the event of not using ACF tool in the field and the absence of mechanisms to cross-check ACF-detected TB (whether they are truly ACF-detected), it is difficult to cross-verify test positivity reported in 2021. In the past, performance of ACF was also being routinely assessed using two methods that focused on ACF-detected TB [32]: i) crude proportion of TB cases detected by PCF compared to ACF for a defined geographical area and period and ii) additional number of TB cases detected in a geographic area (before-during). These methods of monitoring have many limitations and should be done away with [32].

Overall, low scale makes ACF ineffective and high NNS makes ACF inefficient with already constrained resources. The concern of low yield due to scale and quality being below the benchmark has been repeatedly highlighted in previous assessments of NTEP [33].

By specific high-risk groups, the elderly and prison inmates in our study were an exception where ≥5% screened were identified as presumptive TB. NSS was within or around 1538 cut-off for prisons, contact tracing, non-communicable diseases and people who have recovered from COVID19. A systematic review (2006-19) has reported an NNS of 35 among contacts screened in low- and middle-income countries [8]. Another systematic review from low- and middle-income countries (1999-2020) has revealed that yield of ACF is high in prison, contacts, HIV, drug users and poor households [9]. By broad high-risk groups, geographically high-risk were screened the maximum in our study along with the highest NNS. The high NNS was due to lower percentage of screened identified as presumptive TB, lower test positivity and less use of rapid molecular diagnostic tests.

When compared to routine case-finding within NTEP (most being PCF), the test positivity in ACF care cascade was lower than PCF (1.6% versus 4.5%) [14]. The extent of use of rapid molecular diagnostic tests among ACF- and PCF-detected presumptive TB was similar (26% versus 23%) [14].

Implications for India's NTEP

In addition to recommendations from the first and second (qualitative) phase of the project published elsewhere [26,31], the implications from this study from third phase are as follows.

First, to explore in depth the reasons for ACF scale and quality being below the benchmark, a systematic qualitative enquiry is warranted. This has already been completed under the second phase of the TB ACF evaluation project (February to August 2023). Among NTEP staff at various levels, inadequate training, a significant knowledge gap and know-do gap was observed [31]. Six tips for improving scale and quality of ACF were recommended [31].

Second, more effective planning and resources are required if we are to cover 90% of the high-risk populations at least once a year. There is scope to screen a lot more than the current average of 565 screened per ACF activity day per district. In resource constrained settings, specific high-risk groups (prison, contacts) may be prioritized.

Third, continuous monitoring of various ACF quality indicators and increased access to rapid molecular diagnostic tests are recommended with a focus on geographically high-risk. To monitor percentage presumptive among screened and percentage tested among presumptive, aggregate number of presumptive TB should be captured against each ACF activity in the ACF module of *Ni-kshay*.

Fourth, our findings provide evidence in favour of using an ACF tool for individual level data collection in the field (see Supplementary Annex) so that reliable aggregate numbers can be reported in *Ni-kshay*. These can be randomly cross-checked. Additionally, ACF tool may be of use to disburse quality-control linked incentives [31]. Quality-control linked incentives should be for every household visited and every 'sputum collection and transport' (and not linked to number of ACF-detected person with TB) [34,35]. To initiate release of incentives for household visit and sputum collection and transport for a period, as recommended in phase two of the project, completed ACF tool should be submitted, with verification of a certain percentage of data (includes verifying all ACF-detected were detected as a result of ACF) and meeting a conservative test positivity or NNS cut-off [31]. This was successfully done in project *Axshya* (before 2017), where ACF was implemented by non-governmental organizations in India [34]. Decisions about the use of such approaches must be considered in the planning phase [35].

Finally, more resources and effective planning, use of ACF tool for individual level data collection in the field, continuous monitoring of various ACF quality indicators, increased

access to rapid molecular diagnostic tests and addressing the knowledge / know-do gap are applicable not only for India but also other high TB burden countries that plan to scale up ACF and improve quality in routine program settings. This is applicable irrespective of the ACF algorithm used. These planning and implementation barriers in attaining scale and quality, if unaddressed, will impact the attainment of optimal yield even in the context of adding hand held radiography with computer assisted diagnostics and rapid molecular tests for screening high-risk populations (in addition to symptom screen) [6]. The TB officers at district and state/province level, the national TB programs and their partners (non-Governmental organizations, funding agencies) supporting ACF activities should keep these in mind while planning and implementing ACF. As correctly stated by MacPherson P et al, while *ACF cannot and should not be a substitute for equitable access to responsive, affordable, accessible primary care services for all, with careful planning and substantial investment, ACF for TB can be an impactful approach to accelerating progress towards ending TB in high burden countries* [36].

CONCLUSION

In India, using prospective individual level data of TB ACF among high-risk populations in 2023, this study provided nationally representative estimates of all ACF scale and quality indicators, time taken and use of rapid molecular diagnostic tests. This study confirmed the previous findings of one cycle per year and ACF quality being below the benchmark (2021) [26]. Additionally, low scale of ACF was confirmed in our study. Individual level data collection of those screened facilitates implementation monitoring of routinely reported aggregate data in *Ni-kshay* ACF module, resulting in reliable indicators. Therefore, the findings of this study are more reliable and supersede the findings of 2021 that were based on

retrospective secondary aggregate data [26]. The use of rapid molecular tests among ACF-detected presumptive TB was similar to PCF-detected presumptive TB. To influence TB transmission, at least one ACF cycle a year should be implemented at scale and with good quality. More effective planning and resources including access to rapid molecular diagnostic tests are recommended.

Acknowledgements: The India central TB division commissioned, USAID/JSI-supported, ICMR-National Institute of Epidemiology (ICMR-NIE) led, national level TB ACF evaluation project was a collaboration among several key institutions: ICMR-National Institute of Epidemiology (ICMR-NIE) in Chennai, India, USAID India and JSI India, both based in New Delhi; The WHO Country Office for India, also located in New Delhi; and the Central TB Division, Ministry of Health and Family Welfare, Government of India, situated in New Delhi. The authors express sincere gratitude for the invaluable support and contributions from all state TB cells, district TB cells, and the WHO NTEP medical consultant network in India. Additionally, we acknowledge the significant contributions made by research assistants and interns from ICMR-NIE, Chennai, India. The research assistants facilitated data capture in the study districts, while the interns provided essential monitoring of project activities and vital data management support. We acknowledge the following research assistants (not included in the author list): Mr Chandan Kumar Mishra, Mr Deepak Kumar, Ms Santilahun Nongstein, Mr K Arul, Mr Gathala Subhash, Mr Debjyoti Chatterjee, Mr M Rajasekar, Mr Pandiyaraja, Mr Bnajingsuk Nahain Kyndail, Mr Jerry Hujon, Ms Daisy Daimary, Ms Divya Gupta, Ms Krishna Vaghela and Ms Khusbu Ahmas. We acknowledge the following ICMR-NIE interns: Ms. Divya V, Mr. Sathish Kumar, Ms. Suhana Khatoon, Dr. Kavya Rao Bagati, Ms. Lathika R, Ms. Alifia PS, Dr. Aishwarya Dhumale, Dr. Shwetha

R, Ms. Rebecca John, Ms. N Bhooma, Ms. B Suvetha, Mr. Mahesh Gomasa, Mr. Divakar G, Mr. Vishnu Bagavath, and Ms VK Damini. We acknowledge the support of KhushiBaby in supporting mobile-based ACF tool for use in the field (part of their Community Health Integrated Platform) in three districts of a state.

Contributors: HDS, GK, ANS, BV, VR, SKM, SI, DT, AC, SD, JC, KR, RR and MVM conceived and designed the study. HDS, SKP, PR, RS, RRV, PKH, AF, PS, BS, GunjanK, SKP, MAB, KVS, MS, AKD, DB, MLP, DYW, AK, PK, JSP, SushmitaD, AjitK, AjayK, SY, SC, SSM, AmitK, MP, SachinS, ST, PM, BB, GM, BM, AR, BKM, DNB, UCT and KUK supported in data collection and entry. RS, SKP and HDS developed the web application for data entry. HDS, SKP and PR did the project management and coordinated the operations. HDS and SKP managed the data. HDS and SKP did the data cleaning and analysis. HDS, SKP and PR prepared the first draft of the paper. All the authors were involved in data visualization. All authors contributed, reviewed and approved this article. HDS and SKP accessed and verified the data. HDS, SKP, RR and MVM had final responsibility for the decision to submit for publication.

Funding: This study was funded by USAID through John Snow International under the TB Implementation Framework Agreement (TB commitment grants 0011-0549-1024 and 0011-0549-1025 to MVM). All authors had full access to all the data and had final responsibility for the decision to submit for publication. All authors had full access to all the data and had final responsibility for the decision to submit for publication.

Disclaimer: The contents of this study document are the authors' sole responsibility and do not necessarily reflect the views of Central TB Division (India), World Health Organization, ICMR-NIE, JSI, other organizations authors are affiliated to, USAID or the United States Government.

Competing interest: There are no undeclared conflicts of interest.

Data availability statement: All the data are fully available without restriction

Figshare: The link contains a collection of the dataset (EpiData REC file), the codebook/data dictionary for dataset (MS Excel) and the programme file for analysis of dataset (EpiData PGM file) pertaining to this study. https://doi.org/10.6084/m9.figshare.c.7048385.v1

Data are available under the terms of the Creative Commons Attribution 4.0 International License (CC BY 4.0)

Supplementary material: ACF tool for individual level data collection in the field has been shared as Supplementary Annex. There are two supplementary tables and one supplementary figure.

Open access. This is an open access article distributed in accordance with the Creative Commons Attribution 4.0 Unported (CC BY 4.0) license, which permits others to copy, redistribute, remix, transform and build upon this work for any purpose, provided the original work is properly cited, a link to the licence is given, and indication of whether changes were made. See: https://creativecommons.org/licenses/by/4.0/.

ORCID IDs

Hemant Deepak Shewade https://orcid.org/0000-0002-8242-1911

REFERENCES

1. World Health Organization (WHO). Global Tuberculosis Report 2016. Geneva, Switzerland; 2016. Available: http://apps.who.int/iris/bitstream/10665/250441/1/9789241565394-eng.pdf?ua=1

2. World Health Organization (WHO). Global Tuberculosis Report 2023. Geneva, Switzerland; 2023. Available: https://www.who.int/publications/i/item/9789240083851

3. World Health Organization (WHO). Systematic screening for active tuberculosis: an operational guide. Geneva, Switzerland, Switzerland; 2015. Available: http://apps.who.int/iris/bitstream/10665/181164/1/9789241549172_eng.pdf?ua=1

4. Creswell J, Ottmani SE, Wei D, Sahu S, Lönnroth K, Uplekar MW. Programmatic approaches to screening for active tuberculosis. Int J Tuberc Lung Dis. 2013;17: 1248–1256. doi:10.5588/ijtld.13.0199

5. Lönnroth K, Corbett E, Golub J, Godfrey-Faussett P, Uplekar M, Weil D, et al. Systematic screening for active tuberculosis: rationale, definitions and key considerations [State of the art series. Active case finding/screening. Number 1 in the series]. Int J Tuberc Lung Dis. 2013;17: 289–298. doi:10.5588/ijtld.12.0797

6. World Health Organization. World Health Organization (WHO) consolidated guidelines on tuberculosis. Module 2: Screening-Systematic screening for tuberculosis disease. Geneva, Switzerland; 2021.

7. Pramono JS, Ridwan A, Maria IL, Syam A, Russeng SS, Syamsuar, et al. Active Case Finding for Tuberculosis in Migrants: a Systematic Review. Med Arch (Sarajevo, Bosnia Herzegovina). 2024;78: 60–64. doi:10.5455/medarh.2024.78.60-64

8. Velleca M, Malekinejad M, Miller C, Abascal Miguel L, Reeves H, Hopewell P, et al. The yield of tuberculosis contact investigation in low- and middle-income settings: a systematic review and meta-analysis. BMC Infect Dis. 2021;21: 1011. doi:10.1186/s12879-021-06609-3

9. Bohlbro AS, Hvingelby VS, Rudolf F, Wejse C, Patsche CB. Active case-finding of tuberculosis in general populations and at-risk groups: a systematic review and meta-analysis. Eur Respir J. 2021;58: 2100090. doi:10.1183/13993003.00090-2021

10. Haeusler IL, Torres-Ortiz A, Grandjean L. A systematic review of tuberculosis detection and prevention studies in prisons. Glob Public Health. 2022;17: 194–209. doi:10.1080/17441692.2020.1864753

11. Gogichadze N, Sagrera A, Vicente JÁ, Millet J-P, López-Seguí F, Vilaplana C. Cost-effectiveness of active tuberculosis screening among high-risk populations in low tuberculosis incidence countries: a systematic review, 2008 to 2023. Euro Surveill. 2024;29: 2300614. doi:10.2807/1560-7917.ES.2024.29.12.2300614

12. World Health Organization (WHO). Optimizing active case-finding for tuberculosis. Geneva, Switzerland; 2021.

13. Burke RM, Nliwasa M, Feasey HRA, Chaisson LH, Golub JE, Naufal F, et al. Community-based active case-finding interventions for tuberculosis: a systematic review. Lancet Public Heal. 2021;6: e283–e299. doi:10.1016/S2468-2667(21)00033-5

14. Central TB Division. Ministry of Health and Family Welfare. Government of India. India TB Report 2023. New Delhi, India; 2023.

15. Garg T, Chaisson LH, Naufal F, Shapiro AE, Golub JE. A systematic review and meta-analysis of active case finding for tuberculosis in India. Lancet Reg Heal - Southeast Asia. 2022;7: 100076. doi:10.1016/j.lansea.2022.100076

16. Leo S, Rajaram M, Mohapatra MM, Chinnakali P, Joseph NM, Vemuri MB. Active Case Finding for Tuberculosis among Health Care Workers in a Teaching Hospital, Puducherry, India. Indian J Occup Environ Med. 2023;27: 42–48. doi:10.4103/ijoem.ijoem_342_21

17. Chawla S, Gupta V, Gour N, Grover K, Goel PK, Kaushal P, et al. Active case finding of tuberculosis among household contacts of newly diagnosed tuberculosis patients: A community-based study from southern Haryana. J Fam Med Prim care. 2020;9: 3701–3706. doi:10.4103/jfmpc.jfmpc_532_20

18. Narayan G, Vasudevan K, Rajagopal A, Gunasekaran K, Savla H, Suvvari TK. Active Screening for Pulmonary Tuberculosis Among Jail Inmates: A Mixed Method Study From Puducherry, South India. Cureus. 2023;15: e39749. doi:10.7759/cureus.39749

19. Shukla G, Sarin R, Vohra V, Khayyam KU. To study the impact of active case finding (ACF) among the TB patients detected in South Delhi. Indian J Tuberc. 2021;68: 80–84. doi:10.1016/j.ijtb.2020.08.011

20. Shriraam V, Srihari R, Gayathri T, Murali L. Active case finding for Tuberculosis among migrant brick kiln workers in South India. Indian J Tuberc. 2020;67: 38–42. doi:10.1016/j.ijtb.2019.09.003

21. Vyas A, Creswell J, Codlin AJ, Stevens R, Rao VG, Kumar B, et al. Community-based active case-finding to reach the most vulnerable: tuberculosis in tribal areas of India. Int J Tuberc Lung Dis. 2019;23: 750–755. doi:10.5588/ijtld.18.0741

22. Dey A, Thekkur P, Ghosh A, Dasgupta T, Bandopadhyay S, Lahiri A, et al. Active Case Finding for Tuberculosis through TOUCH Agents in Selected High TB Burden Wards of Kolkata, India: A Mixed Methods Study on Outcomes and Implementation Challenges. Trop Med Infect Dis. 2019;4: 134. doi:10.3390/tropicalmed4040134

23. Vijayan S, Jondhale V, Pande T, Khan A, Brouwer M, Hegde A, et al. Implementing a chest X-ray artificial intelligence tool to enhance tuberculosis screening in India: Lessons learned. PLOS Digit Heal. 2023;2: e0000404. doi:10.1371/journal.pdig.0000404

24. Prasad BM, Satyanarayana S, Chadha SS, Das A, Thapa B, Mohanty S, et al. Experience of active tuberculosis case finding in nearly 5 million households in India. Public Heal Action. 2016;6: 15–18. doi:10.5588/pha.15.0035

25. Prasad BM, Thapa B, Chadha SS, Das A, Babu ER, Mohanty S, et al. Status of Tuberculosis services in Indian Prisons. Int J Infect Dis. 2017;56: 117–121. doi:10.1016/j.ijid.2017.01.035

26. Shewade HD, Kiruthika G, Ravichandran P, Iyer S, Chowdhury A, Kiran Pradeep S, et al. Quality of active case-finding for tuberculosis in India: a national level secondary data analysis. Glob Health Action. 2023;16: 2256129. doi:10.1080/16549716.2023.2256129

27. Nagaraja SB, Thekkur P, Satyanarayana S, Tharyan P, Sagili KD, Tonsing J, et al. Active case finding for tuberculosis in india: A syntheses of activities and outcomes reported by the national tuberculosis elimination programme. Tropical Medicine and Infectious Disease. 2021. p. 206. doi:10.3390/tropicalmed6040206

28. Central TB Division. Ministry of Health and Family Welfare. Govt of India. Active Case Finding. Guidance Document (Updated in August 2019 by State TB Cell Haryana for ACF campaign 2019). New Delhi, India; 2019.

29. Central TB Division. Ministry of Health and Family Welfare. Government of India. Active Case Finding. Guidance Document. New Delhi, India; 2017.

30. National TB Elimination Programme (NTEP) India. Nikshay Reports. 2020 [cited 8 May 2022]. Available: https://reports.nikshay.in/Reports/TBNotification

31. Shewade HD, Ravichandran P, Pradeep SK, Kiruthika G, Shanmugasundaram D, Chadwick J, et al. Bridging the “know-do” gap to improve active case finding for tuberculosis in India: A qualitative exploration into national tuberculosis elimination program staffs’ perspectives. Goli A, editor. PLoS One. 2024;19: e0309750. doi:10.1371/journal.pone.0309750

32. Satyanarayana S, Nagaraja SB. Measuring the performance of active TB case finding: programme managers need to be cautious. Public Heal Action. 2017;7: 178–178. doi:10.5588/pha.17.0023

33. Central TB Division; Government of India. Report of the joint monitoring mission, revised national tuberculosis control programme, November 2019. New Delhi, India; 2019. Available: https://www.indiaspend.com/wp-content/uploads/2020/06/Report-of-JMM-RNTCP-Nov-2019_Mobile.pdf

34. Shewade HD, Gupta V, Satyanarayana S, Pandey P, Bajpai UN, Tripathy JP, et al. Patient characteristics, health seeking and delays among new sputum smear positive TB patients identified through active case finding when compared to passive case finding in India. PLoS One. 2019;14: e0213345. doi:10.1371/journal.pone.0213345

35. Singh AA, Creswell J, Bhatia V. Framework for planning and monitoring active TB case finding interventions to meet the global targets in the COVID-19 era and beyond: South-East Asia perspective. PLOS Glob public Heal. 2021;1: e0000073. doi:10.1371/journal.pgph.0000073

36. MacPherson P, Shanaube K, Phiri MD, Rickman HM, Horton KC, Feasey HRA, et al. Community-based active-case finding for tuberculosis: navigating a complex minefield. BMC Glob Public Heal. 2024;2. doi:10.1186/s44263-024-00042-9

FIGURE 1. Scale and quality indicators for an ACF cycle for TB, India, as per the national ACF guidance 2019 [28]

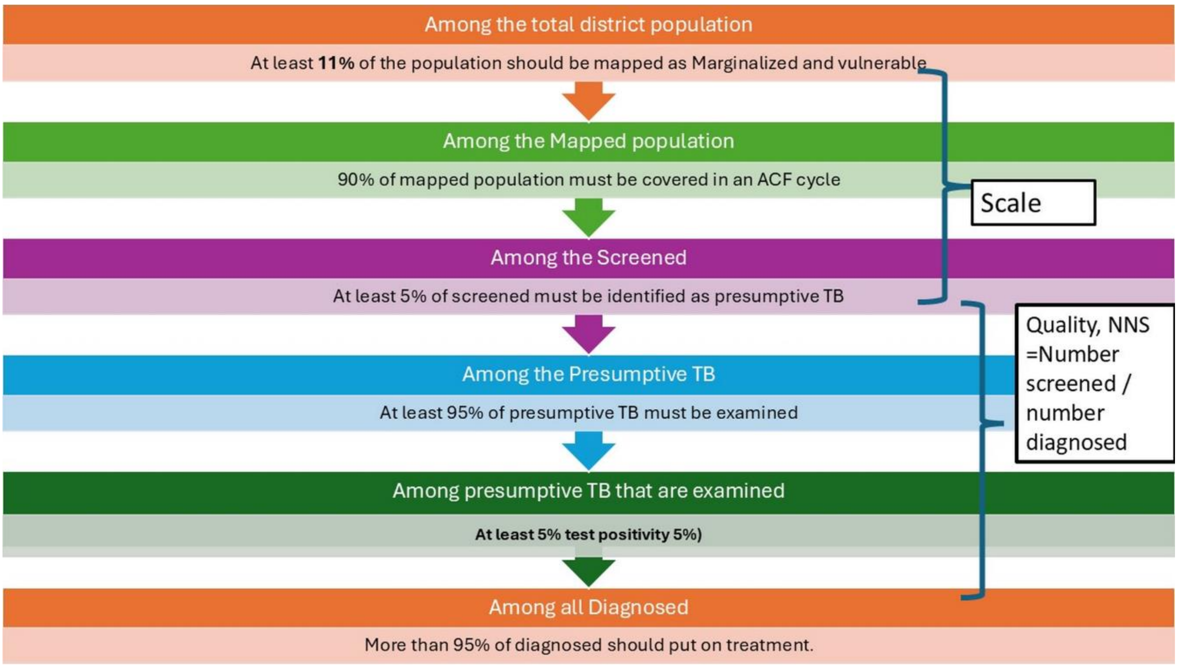

Abbreviations: ACF, active case-finding; TB, tuberculosis

Reprinted with permission from Shewade HD et al [26] under a CC BY license

FIGURE 2. Scale and quality indicators in the TB ACF cycle conducted from January to September 2023 in 30 randomly sampled NTEP districts of India, overall and stratified by specific high-risk groups [a]

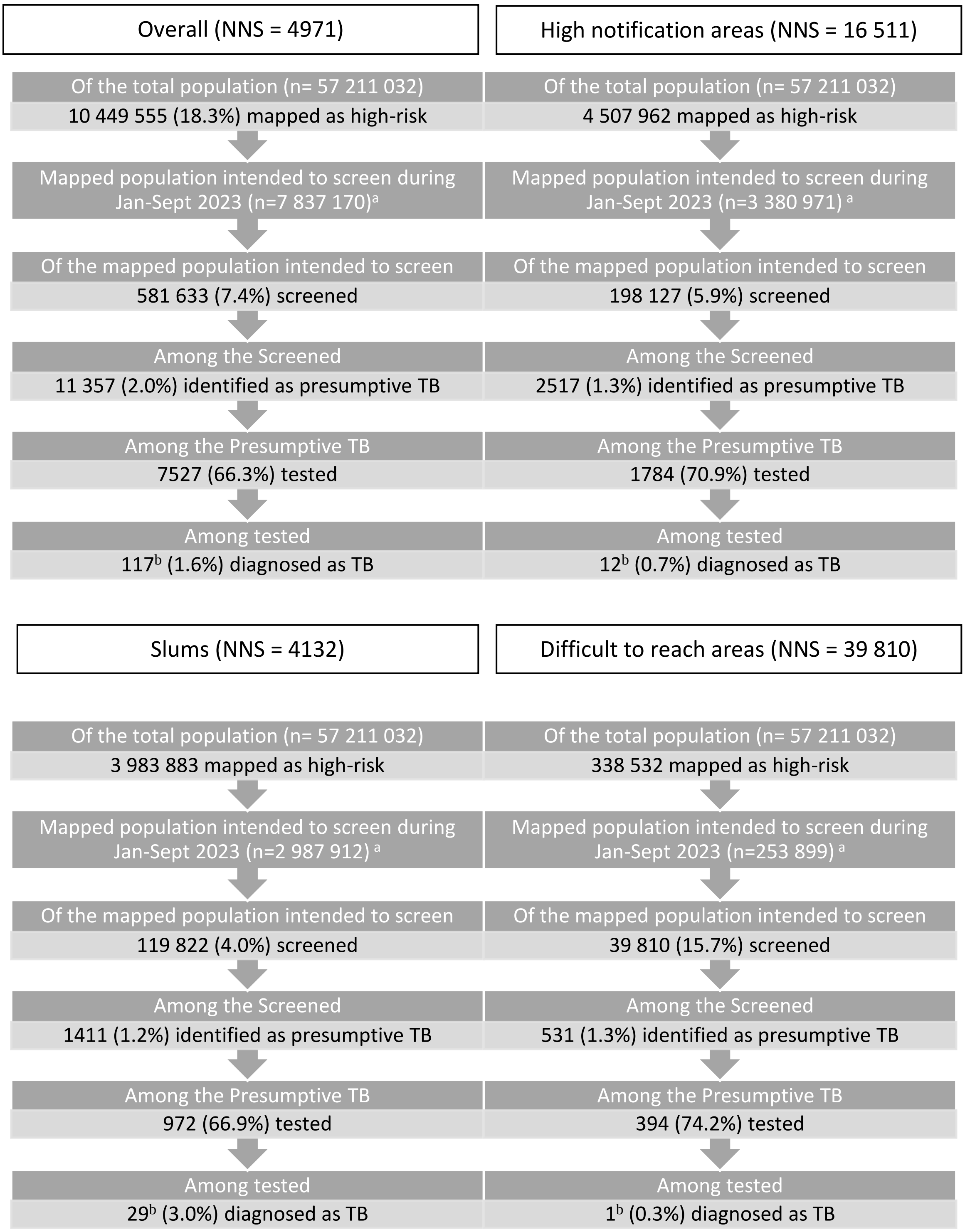

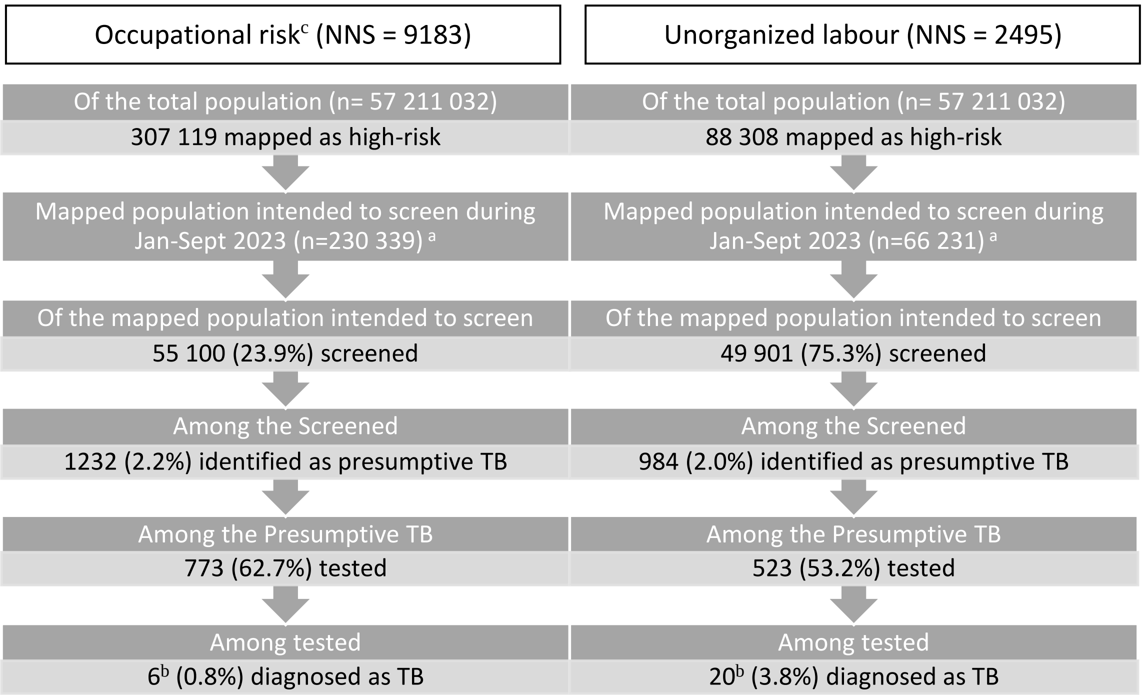

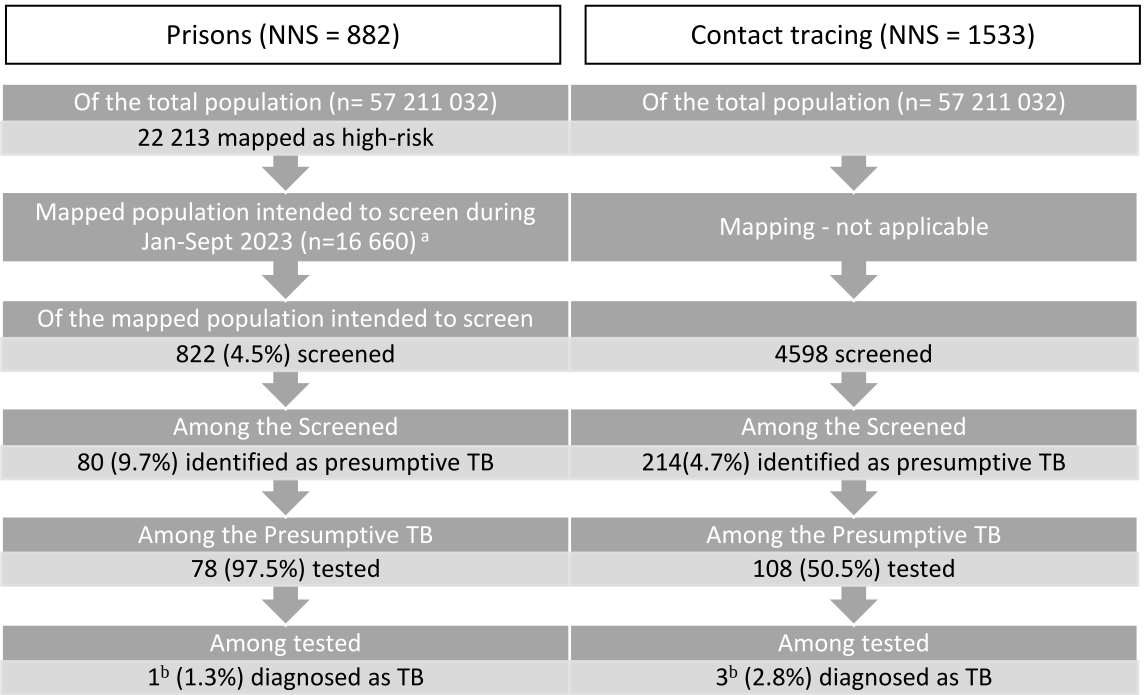

Abbreviations: ACF, active case-finding; TB, tuberculosis; NNS, number needed to screen (number screened divided by number diagnosed)

[a]75% of the mapped population was used as denominator as we captured ACF data for three-quarters of the year (one year=one ACF cycle); [a]Among screened, specific high-risk groups with 2.5% or higher contribution have been depicted. Data of prisons and contact screening has been included due to their importance; [b]all the ACF-detected TB were initiated on treatment; [c]occupation included construction site workers, cotton mill workers, mine workers, stone crushers, tea garden workers, weaving glass industrial workers, poultry workers, beedi workers

TABLE 1. Specific high-risk populations for active case-finding for TB classified[a] into three broad categories

**Clinical**	**Socioeconomic**	**Geographical (related to health access)**
• Contacts of people with TB<br>• Undernutrition<br>• HIV<br>• High risk groups for HIV identified by the program<br>• Noncommunicable diseases [Diabetes, hypertension, other cardiovascular diseases, chronic lung/liver/renal disease and their risk factors (alcohol, smoking, drug addiction, others)]<br>• People who have recovered from COVID19<br>• Immunocompromised (cancer), on palliative care<br>• Occupational risk with TB (construction site workers, weaving/glass industry workers, miners, cotton mill workers, stone crushers, beedi rolling, poultry workers, tea garden workers, health care workers)<br>• Elderly	• Prisons<br>• Old age homes, night shelters, home for destitute, orphanages, refugee camps, asylums<br>• Street children / homeless<br>• Unorganized labour<br>• Migrant population<br>• Slums<br>• Areas with high level of poverty / people living below the poverty line<br>• Populations known to drink raw milk/uncooked meat<br>• Indigenous/ tribal population<br>• Sanitary workers	• Hard to reach areas<br>• Villages seeking care from traditional healers<br>• Areas with clustering of TB<br>• Areas with low TB notification (than expected)

Abbreviations: TB, tuberculosis; HIV, human immunodeficiency virus; COVID19, corona virus disease 2019

[a]these specific high-risk groups may also be classified into facility-based and population-based, facility-based included orphanages, prison, refugee camps, homes for the destitute, tribal school homes, old age homes, night shelters, construction site workers, cotton mill workers, mine workers, stone crushers, tea garden workers, weaving, glass workers, industrial workers, poultry workers, beedi workers

TABLE 2. Quality indicators in the TB ACF cycle conducted from January to September 2023 in 30 randomly sampled NTEP districts of India, overall and stratified by gender, age, residence, and type of high-risk groups (broad categories)

Category		Screened		Presumptive TB		Tested		Diagnosed[a]		NNS
		**n**	**(%)[c]**	**n**	**(%)[r]**	**n**	**(%)[r]**	**n**	**(%)[r]**	
**Total**		**581 633**	**(100.0)**	**11 357**	**(2.0)**	**7527**	**(66.3)**	**117[a]**	**(1.6)**	**4971**
Gender	Male	293260	(50.4)	5798	(2.0)	3961	(68.3)	90	(2.3)	3258
	Female	284612	(48.9)	5526	(1.9)	3549	(64.4)	27	(0.8)	9208
	Other	48	(<0.1)	1	(2.1)	1	(100.0)	0	(0.0)	-
	Missing	3713	(0.6)	32	(0.9)	16	(50.0)	0	(0.0)	-
Elderly	Yes (>60y)	63645	(10.9)	3311	(5.2)[b]	2258	(68.2)	32	(1.4)	1989
	No	512 127	(88.0)	8007	(1.6)[ref]	5239	(65.4)	85	(1.6)	6025
	Missing	5861	(1.0)	30	(0.5)	30	(100.0)	0	(0.0)	-
Residence	Rural	349300	(60.1)	7590	(2.2)	4994	(65.8)	57	(1.1)	6128
	Urban	232300	(39.9)	3766	(1.6)	2532	(67.2)	60	(2.4)	3872
	Missing	33	(<0.1)	1	(3.0)	1	(100.0)	0	(0.0)	-
Type of high-risk population										
	Socioeconomically	188 961	(32.5)	2893	(1.5)	1910	(66.0)	56	(2.9)[b]	3374
	Clinically	88 804	(15.3)	3621	(4.1)[b]	2504	(69.2)	26	(1.0)	3416
	Geographically	238 297	(41.0)	3049	(1.3)[ref]	2178	(71.4)	13	(0.6)[ref]	18331
	Not classifiable[c]	63 011	(10.8)	1794	(2.8)	935	(52.1)	22	(2.4)	2864
Facility-based high-risk group										
	Yes	55 743	(9.6)	1399	(2.5)	915	(65.4)	8	(0.9)	6968
	No	501 619	(86.2)	8832	(1.8)	5976	(67.7)	93	(1.6)	5394
	Not classifiable	24 271	(4.2)	1126	(4.6)	636	(56.5)	16	(2.5)	1517

Abbreviations: TB, tuberculosis; ACF, active case-finding; NNS, number needed to screen

[a]all the ACF-detected TB were initiated on treatment

[b]public health wise significant difference (at least 10% for proportion of presumptive tested, at least 2% for other proportions) which was statistically significant ($p<0.05$ using chi squared test) was considered

[c]there were many instances where a mapped population could be classified into multiple specific high-risk population types and these specific high-risk population types could be from more than one broad high-risk population types

[c]column percentage; [r]row percentage; [ref]Reference

TABLE 3. Use of rapid molecular diagnostic test among ACF-detected presumptive TB who were tested in 30 randomly sampled NTEP districts of India, January to September 2023, overall and stratified by gender, age, residence and type of high-risk groups (broad categories)

**Category**		**Tested**	**Rapid molecular diagnostic test**	
		**N**	**n**	**(%)[r]**
**Total**		**7527**	**1989**	**(26.4)**
Gender	Male	3961	1150	(29.0)
	Female	3549	835	(23.5)
	Other	1	1	(100.0)
	Missing	16	3	(18.8)
Elderly	Yes (>60y)	2258	657	(29.1)
	No	5239	1329	(25.4)
	Missing	30	3	(10.0)
Residence	Rural	4994	902	(18.1) [a]
	Urban	2532	1087	(42.9)[ref]
	Missing	1	0	(0.0)
Type of high-risk population				
	Socioeconomically	1910	784	(41.0)[ref]
	Clinically	2504	783	(31.3)[a]
	Geographically	2178	114	(5.2) [a]
	Not classifiable[b]	935	308	(32.9)
Facility-based high-risk group				
	Yes	915	395	(43.2)[ref]
	No	5976	1340	(22.4)[a]
	Not classifiable	636	254	(39.9)

Abbreviations: TB, tuberculosis; ACF, active case-finding; NNS, number needed to screen
[a]difference in proportion of at least 10% and statistically significant ($p<0.05$) using chi-squared test
[b]there were many instances where a mapped population could be classified into multiple specific high-risk population types and these specific high-risk population types could be from more than one broad high-risk population types
[r]row percentage; [ref]Reference

Figure 1

Click here to access/download;Figure;Figure 1_PLOS.tif

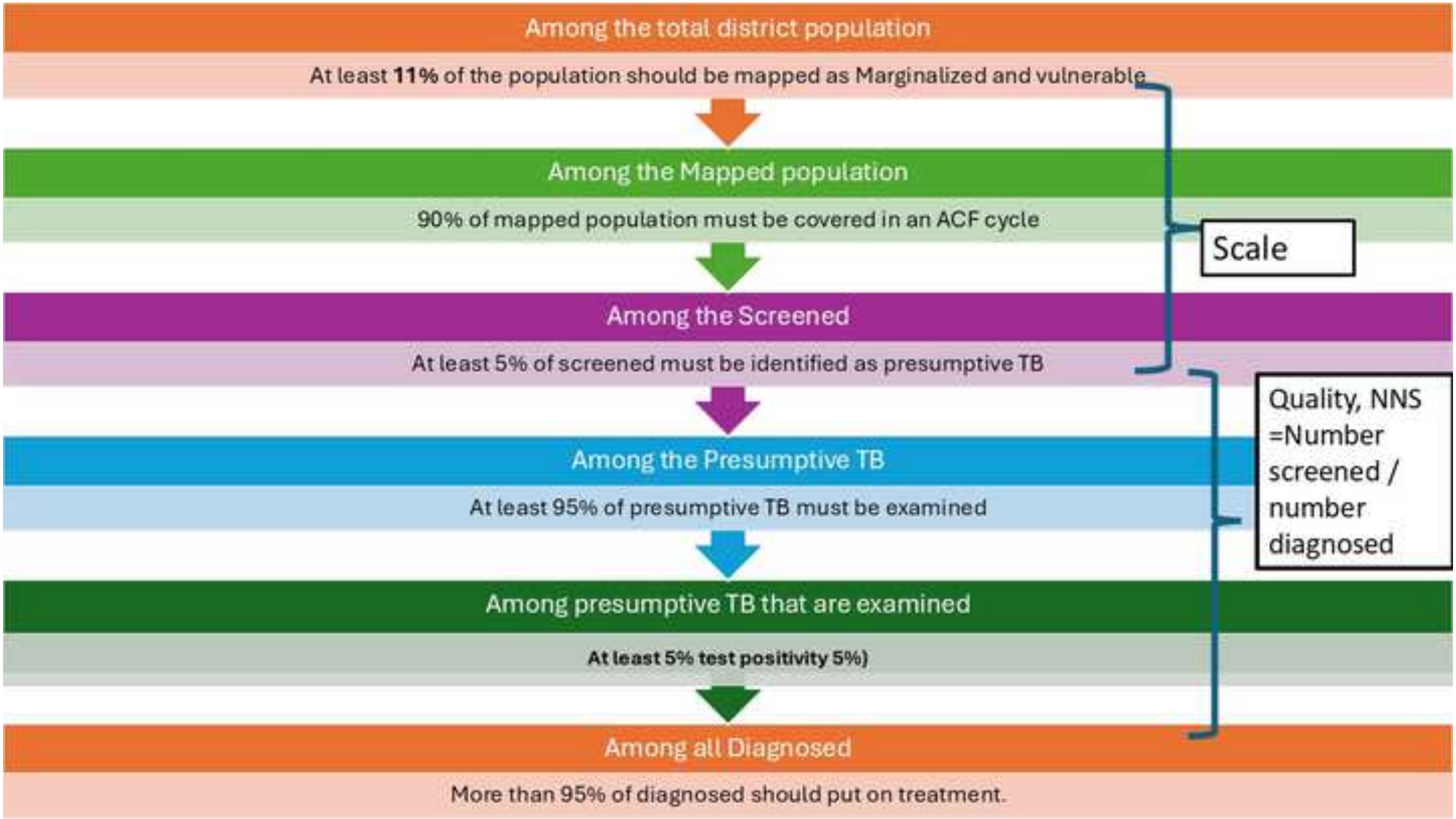

Figure 2

Click here to access/download;Figure;Figure 2_PLOS.tif

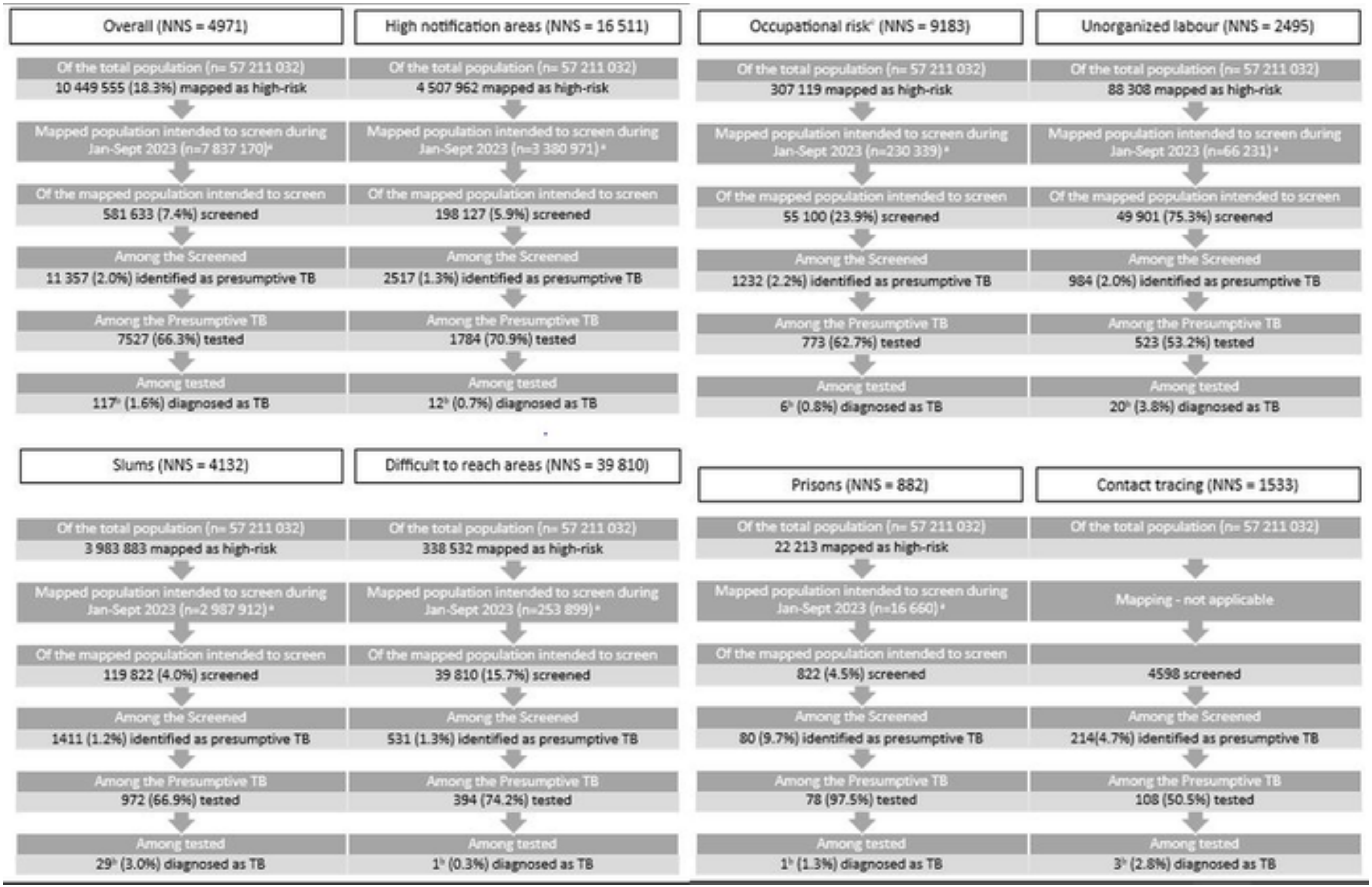

Supplementary figure 1, Supplementary tables 1 and 2

Click here to access/download

Supporting Information

Supplementary Tables and Figure_PLOS.docx

Supplementary annex 1

Click here to access/download
Supporting Information
Supplement Annex_plos.pdf

ORIGINAL ARTICLE

Active case-finding for TB in India: Assessment of scale and quality benchmarks, time taken and use of rapid molecular diagnostic tests

Hemant Deepak Shewade[a,†,*], S Kiran Pradeep[a,†], Prabhadevi Ravichandran[a], G Kiruthika[a], Amar N Shah[b], Bhavin Vadera[b], R Sabarinathan[a], Venkatesh Roddawar[c], Sanjay K Mattoo[d], Swati Iyer[e], Dheeraj Tumu[e], Aniket Chowdhury[e], Shanmugasundaram Devika[a], Joshua Chadwick[a], Rakesh R Vaidya[f], Prasanta Kumar Hota[g], Asha Frederick[h], Pankaj Singh[i], Bayarilin Shanpru[j], Gunjan Khunger[k], Subrata Kumar Panda[e], Mohamed Arif Baig[e], K V Suma[e], Mahendran Suleka[a], Amit Kumar Digal[a], Debobrata Banerjee[a], Machupalli Lakshmi Prasanna[a], Divya Yashpal Waghela[a], A Krishnaraj[a], Pratibha Kashyap[a], Jayeshbhai Sendhabhai Parmar[a], Sushmita Das[a], Ajit Kumar[a], Ajay Kumar[a], Sunny Yadav[a], Shradha Chetri[a], Subhashree Suchismita Mahapatra[a], Amit Kumar[a], Monu Pathak[a], Sachin Singh[a], Shams Tabrez[a], Piyush Mehra[a], Bipra Bishnu[e], G Mahesh[e], Biswabihari Mohanty[l], A Rajesham[m], Bal Krishna Mishra[n], Dinesh N Barot[o], Umesh Chandra Tripathi[e], Khalid Umer Khayyam[p], Kiran Rade[e,@], Raghuram Rao[d,@], Manoj V Murhekar[a,@]

[a]ICMR-National Institute of Epidemiology (ICMR-NIE), Chennai, India

[b]USAID India, New Delhi, India

[c]John Snow India Private Limited, New Delhi, India

[d]Central TB Division, Ministry of Health and Family Welfare, New Delhi, India

[e]Office of the World Health Organization (WHO) Representative to India, WHO Country Office, New Delhi, India

[f]State TB Cell, Government of Gujarat, Ahmedabad, India

[g]State TB Cell, Government of Odisha, Bhubaneshwar, India
[h]State TB Cell, Government of Tamil Nadu, Chennai, India
[i]State TB Cell, Government of Uttarakhand, Dehradun, India
[j]East Khasi Hills District TB Cell, Government of Meghalaya, Shillong India
[k]Ganganagar District TB Cell, Government of Rajasthan, Ganganagar, India
[l]Anugul District TB Cell, Government of Odisha, Anugul ,India
[m]State TB Cell, Government of Telangana, Hyderabad, India
[n]State TB Cell, Government of Bihar, Patna, India
[o]Kheda District TB Cell, Government of Gujarat, Nadiad, India
[p]National Institute of Tuberculosis and Respiratory Diseases, Government of India, New Delhi, India
[†]joint primary authors; [@]joint senior authors

**Correspondence to:* Dr Hemant Deepak Shewade, MBBS MD PhD, Scientist E, Division of Health Systems Research, ICMR-National Institute of Epidemiology (ICMR-NIE), Chennai 600077, India. E-mail: hemantjipmer@gmail.com)

Short title: ACF for TB in India (2023)

Key words: India; Operational research; TB ACF cycle; TB ACF quality indicators; high-risk groups; number needed to screen.

Manuscript details

Word count: Abstract (299~~2~~ words); main text (4273~~195~~ words); 2 figures; 3 tables; 1 supplementary figure; 2 supplementary tables; 1 supplementary annex; 36~~7~~ references

ABSTRACT

Since 2017, tuberculosis active case-finding (TB ACF) in India is being implemented in routine national TB elimination program settings. Symptom screen of high-risk population followed by confirmation of TB among symptom-screen positive is the TB ACF algorithm. TB ACF scale and quality assessments hitherto were predominantly local or based on aggregate program data with limited details on all the indicators, the extent of use of rapid molecular diagnostic tests and the time taken. As a part of the national TB elimination program commissioned evaluation, we assessed one ACF cycle (intention to screen the high-risk population once) between January and September 2023 using nationally representative prospectively collected individual level data. In this cohort study from high-risk populations in 30 randomly sampled districts (nine states), 581, 633 high-risk individuals were screened by the program utilizing existing workforce and resources. The two scale indicators (target, observed) were: percentage of i) population mapped as high-risk (≥11%, 18.3%) and ii) mapped population screened (≥90%, 7.4%). The four quality indicators (target, observed) were: percentage of i) screened identified as presumptive TB (≥5%, 2%) ii) presumptive TB tested (≥95%, 66.3%) iii) tested diagnosed as TB (≥5%, 1.6%) and iv) diagnosed put on treatment (≥95%, 100%). The number needed to screen (target) to detect one person with TB was 4971 (≤1538 considering the algorithm). This was observed across most of the high-risk groups, with few exceptions. The extent of using rapid molecular diagnostic tests was 26.4% and the median time taken from screening to sputum collection and testing was one day. To conclude, ACF scale and quality were grossly below the benchmark, lower than previously reported (2021) based on retrospective aggregate program data. Effective planning, resource allocation including use of rapid molecular diagnostic tests and individual data recording among those screened to facilitate implementation monitoring are recommended.

AUTHOR SUMMARY

In India, ~~TB ACF~~tuberculosis active case-finding scale and quality assessments hitherto were predominantly local or based on aggregate program data with limited details on all the indicators and the extent of use of rapid molecular diagnostic tests for confirmation of ~~TB~~tuberculosis. We used nationally representative prospectively collected individual level ~~ACF~~ data. In 2023, scale and quality of active case-finding for ~~TB~~tuberculosis was below the benchmark, lower than previously reported (2021) based on retrospective aggregate program data. Among active case-finding-detected presumptive ~~TB~~tuberculosis, rapid molecular diagnostic tests were used in one-fourth (similar extent of use in passive case-finding). We recommend effective planning and resources (including access to rapid molecular diagnostic tests) to improve scale and quality. Individual data recording among those screened should be routinely done to facilitate implementation monitoring.

INTRODUCTION

Globally, to detect people with tuberculosis (TB), national TB programs have relied heavily on passive case-finding (PCF) and its impact has been less than adequate [1–3]. To detect the missing people with TB (3.1 million in 2022) and put them on treatment, other case-finding strategies are employed [4,5]. Active case-finding (ACF) is systematic screening for TB disease implemented outside of health facilities. This includes household contact investigation and community-based systematic screening of high-risk populations [1]. World Health Organization recommends conducting ACF in high-risk populations that have at least 0.5% prevalence of undetected TB and not in general population [5].

Many systematic reviews have synthesized information regarding effectiveness of ACF in high-risk populations overall and also in migrants, household contacts and prisons [6–9]. Even in low TB incidence countries, targeting high-risk populations has been found to be a cost-effective strategy [10]. The impact of ACF on TB transmission depends on the frequency of ACF cycles per year (number of times high-risk population are screened in a year) and yield of an ACF cycle (extent of estimated undetected TB in the high-risk population that is detected because of ACF). Yield of an ACF cycle depends on the choice of high-risk populations, the scale (proportion of high-risk population in a geographic area screened) and quality (NNS – number needed to screen) of an ACF cycle, and use of sensitive screening algorithms [11,12]. Most studies focus on quality (NNS) and number needed to test (test-positivity). There are limited studies that describe frequency, scale and yield.

Globally, India has the highest TB burden with 0.5 million missing TB in 2022 [4,13]. COVID19 pandemic impacted TB programs worldwide and reversed some of the gains made by India [14]. When compared to 2019, India’s TB case notifications fell by 25% in 2020 [15]. As a part of COVID19 mitigation measures, starting July 2021, there was a push to

effectively implement ongoing ACF among high-risk populations. By 2022, the annual TB notifications (2.4 million) reached the pre-COVID19 levels [13,16].

In 2022, India's National TB Elimination Program (NTEP) commissioned a national level TB ACF evaluation project (August 2022 to February 2024) to guide evidence-based strategic planning. Under the first phase of this project, we assessed the frequency, scale and quality of ACF at district, state and national level using retrospective secondary aggregate ACF data for the year 2021 from *Ni-kshay* (a case-based, web-based electronic TB information management system under NTEP) [17]. Data on high-risk populations, ACF-detected presumptive TB and ACF-detected TB initiated on treatment were not available to calculate all the scale and quality indicators (see Figure 1 for all program-recommended indicators) [17]. Hence, we used three revised indicators based on the available data for 2021. The details of these revised indicators and rationale for revision are given in Supplementary Table 1 [17]. Most of the states implemented one ACF cycle, and none of the states or districts met all the three revised indicator cut-offs (Supplementary Table 1) [17]. Though ≈9% (revised target 10%) of district population was reported to be screened in 2021, we were not sure whether these were high-risk populations [17]. Among those screened, ACF quality was largely below the benchmark (high NNS) [17].

Available studies on the ACF care cascade from India have three limitations; either they are local with limited scope for extrapolating the findings nationally [18–25], or are based in project settings [24–27], or based on aggregate retrospective secondary data [17,28]. We have limited data on all the indicators, the time taken, and the extent of use of rapid molecular diagnostic tests in the care cascade [17,18,27,28,19–26]. A systematic review (2022) revealed that ACF implementation is highly variable in India and current literature is sparse for many important risk groups [29].

~~To better understand the routinely implemented ACF, a~~Globally, we have made significant progress in reducing the missing people with tuberculosis (TB) [1,2]. To detect people with TB, national TB programs have relied heavily on passive case-finding (PCF) [3–5]. To detect the missing people with TB (3.1 million in 2022) and put them on treatment, other case-finding strategies are employed [2,6]. Active case-finding (ACF) is systematic screening for TB disease implemented outside of health facilities. This includes household contact investigation and community-based systematic screening of high-risk populations [3]. World Health Organization recommends conducting ACF in high-risk populations that have at least 0.5% prevalence of undetected TB and not in general population [6].

Many systematic reviews have synthesized information regarding effectiveness of ACF in high-risk populations overall and also in migrants, household contacts and prisons [7–10]. Even in low TB incidence countries, targeting high-risk populations has been found to be a cost-effective strategy [11]. The effectiveness of ACF on TB transmission depends on the frequency of ACF cycles per year (number of times high-risk population are screened in a year) and yield of an ACF cycle (extent of estimated undetected TB in the high-risk population that is detected because of ACF). Yield of an ACF cycle depends on the choice of high-risk populations, the scale (proportion of high-risk population in a geographic area screened) and quality (NNS - number needed to screen) of an ACF cycle, and use of sensitive screening algorithms [12,13]. Most studies assessing ACF care cascade focus on quality (NNS) and number needed to test (test-positivity). There are limited studies that describe frequency, scale and yield.

Globally, India has the highest TB burden with 0.5 million missing TB in 2022 [2,14]. A systematic review (2022) revealed that ACF implementation is highly variable in India and current literature is sparse for many important risk groups [15]. Additionally, studies

focussing on ACF care cascade from India have three limitations; either they are local with limited scope for extrapolating the findings nationally [16–23], or are based in project settings [22–25], or based on aggregate retrospective secondary data [26,27]. We have limited data on all the scale and quality indicators, the time taken, and the extent of use of rapid molecular diagnostic tests in the care cascade [16–27].

In 2022, India's National TB Elimination Program (NTEP) commissioned a national level TB ACF evaluation project (August 2022 to February 2024) to guide evidence-based strategic planning. Under the first phase of this project, we assessed the frequency, scale and quality of ACF at district, state and national level for the year 2021 using retrospective secondary aggregate ACF data from *Ni-kshay* (a case-based, web-based electronic TB information management system under NTEP) [26]. Data on high-risk populations, ACF-detected presumptive TB and ACF-detected TB initiated on treatment were not available to calculate all the scale and quality indicators (see Figure 1 for all program-recommended indicators). Hence, we used three revised indicators based on the available data (see Supplementary Table 1) [26]. The findings revealed that most of the states implemented one ACF cycle, with suboptimal scale and quality (Supplementary Table 1) [26]. Though ≈9% (revised target 10% for scale) of district population was reported to be screened, we were not sure whether these were high-risk populations [26].

A nationally representative prospective study involving individual-level data from ACF ~~cycles~~cycle with reliable estimates of all scale and quality indicators (see Figure 1) was required. Recording individual data of those screened ~~provides an opportunity for~~allows random checks of the aggregate numbers reported in ACF care cascade (facilitates implementation monitoring~~), resulting in generation of more reliable scale and quality indicators.~~). This would add to our existing knowledge and suggest modifications required in

the national ACF guidance ~~[30,31].~~[28,29]. Hence, under the third phase of the NTEP commissioned national level TB ACF evaluation project, we ~~determined~~estimated at national level (2023), i) all ACF scale and quality indicators, overall and stratified by key high-risk populations ii) the time taken in the care cascade and iii) the extent of use of rapid molecular diagnostic tests among ACF-detected people with presumptive TB.

METHODS

Study design

This was a cohort study using individual level secondary data.

Study setting

India has 36 state-level TB cells and 768 NTEP districts. At the national level, the central TB division provides guidance and leadership to state and district TB cells ~~[13].~~[14]. A district programme coordinator supports the district TB officer. In public sector, the lowest level of peripheral health ~~institutions (PHIs) that facilities~~ institution (PHI) facilitating TB diagnosis ~~are~~is the primary health ~~centres~~centre that ~~have~~has at least one medical doctor and ~~TB diagnostic centre, each with a~~ laboratory technician for sputum examination. Paper-based registers maintained at PHIs and sub-district level administrative units (TB units) have a line-list of people with TB notified, their management, and the treatment outcomes. Each TB unit has a senior TB treatment supervisor who ~~updates these details from the register in *Ni-kshay* using his/her mobile phone/ tablet [32].~~transcribes these details in *Ni-kshay* [30]. People with

TB receive daily ambulatory directly observed treatment by a health care provider, community volunteer or a family member.

Since 2017, India is implementing ACF in high-risk populations of all the districts ~~[31].~~[29]. Budgets (including incentives) are accounted for in the program implementation plans of the districts. All high-risk populations are to be identified (mapped) at the start of the year and added to *Ni-kshay* ~~[31]. Against each mapped population, ACF activity day-linked aggregate numbers~~[29]. High risk populations are broadly classified into clinically, socio-economically and geographically high risk (see Table 1). Against each mapped population, aggregate numbers linked to each ACF activity day are captured: date of ACF activity, number screened, number tested and number diagnosed (number of ACF-detected presumptive TB is not captured). ACF activity days are the days identified by the district in the year when ACF will be done, irrespective of the mode (campaign mode, in multiple rounds or on fixed days of a month) or scale.

The algorithm used for ACF under NTEP involves symptom-screen (among high-risk populations~~, see Table 1~~) followed by sputum microscopy or rapid molecular test (Xpert MTB/Rif~~®~~ or TrueNat~~®~~) among symptom-screen positive, also called as ACF-detected presumptive (pulmonary) TB. Symptom-screen positive is defined as the presence of cough or fever of two or more weeks or unexplained weight loss or chest pain or hemoptysis. Among clinically vulnerable, cough or fever of any duration is considered ~~[30,31]~~[28,29].

~~The national ACF guidance provides benchmarks for ACF scale and quality (Figure 1) but not for time taken and extent of use of rapid molecular diagnostic tests in the care cascade [30,31]. The 2017 ACF guidance recommended the use of a paper-based ACF tool in the field to collect individual level information of those screened, based on which aggregate~~

~~numbers are to be filled in *Ni-kshay* [31]. The ACF tool includes population level information regarding the address and the specific type of high-risk population and individual level information like name, age, gender, mobile number, symptom details, presumptive pulmonary TB (yes/no) and details of testing and TB diagnosis if found to be presumptive TB. The 2019 ACF guidance did not mention the use of this paper-based ACF tool in the field [30]. Most districts and states are implementing one ACF cycle per year with limited use of the paper-based ACF tool in the field (2021) [17,33].~~

The national ACF guidance provides benchmarks for ACF scale and quality (Figure 1) but not for time taken and extent of use of rapid molecular diagnostic tests in the care cascade [28,29]. The 2017 ACF guidance recommended the use of a paper-based ACF tool in the field to collect individual level information of those screened, based on which aggregate numbers are to be filled in *Ni-kshay* [29]. The ACF tool includes population level information regarding the address and the specific type of high-risk population and individual level information like name, age, gender, mobile number, symptom details, presumptive pulmonary TB (yes/no) and details of testing and TB diagnosis if found to be presumptive TB. The 2019 ACF guidance did not mention the use of this paper-based ACF tool in the field [28]. Most districts and states are implementing one ACF cycle per year with limited use of the paper-based ACF tool in the field [26,31].

Study population

In 30 randomly sampled NTEP districts (nine states) across India (see Supplementary Figure), we included all persons belonging to high-risk populations (not general population) who were screened between January and September 2023 and whose information were

collected in a paper-based ACF tool used in the field (mobile-based in three districts belonging to one state). Aggregate data directly entered in *Ni-kshay* without the use of ACF tool was not verifiable and therefore, not considered.

We selected nine states by stratified random sampling based on the routinely available composite TB score measuring NTEP performance and TB burden for every state (>80 score classified as high, 60 to 80 as medium and <60 as low performance). Three states were selected from each stratum by simple random sampling. From the nine states, 30 NTEP districts were selected based on probability proportionate to size sampling (based on TB notification). Three NTEP districts each were selected from six states (Tamil Nadu, Telangana, Rajasthan, Bihar, Odisha, Meghalaya), except Gujarat, Uttarakhand and Delhi, from which four districts were selected (see Supplementary Figure).

Variables, source of data and data collection

~~During the national/state/district level orientation and re-orientation meetings and in writing, it was clarified that individual level data available from the ACF tool used in field (see Supplementary Annex) will be considered as ACF data. The ACF tool used was similar to the one recommended in the 2017 ACF guidance [31], with the addition of the following questions: whether rapid molecular test used for TB diagnosis (yes/no), whether chest radiograph was used for screening (yes/no). Use of the ACF tool provided an opportunity for cross-checking whether ACF happened in mapped high-risk populations, verify a certain proportion of those in the care cascade and provide quality control-linked incentives. This provided a certain sense of reliability to the aggregate numbers generated for the ACF care cascade [17].~~

During the national/ state/ district level orientation and re-orientation meetings and in writing, it was clarified by the investigator team that individual level data available from the ACF tool used in field (see Supplementary Annex) will be considered as ACF data. The ACF tool used was similar to the one recommended in the 2017 ACF guidance [29], with the addition of two questions: whether chest radiograph was used for screening (yes/no) and whether rapid molecular test used for TB diagnosis (yes/no). Use of the ACF tool provided an opportunity for cross-checking whether ACF happened in mapped high-risk populations, verify a certain proportion of those in the care cascade and provide quality control-linked incentives [31]. This provided a certain sense of reliability to the aggregate numbers generated for the ACF care cascade.

ACF was implemented in routine settings by the health system. The district NTEP team provided overall guidance in planning, training, implementation (including mapping), and monitoring. One project research assistant per district facilitated the use of ACF tool. Within a district at the beginning of the year, in each sub-district level administrative unit (block), high risk populations were mapped (population ID, address, nearest landmark, high-risk population type and high-risk population numbers). Then this information was aggregated at district level to generate total high-risk population: ~~overall~~broad and specific high-risk populations type~~.~~ (Table 1). For each ACF activity day, the project research assistant captured information on whether ACF tool was used in the field and compiled the ACF tool at district level. If there was any missing field or data inconsistency, they got it corrected. Beyond this, they did not interfere with the implementation and data quality control mechanism ~~that was~~ put in place by the district NTEP team.

Data management and analysis

The project research assistants single-entered (with adequate data entry checks) the paper-based ACF tool data (except name and contact number) into a web application with locally stored server at ICMR-National Institute of Epidemiology (ICMR-NIE), Chennai (except three districts belonging to one state that directly used mobile-based version of the ACF tool in the field). Data ~~entered~~collection and entry were monitored centrally by the project management team from ICMR-~~National Institute of Epidemiology, Chennai (ICMR-~~NIE~~)~~.. Descriptive and analytical statistics were used as appropriate using EpiData Analysis software v 2.2.2.183 (EpiData Association, Odense, Denmark).

As we captured ACF data from January to September 2023 and the districts aimed to implement one ACF cycle in 2023, 75% of the mapped high-risk population (aggregate number) was considered as intended for screening (denominator) during the study period.

The two ACF scale indicators (target) were: i) percentage of population mapped as high-risk (≥11%) and ii) percentage of mapped population screened in the ACF cycle (≥90%). The four ACF quality indicators (target) were: i) percentage of screened identified as presumptive TB (≥5%) ii) percentage of presumptive TB tested (≥95%) iii) percentage of tested diagnosed as TB (test positivity ≥5%) and iv) percentage of diagnosed put on treatment (≥95%). ~~These were (see Figure 1) in line with national ACF guidance [30]. NNS (number screened divided by number diagnosed) cut-off (≤1538) for the algorithm summarized ACF quality indicators into one [17]. We calculated the six indicators and NNS, overall and stratified by high-risk populations. Due to multiple (panning across the three broad high-risk population types) specific high-risk population type ascribed to a single mapped population, it was not possible to provide the two ACF scale indicators by broad high-risk population types (Table 1),~~These were (see Figure 1) in line with national ACF guidance [28]. NNS (number screened divided by number diagnosed) cut-off (≤1538) for the algorithm summarized ACF quality indicators

into one [26]. We calculated the six indicators and NNS, overall and stratified by high-risk populations. It was not possible to provide the two ACF scale indicator estimates by broad high-risk population types. The reason being there were many instances where a mapped population could be classified into multiple specific high-risk population types and these specific high-risk population types could be from more than one broad high-risk population types (see Table 1).

A meaningful difference of at least 10% for proportion of presumptive tested and extent of use of rapid molecular diagnostic test, and at least 2% for other proportions was considered significant if it also achieved statistical significance ($p<0.05$ using chi squared test).

Ethics

This study was part of the third phase of India central TB division commissioned national level TB ACF evaluation project. The evaluation project was approved by ICMR-NIE's ethics committee (NIE/IHEC/202201-10 dated 09 Feb 2022, renewal on 25 Jan 2023). As this study involved routinely collected secondary data (anonymous), waiver of written informed consent was sought and approved by the ethics committee.

RESULTS

The total population of 30 districts was 57 211 032. A total of 10 449 555 (18.3%) were mapped as high-risk, 75% of which (n= 7 837 170) were considered as denominator for further analysis.

There were a total of 1467 ACF activity days. ACF tool was used in the field in 1031 (70.3%) days. During the 1031 ACF activity days considered in the analysis, of the 7 837 170 high-risk population, 581 633 (7.4%, 95% CI: 7.42. 7.44) were screened (an average of 565 people screened per district per ACF activity day). Of these, 11 357 (1.95%, 95% CI: 1.92, 1.99) were identified as presumptive TB. Among those with presumptive TB, the prevalence estimates of symptoms [duration of symptoms] were as follows: cough in 70.3% [median 9 days (interquartile range: 1,15)], fever in 28.7% [median 5 days (interquartile range: 2,7)], weight loss in 8%, ~~hemoptysis~~haemoptysis in 2.7% and chest pain in 9.2%. Of 11 357, sputum specimen was collected for 8541 (75.2%) and ~~a total of~~ 7527 (66.3%, 95% CI: 65.4, 67.2) were tested ~~and among tested~~, of which 117 (1.6%, 95% CI: 1.3, 1.9) were diagnosed with TB. All the ACF-detected TB were initiated on treatment. The NNS was 4971. The ACF scale and quality indicators are depicted in Figure 2 and Table 2.

Half of those screened were male, 11% were elderly and 60% resided in rural areas (see Table 2). The most common type of high-risk population (by broad categories) screened was geographically high-risk (41%). Ten percent of high-risk individuals were screened at facilities (see Table 2). Higher percentage of presumptive TB among screened was observed in elderly (5.2% versus 1.6% in ≤60 years) and clinically high-risk (4.1% versus 1.3% in geographically high-risk). The percentage of testing among presumptive TB was low across all sub-groups and ranged from 64% to 71%. Higher test positivity was observed in socioeconomically high-risk (2.9% versus 0.6% in geographically high-risk). NNS was >1538 for all subgroups. It was as high as 18 331 for geographically high-risk (see Table 2).

The distribution of specific high-risk populations that were screened is as follows (those with 2.5% or higher contribution have been mentioned): areas with high notifications (34.1%), slums (20.6%), occupational risk (9.5%), unorganized labour (8.6 %), difficult to reach areas

(6.9%), tribal areas (2.9%) and noncommunicable disease and/or its risk factors and/or COVID19 recovered (2.7%) ~~(data not shown)~~. The percentage screened among the mapped population was the highest for unorganized labour (75.3%) with an NNS of 2495. The NNS was below or around the cut-off in prisons (NNS 882), contact tracing (NNS 1583) and noncommunicable disease and/or its risk factors and/or COVID19 recovered (NNS 1288) (Figure 2).

Of 7527 presumptive tested, 1989 (26.4%, 95% CI: 25.4, 27.4) underwent rapid molecular diagnostic test. High-risk populations in rural areas (18.3% versus 42.9% in urban), population-based high-risk groups (22.7% versus 43.2% for facility-based), and geographically high-risk (5.4% versus 41.1% for socioeconomically) had a significantly lower use (Table 3). The median time taken in the various steps of the care cascade ranged from zero to one day (Supplementary Table 2).

Of the high-risk population screened, a total of 9321 (1.6%) underwent upfront chest radiograph as a screening test~~. Most~~ and most of these people (n=8854, 95%) were from two districts of a state.~~ ~~ Of these 9321, a total of 1087 (11.7%) had an abnormality in chest radiograph, of whom 679 (62.5%) did not have any symptoms.

DISCUSSION

This study on TB ACF care cascade (2023) from India is possibly the first ever nationally representative study using individual level data that ~~has~~ assessed the scale and quality of an ACF cycle. During the ACF cycle, less than 10% of high-risk population was screened along with NNS of ≈5000. Time taken in the care cascade was minimal and use of rapid molecular diagnostic tests was observed in less than one-third of those tested. ~~Overall, but~~But for the

percentage of population mapped as high-risk and percentage ACF-detected TB initiated on treatment, none of the other four scale and quality indicators were above the benchmark. The percentage of mapped high-risk population screened fared the worst and percentage of presumptive TB tested fared the best. ~~During the ACF cycle, less than 10% of high-risk population was screened along with NNS of ≈5000. Time taken in the care cascade was minimal and use of rapid molecular diagnostic tests was observed in less than one-third of those tested.~~

Strengths and limitations

~~A systematic review assessing the effect of ACF on TB epidemiology recommended that ACF projects should incorporate a well-designed, robust evaluation to contribute to the evidence base [8]. This is one such example. Similar to our 2021 analysis [17], we analyzed the ACF scale and quality indicators for an ACF cycle. We did not merely start our analysis from those screened. This provides us with a holistic understanding of yield, so that corrective actions can be taken. We mapped high-risk populations and prospectively documented ACF care cascade, making it more reliable than previous secondary retrospective aggregate data-based analysis [17,28].~~

A systematic review assessing the effect of ACF on TB epidemiology recommended that ACF projects should incorporate a well-designed, robust evaluation to contribute to the evidence base [9]. This is one such example. Similar to our 2021 analysis [26], we analysed the ACF scale and quality indicators for an ACF cycle starting from high-risk populations; we did not merely start our analysis from those screened. This provided us with a holistic understanding of low yield, so that corrective actions can be taken. We mapped high-risk

populations and prospectively documented ACF care cascade, making it more reliable than previous secondary retrospective aggregate data-based analysis [26,27].

There were three limitations. First, we captured the extent of use of ACF tool by ACF activity days (1031 of 1467 ACF activity days) and not based on what proportion of *Ni-kshay* reported ACF care cascade data were captured by the ACF tool. The reason was that most districts did not update their *Ni-kshay* ACF data for the study period till the completion of our analysis. Second, as we had project research assistants posted in the study districts, these findings could present a best-case scenario with the true picture being worse. Finally, this being analysis of routinely captured data, data collection errors cannot be ruled out. The research assistants tried to minimize this when they compiled the forms at district level.

Inference from our findings

~~The results of our study have significant implications in improving ACF in India. To begin with, let us compare our findings with 2021 [17]. First, in 2021, based on secondary aggregate *Ni-kshay* data, nearly nine percent (cut-off 10%) of the population was reported to be screened indicating acceptable scale (Supplementary Table 1) [17]. However, the limitation here was that we were not sure whether those screened were from high-risk population [17]. Our estimates from this study, which are more reliable, suggest that only 7% of mapped high-risk population were screened (cut-off 90%). Even if the ACF tool was used in all ACF days and if the mapped high-risk population was reduced to 11% (instead of 18.3% observed), the estimated scale of ACF will increase only to 17.5%, way lower than the target of 90%.~~

Second, in 2021, 1% of screened were tested (target 4.75%) [17]. Our findings in 2023 suggest that it is due to a combination of sub-optimal screening in the field (low percentage of presumptive among those screened) and losses between presumption, sputum collection and testing. Not capturing aggregate number of presumptive among screened in *Ni-kshay* could be the reason for identifying sub-optimal screening in the field during implementation monitoring.

Third, our findings estimate a low test positivity when compared to 2021 [17]. We speculate that due to non-capture of number of presumptive TB in *Ni-kshay*, percentage presumptive among screened and percentage tested among presumptive cannot be generated. Hence, all the focus in the field in 2021 might have been on test positivity. In the event of not using ACF tool in the field and the absence of mechanisms to cross-check ACF-detected TB (whether they are truly ACF-detected), it is difficult to cross-verify test positivity reported in 2021. In the past, performance of ACF was also being routinely assesed using two methods focused on ACF-detected TB [34]: i) crude proportion of TB cases detected by PCF compared to ACF for a defined geographical area and period and ii) additional number of TB cases detected in a geographic area (before-during). These methods of monitoring have many limitations and should be done away with [34].

To begin with, let us compare our findings with 2021 [26]. First, in 2021, based on secondary aggregate *Ni-kshay* data, nearly nine percent (cut-off 10%) of the population was reported to be screened indicating acceptable scale (Supplementary Table 1) [26]. However, the limitation here was that we were not sure whether those screened were from high-risk population [26]. Our estimates from this study, which are more reliable, suggest that only 7% of mapped high-risk population were screened (cut-off 90%). Even if the ACF tool was used in all ACF days and if the mapped high-risk population was reduced to 11% (instead of

18.3% observed), the estimated scale of ACF will increase only to 17.5%, way lower than the target of 90%.

Second, in 2021, 1% of screened were tested (target 4.75%) [26]. Our findings in 2023 that provide information on all ACF quality indicators suggest that it is due to a combination of sub-optimal screening in the field (low percentage of presumptive among those screened) and losses between presumption, sputum collection and testing. Not capturing aggregate number of presumptive among screened in *Ni-kshay* could be the reason for identifying sub-optimal screening in the field during implementation monitoring.

Third, our findings estimate a low test positivity when compared to 2021 [26]. We speculate that due to non-capture of number of presumptive TB in *Ni-kshay*, percentage presumptive among screened and percentage tested among presumptive cannot be generated. Hence, all the focus in the field in 2021 might have been on test positivity. In the event of not using ACF tool in the field and the absence of mechanisms to cross-check ACF-detected TB (whether they are truly ACF-detected), it is difficult to cross-verify test positivity reported in 2021. In the past, performance of ACF was also being routinely assessed using two methods that focused on ACF-detected TB [32]: i) crude proportion of TB cases detected by PCF compared to ACF for a defined geographical area and period and ii) additional number of TB cases detected in a geographic area (before-during). These methods of monitoring have many limitations and should be done away with [32].

Overall, low scale makes ACF ineffective and high NNS makes ACF inefficient with already constrained resources. The concern of low yield due to scale and quality being below the benchmark has been repeatedly highlighted in previous assessments of NTEP ~~[35]~~[33].

By specific high-risk groups, the elderly and prison inmates in our study were an exception where ≥5% screened were identified as presumptive TB. NSS was within or around 1538 cut-off for prisons, contact tracing, non-communicable diseases and people who have recovered from COVID19. A systematic review (2006-19) has reported an NNS of 35 among contacts screened in low- and middle-income countries [8]. Another systematic review from low- and middle-income countries (1999-2020) has revealed that yield of ACF is high in prison, contacts, HIV, drug users and poor households [9]. By broad high-risk groups, geographically high-risk were screened the maximum in our study along with the highest NNS. The high NNS was due to lower percentage of screened identified as presumptive TB, lower test positivity and less use of rapid molecular diagnostic tests.

When compared to routine case-finding within NTEP (most being PCF), the test positivity in ACF care cascade was lower than PCF (1.6% versus 4.5%) [14]. The extent of use of rapid molecular diagnostic tests among ACF- and PCF-detected presumptive TB was similar (26% versus 23%) [14].

Implications for India's NTEP

In addition to recommendations from the first and second (qualitative) phase of the project published elsewhere [26,31], the implications from this study from third phase are as follows.

First, to explore in depth the reasons for ACF scale and quality being below the benchmark, a systematic qualitative enquiry is warranted. This has already been completed under the second phase of the TB ACF evaluation project (February to August 2023). Among NTEP staff at various levels, inadequate training, a significant knowledge gap and know-do gap was observed [31]. Six tips for improving scale and quality of ACF were recommended [31].

By specific high-risk groups, the elderly and prison inmates in our study were an exception where ≥5% screened were identified as presumptive TB. NSS was within or around 1538 cut-off for prisons, contact tracing, non-communicable diseases and people who have recovered from COVID19. A systematic review (2006-19) has reported an NNS of 35 among contacts screened in low- and middle-income countries [7]. Another systematic review from low- and middle-income countries (1999-2020) has revealed that yield of ACF is high in prison, contacts, HIV, drug users and poor households [8]. By broad high-risk groups, geographically high-risk were screened the maximum in our study along with the highest NNS. The high NNS was due to lower percentage of screened identified as presumptive TB, lower test positivity and less use of rapid molecular diagnostic tests.

When compared to routine case-finding within NTEP (most being PCF), the test positivity in ACF care cascade was lower than PCF (1.6% versus 4.5%) [13]. The extent of use of rapid molecular diagnostic tests among ACF- and PCF-detected presumptive TB appears to be similar (26% versus 23%) [13].

Implications for India's NTEP

In addition to recommendations from the first and second (qualitative) phase of the project published elsewhere [17,33], the implications from this study from third phase are as follows.

Second,M more effective planning and resources are required if we are to cover 90% of the high-risk populations at least once a year. There is scope to screen a lot more than the current average of 565 screened per ACF activity day per district reported in our study.. In resource constrained settings, specific high-risk groups (prison, contacts) may be prioritized.

Continuous

Third, continuous monitoring of various ACF quality indicators and increased access to rapid molecular diagnostic tests are recommended with a focus on geographically high-risk. To monitor percentage presumptive among screened and percentage tested among presumptive, aggregate number of presumptive TB should be captured against each ACF activity in the ACF module of *Ni-kshay*.

~~Our findings provide evidence in favour of using an ACF tool for individual level data collection in the field (see Supplementary Annex) so that reliable aggregate numbers can be reported in *Ni-kshay*. These can be randomly cross-checked. Quality-control linked incentives should be for every household visited and every ‘sputum collection and transport’ (and not linked to TB case detection) [36,37]. To initiate release of incentives for a period, as recommended in phase two of the project, completed ACF tool should be submitted, with verification of a certain percentage of data (verifying all ACF-detected) and meeting a conservative test positivity or NNS cut-off [33]. This was successfully done in project *Axshya* (before 2017), where ACF was implemented by non-governmental organizations in India [36]. Decisions about the use of such approaches must be considered in the planning phase [37].~~

~~Finally~~Fourth, our findings provide evidence in favour of using an ACF tool for individual level data collection in the field (see Supplementary Annex) so that reliable aggregate numbers can be reported in *Ni-kshay*. These can be randomly cross-checked. Additionally, ACF tool may be of use to disburse quality-control linked incentives [31]. Quality-control linked incentives should be for every household visited and every ‘sputum collection and transport’ (and not linked to number of ACF-detected person with TB) [34,35]. To initiate release of incentives for household visit and sputum collection and transport for a period, as recommended in phase two of the project, completed ACF tool should be submitted, with

verification of a certain percentage of data (includes verifying all ACF-detected were detected as a result of ACF) and meeting a conservative test positivity or NNS cut-off [31]. This was successfully done in project *Axshya* (before 2017), where ACF was implemented by non-governmental organizations in India [34]. Decisions about the use of such approaches must be considered in the planning phase [35].

Finally, more resources and effective planning, use of ACF tool for individual level data collection in the field, continuous monitoring of various ACF quality indicators, increased access to rapid molecular diagnostic tests and addressing the knowledge / know-do gap are applicable not only for India but also other high TB burden countries that plan to scale up ACF and improve quality in routine program settings. This is applicable irrespective of the ACF algorithm used. These planning and implementation barriers in attaining scale and quality, if unaddressed, will impact the attainment of optimal yield even in the context of adding hand held radiography with computer assisted diagnostics and rapid molecular tests for screening high-risk populations (in addition to symptom screen) [6]. The TB officers at district and state/province level, the national TB programs and their partners (non-Governmental organizations, funding agencies) supporting ACF activities should keep these in mind while planning and implementing ACF. As correctly stated by MacPherson P et al, while *ACF cannot and should not be a substitute for equitable access to responsive, affordable, accessible primary care services for all, with careful planning and substantial investment, ACF for TB can be an impactful approach to accelerating progress towards ending TB in high burden countries* [36].

~~, to explore in depth the reasons for ACF scale and quality being below the benchmark, a systematic qualitative enquiry is warranted. This has already been completed under the second phase of the TB ACF evaluation project (February to August 2023). Among NTEP~~

~~staff at various levels, inadequate training, a significant knowledge gap and know-do gap was observed [33].~~

~~More effective planning and resources, use of ACF tool for individual level data collection in the field, continuous monitoring of various ACF quality indicators, increased access to rapid molecular diagnostic tests and addressing the knowledge / know-do gap are applicable not only for India but also other high TB burden countries that plan to scale up ACF and improve quality in routine program settings. This is applicable irrespective of the screening algorithm used. The TB officers at district and state/province level, the national TB programs and their partners (non-Governmental organizations, funding agencies) supporting ACF activities should keep these in mind while planning and implementing ACF.~~

CONCLUSION

~~Through~~In India, using prospective individual level data ~~collection~~of TB ACF among high-risk populations in 2023, this study provided nationally representative estimates of all ACF scale and quality indicators, time taken and use of rapid molecular diagnostic tests~~ for TB ACF in India.~~. This study confirmed the previous findings of one cycle per year and ACF quality being below the benchmark (2021) ~~[17]. Additionally, low scale of ACF was confirmed in our study. The findings of this study are more reliable and therefore supersede the findings of 2021 that were based on retrospective secondary aggregate data [17]. Individual data recording~~[26]. Additionally, low scale of ACF was confirmed in our study. Individual level data collection of those screened facilitates implementation monitoring of routinely reported aggregate data in *Ni-kshay* ACF module, resulting in reliable indicators. ~~The use of rapid molecular tests among ACF-detected presumptive TB was more or less~~

~~similar to PCF-detected presumptive TB. To implement at least one ACF cycle a year at scale and with good quality, more effective planning and resources (including access to rapid molecular diagnostic tests) are recommended.~~Therefore, the findings of this study are more reliable and supersede the findings of 2021 that were based on retrospective secondary aggregate data [26]. The use of rapid molecular tests among ACF-detected presumptive TB was similar to PCF-detected presumptive TB. To influence TB transmission, at least one ACF cycle a year should be implemented at scale and with good quality. More effective planning and resources including access to rapid molecular diagnostic tests are recommended.

Acknowledgements: The India central TB division commissioned, USAID/JSI-supported, ICMR-National Institute of Epidemiology (ICMR-NIE) led, national level TB ACF evaluation project was a collaboration among several key institutions: ICMR-National Institute of Epidemiology (ICMR-NIE) in Chennai, India, USAID India and JSI India, both based in New Delhi; The WHO Country Office for India, also located in New Delhi; and the Central TB Division, Ministry of Health and Family Welfare, Government of India, situated in New Delhi. The authors express sincere gratitude for the invaluable support and contributions from all state TB cells, district TB cells, and the WHO NTEP medical consultant network in India. Additionally, we acknowledge the significant contributions made by research assistants and interns from ICMR-NIE, Chennai, India. The research assistants facilitated data capture in the study districts, while the interns provided essential monitoring of project activities and vital data management support. We acknowledge the following research assistants (not included in the author list): Mr Chandan Kumar Mishra, Mr Deepak Kumar, Ms Santilahun Nongstein, Mr K Arul, Mr Gathala Subhash, Mr Debjyoti Chatterjee,

Mr M Rajasekar, Mr Pandiyaraja, Mr Bnajingsuk Nahain Kyndail, Mr Jerry Hujon, Ms Daisy Daimary, Ms Divya Gupta, Ms Krishna Vaghela and Ms Khusbu Ahmas. We acknowledge the following ICMR-NIE interns: Ms. Divya V, Mr. Sathish Kumar, Ms. Suhana Khatoon, Dr. Kavya Rao Bagati, Ms. Lathika R, Ms. Alifia PS, Dr. Aishwarya Dhumale, Dr. Shwetha R, Ms. Rebecca John, Ms. N Bhooma, Ms. B Suvetha, Mr. Mahesh Gomasa, Mr. Divakar G, Mr. Vishnu Bagavath, and Ms VK Damini. We acknowledge the support of KhushiBaby in supporting mobile-based ACF tool for use in the field (part of their Community Health Integrated Platform) in three districts of a state.

Contributors: HDS, GK, ANS, BV, VR, SKM, SI, DT, AC, SD, JC, KR, RR and MVM conceived and designed the study. HDS, SKP, PR, RS, RRV, PKH, AF, PS, BS, GunjanK, SKP, MAB, KVS, MS, AKD, DB, MLP, DYW, AK, PK, JSP, SushmitaD, AjitK, AjayK, SY, SC, SSM, AmitK, MP, SachinS, ST, PM, BB, GM, BM, AR, BKM, DNB, UCT and KUK supported in data collection and entry. RS, SKP and HDS developed the web application for data entry. HDS, SKP and PR did the project management and coordinated the operations. HDS and SKP managed the data. HDS and SKP did the data cleaning and analysis. HDS, SKP and PR prepared the first draft of the paper. All the authors were involved in data visualization. All authors contributed, reviewed and approved this article. HDS and SKP accessed and verified the data. HDS, SKP, RR and MVM had final responsibility for the decision to submit for publication.

Funding: This study was funded by USAID through John Snow International (JSI) under the TB Implementation Framework Agreement (TIFA) (TB commitment grants 0011-0549-1024 and 0011-0549-1025 to MVM). All authors had full access to all the data and had final responsibility for the decision to submit for publication.

Disclaimer: The contents of this study document are the authors' sole responsibility and do not necessarily reflect the views of Central TB Division (India), World Health Organization, ICMR-NIE, JSI, other organizations authors are affiliated to, USAID or the United States Government.

Competing interest: ~~None declared~~There are no undeclared conflicts of interest.

Data availability statement: All the data are fully available without restriction

Figshare: The link contains a collection of the dataset (EpiData REC file), the codebook/data dictionary for dataset (MS Excel) and the programme file for analysis of dataset (EpiData PGM file) pertaining to this study. https://doi.org/10.6084/m9.figshare.c.7048385.v1

Data are available under the terms of the Creative Commons Attribution 4.0 International License (CC BY 4.0)

Supplementary material: ACF tool for individual level data collection in the field has been shared as Supplementary Annex. There are two supplementary tables and one supplementary figure.

Open access. This is an open access article distributed in accordance with the Creative Commons Attribution 4.0 Unported (CC BY 4.0) license, which permits others to copy, redistribute, remix, transform and build upon this work for any purpose, provided the original work is properly cited, a link to the licence is given, and indication of whether changes were made. See: https://creativecommons.org/licenses/by/4.0/.

ORCID IDs

Hemant Deepak Shewade https://orcid.org/0000-0002-8242-1911

REFERENCES

~~1.~~1. World Health Organization (WHO). Global Tuberculosis Report 2016. Geneva, Switzerland; 2016. Available: http://apps.who.int/iris/bitstream/10665/250441/1/9789241565394-eng.pdf?ua=1

2. World Health Organization (WHO). Global Tuberculosis Report 2023. Geneva, Switzerland; 2023. Available: https://www.who.int/publications/i/item/9789240083851

3. World Health Organization (WHO). Systematic screening for active tuberculosis: an operational guide. Geneva, Switzerland, Switzerland; 2015. Available: http://apps.who.int/iris/bitstream/10665/181164/1/9789241549172_eng.pdf?ua=1

~~2~~4. Creswell J, Ottmani SE, Wei D, Sahu S, Lönnroth K, Uplekar MW. Programmatic approaches to screening for active tuberculosis. Int J Tuberc Lung Dis. 2013;17: 1248–1256. doi:10.5588/ijtld.13.0199

~~3~~5. Lönnroth K, Corbett E, Golub J, Godfrey-Faussett P, Uplekar M, Weil D, et al. Systematic screening for active tuberculosis: rationale, definitions and key considerations [State of the art series. Active case finding/screening. Number 1 in the series]. Int J Tuberc Lung Dis. 2013;17: 289–298. doi:10.5588/ijtld.12.0797

~~4. World Health Organization (WHO). Global Tuberculosis Report 2023. Geneva, Switzerland; 2023.~~

~~5~~6. World Health Organization. World Health Organization (WHO) consolidated guidelines on tuberculosis. Module 2: Screening-Systematic screening for tuberculosis disease. Geneva, Switzerland; 2021.

~~6~~7. Pramono JS, Ridwan A, Maria IL, Syam A, Russeng SS, Syamsuar, et al. Active Case Finding for Tuberculosis in Migrants: a Systematic Review. Med Arch (Sarajevo, Bosnia Herzegovina). 2024;78: 60–64. doi:10.5455/medarh.2024.78.60-64

~~7~~8. Velleca M, Malekinejad M, Miller C, Abascal Miguel L, Reeves H, Hopewell P, et al. The yield of tuberculosis contact investigation in low- and middle-income settings: a systematic review and meta-analysis. BMC Infect Dis. 2021;21: 1011. doi:10.1186/s12879-021-06609-3

~~8~~9. Bohlbro AS, Hvingelby VS, Rudolf F, Wejse C, Patsche CB. Active case-finding of tuberculosis in general populations and at-risk groups: a systematic review and meta-analysis. Eur Respir J. 2021;58: 2100090. doi:10.1183/13993003.00090-2021

~~9~~10. Haeusler IL, Torres-Ortiz A, Grandjean L. A systematic review of tuberculosis detection and prevention studies in prisons. Glob Public Health. 2022;17: 194–209. doi:10.1080/17441692.2020.1864753

Formatted: Normal (Web), Space Before: 0 pt, After: 0 pt, Widow/Orphan control, Adjust space between Latin and Asian text, Adjust space between Asian text and numbers

Formatted: Normal (Web), Space Before: 0 pt, After: 0 pt, Widow/Orphan control, Adjust space between Latin and Asian text, Adjust space between Asian text and numbers

~~10~~11. Gogichadze N, Sagrera A, Vicente JÁ, Millet J-P, López-Seguí F, Vilaplana C. Cost-effectiveness of active tuberculosis screening among high-risk populations in low tuberculosis incidence countries: a systematic review, 2008 to 2023. Euro Surveill. 2024;29: 2300614. doi:10.2807/1560-7917.ES.2024.29.12.2300614

~~11~~12. World Health Organization (WHO). Optimizing active case-finding for tuberculosis. Geneva, Switzerland; 2021.

~~12~~13. Burke RM, Nliwasa M, Feasey HRA, Chaisson LH, Golub JE, Naufal F, et al. Community-based active case-finding interventions for tuberculosis: a systematic review. Lancet Public Heal. 2021;6: e283–e299. doi:10.1016/S2468-2667(21)00033-5

~~13~~14. Central TB Division. Ministry of Health and Family Welfare. Government of India. India TB Report 2023. New Delhi, India; 2023.

~~14. Quraishi S, Quraishi H, Yadav H, Singh A, Fasih I, Vasquez NA, et al. Digital Storytelling and Community Engagement to Find Missing TB Cases in Rural Nuh, India. Trop Med Infect Dis 2022, Vol 7, Page 49. 2022;7: 49. doi:10.3390/TROPICALMED7030049~~

~~15. World Health Organization (WHO). Global Tuberculosis Report 2021. Geneva, Switzerland; 2021 Oct.~~

~~16. Central TB Division. Ministry of Health and Family Welfare. Government of India. India TB Report 2020. New Delhi, India; 2020.~~

~~17~~15. Garg T, Chaisson LH, Naufal F, Shapiro AE, Golub JE. A systematic review and meta-analysis of active case finding for tuberculosis in India. Lancet Reg Heal - Southeast Asia. 2022;7: 100076. doi:10.1016/j.lansea.2022.100076

16. Leo S, Rajaram M, Mohapatra MM, Chinnakali P, Joseph NM, Vemuri MB. Active Case Finding for Tuberculosis among Health Care Workers in a Teaching Hospital, Puducherry, India. Indian J Occup Environ Med. 2023;27: 42–48. doi:10.4103/ijoem.ijoem_342_21

17. Chawla S, Gupta V, Gour N, Grover K, Goel PK, Kaushal P, et al. Active case finding of tuberculosis among household contacts of newly diagnosed tuberculosis patients: A community-based study from southern Haryana. J Fam Med Prim care. 2020;9: 3701–3706. doi:10.4103/jfmpc.jfmpc_532_20

18. Narayan G, Vasudevan K, Rajagopal A, Gunasekaran K, Savla H, Suvvari TK. Active Screening for Pulmonary Tuberculosis Among Jail Inmates: A Mixed Method Study From Puducherry, South India. Cureus. 2023;15: e39749. doi:10.7759/cureus.39749

19. Shukla G, Sarin R, Vohra V, Khayyam KU. To study the impact of active case finding (ACF) among the TB patients detected in South Delhi. Indian J Tuberc. 2021;68: 80–84. doi:10.1016/j.ijtb.2020.08.011

Formatted: Normal (Web), Space Before: 0 pt, After: 0 pt, Widow/Orphan control, Adjust space between Latin and Asian text, Adjust space between Asian text and numbers

20. Shriraam V, Srihari R, Gayathri T, Murali L. Active case finding for Tuberculosis among migrant brick kiln workers in South India. Indian J Tuberc. 2020;67: 38–42. doi:10.1016/j.ijtb.2019.09.003

21. Vyas A, Creswell J, Codlin AJ, Stevens R, Rao VG, Kumar B, et al. Community-based active case-finding to reach the most vulnerable: tuberculosis in tribal areas of India. Int J Tuberc Lung Dis. 2019;23: 750–755. doi:10.5588/ijtld.18.0741

22. Dey A, Thekkur P, Ghosh A, Dasgupta T, Bandopadhyay S, Lahiri A, et al. ~~- Shewade HD, Kiruthika G, Ravichandran P, Iyer S, Chowdhury A, Kiran Pradeep S, et al. Quality of active case-finding for tuberculosis in India: a national level secondary data analysis. Glob Health Action. 2023;16: 2256129. doi:10.1080/16549716.2023.2256129~~

~~18. Leo S, Rajaram M, Mohapatra MM, Chinnakali P, Joseph NM, Vemuri MB. Active Case Finding for Tuberculosis among Health Care Workers in a Teaching Hospital, Puducherry, India. Indian J Occup Environ Med. 2023;27: 42–48. doi:10.4103/ijoem.ijoem_342_21~~

~~19. Chawla S, Gupta V, Gour N, Grover K, Goel PK, Kaushal P, et al. Active case finding of tuberculosis among household contacts of newly diagnosed tuberculosis patients: A community-based study from southern Haryana. J Fam Med Prim care. 2020;9: 3701–3706. doi:10.4103/jfmpc.jfmpc_532_20~~

~~20. Narayan G, Vasudevan K, Rajagopal A, Gunasekaran K, Savla H, Suvvari TK. Active Screening for Pulmonary Tuberculosis Among Jail Inmates: A Mixed Method Study From Puducherry, South India. Cureus. 2023;15: e39749. doi:10.7759/cureus.39749~~

~~21. Shukla G, Sarin R, Vohra V, Khayyam KU. To study the impact of active case finding (ACF) among the TB patients detected in South Delhi. Indian J Tuberc. 2021;68: 80–84. doi:10.1016/j.ijtb.2020.08.011~~

~~22. Shriraam V, Srihari R, Gayathri T, Murali L. Active case finding for Tuberculosis among migrant brick kiln workers in South India. Indian J Tuberc. 2020;67: 38–42. doi:10.1016/j.ijtb.2019.09.003~~

~~23. Vyas A, Creswell J, Codlin AJ, Stevens R, Rao VG, Kumar B, et al. Community-based active case-finding to reach the most vulnerable: tuberculosis in tribal areas of India. Int J Tuberc Lung Dis. 2019;23: 750–755. doi:10.5588/ijtld.18.0741~~

~~24. Dey A, Thekkur P, Ghosh A, Dasgupta T, Bandopadhyay S, Lahiri A, et al.~~ Active Case Finding for Tuberculosis through TOUCH Agents in Selected High TB Burden Wards of Kolkata, India: A Mixed Methods Study on Outcomes and Implementation Challenges. Trop Med Infect Dis. 2019;4: 134. doi:10.3390/tropicalmed4040134

~~25~~23. Vijayan S, Jondhale V, Pande T, Khan A, Brouwer M, Hegde A, et al. Implementing a chest X-ray artificial intelligence tool to enhance tuberculosis screening in India:

Lessons learned. PLOS Digit Heal. 2023;2: e0000404. doi:10.1371/journal.pdig.0000404

~~26~~24. Prasad BM, Satyanarayana S, Chadha SS, Das A, Thapa B, Mohanty S, et al. Experience of active tuberculosis case finding in nearly 5 million households in India. Public Heal Action. 2016;6: 15–18. doi:10.5588/pha.15.0035

~~27~~25. Prasad BM, Thapa B, Chadha SS, Das A, Babu ER, Mohanty S, et al. Status of Tuberculosis services in Indian Prisons. Int J Infect Dis. 2017;56: 117–121. doi:10.1016/j.ijid.2017.01.035

~~28~~26. Shewade HD, Kiruthika G, Ravichandran P, Iyer S, Chowdhury A, Kiran Pradeep S, et al. Quality of active case-finding for tuberculosis in India: a national level secondary data analysis. Glob Health Action. 2023;16: 2256129. doi:10.1080/16549716.2023.2256129

27. Nagaraja SB, Thekkur P, Satyanarayana S, Tharyan P, Sagili KD, Tonsing J, et al. Active case finding for tuberculosis in india: A syntheses of activities and outcomes reported by the national tuberculosis elimination programme. Tropical Medicine and Infectious Disease. 2021. p. 206. doi:10.3390/tropicalmed6040206

~~29. Garg T, Chaisson LH, Naufal F, Shapiro AE, Golub JE. A systematic review and meta-analysis of active case finding for tuberculosis in India. Lancet Reg Heal - Southeast Asia. 2022;7: 100076. doi:10.1016/j.lansea.2022.100076~~

~~30~~28. Central TB Division. Ministry of Health and Family Welfare. Govt of India. Active Case Finding. Guidance Document (Updated in August 2019 by State TB Cell Haryana for ACF campaign 2019). New Delhi, India; 2019.

~~31~~29. Central TB Division. Ministry of Health and Family Welfare. Government of India. Active Case Finding. Guidance Document. New Delhi, India; 2017.

~~32~~30. National TB Elimination Programme (NTEP) India. Nikshay Reports. 2020 [cited 8 May 2022]. Available: https://reports.nikshay.in/Reports/TBNotification

~~33~~31. Shewade HD, Ravichandran P, Pradeep SK, Kiruthika G, Shanmugasundaram D, Chadwick J, et al. Bridging the “know-do” gap to improve active case finding for tuberculosis in India: A qualitative exploration into national tuberculosis elimination program staffs’ perspectives. Goli A, editor. PLoS One. 2024;19: e0309750. doi:10.1371/journal.pone.0309750

~~34~~32. Satyanarayana S, Nagaraja SB. Measuring the performance of active TB case finding: programme managers need to be cautious. Public Heal Action. 2017;7: 178–178. doi:10.5588/pha.17.0023

~~35~~33. Central TB Division; Government of India. Report of the joint monitoring mission, revised national tuberculosis control programme, November 2019. New Delhi, India;

2019. Available: https://www.indiaspend.com/wp-content/uploads/2020/06/Report-of-JMM-RNTCP-Nov-2019_Mobile.pdf

~~36~~34. Shewade HD, Gupta V, Satyanarayana S, Pandey P, Bajpai UN, Tripathy JP, et al. Patient characteristics, health seeking and delays among new sputum smear positive TB patients identified through active case finding when compared to passive case finding in India. PLoS One. 2019;14: e0213345. doi:10.1371/journal.pone.0213345

~~37~~35. Singh AA, Creswell J, Bhatia V. Framework for planning and monitoring active TB case finding interventions to meet the global targets in the COVID-19 era and beyond: South-East Asia perspective. PLOS Glob public Heal. 2021;1: e0000073. doi:10.1371/journal.pgph.0000073

36. MacPherson P, Shanaube K, Phiri MD, Rickman HM, Horton KC, Feasey HRA, et al. Community-based active-case finding for tuberculosis: navigating a complex minefield. BMC Glob Public Heal. 2024;2. doi:10.1186/s44263-024-00042-9

FIGURE 1. Scale and quality indicators for an ACF cycle for TB, India, as per the national ACF guidance 2019 [30]

FIGURE 1. Scale and quality indicators for an ACF cycle for TB, India, as per the national ACF guidance 2019 [28]

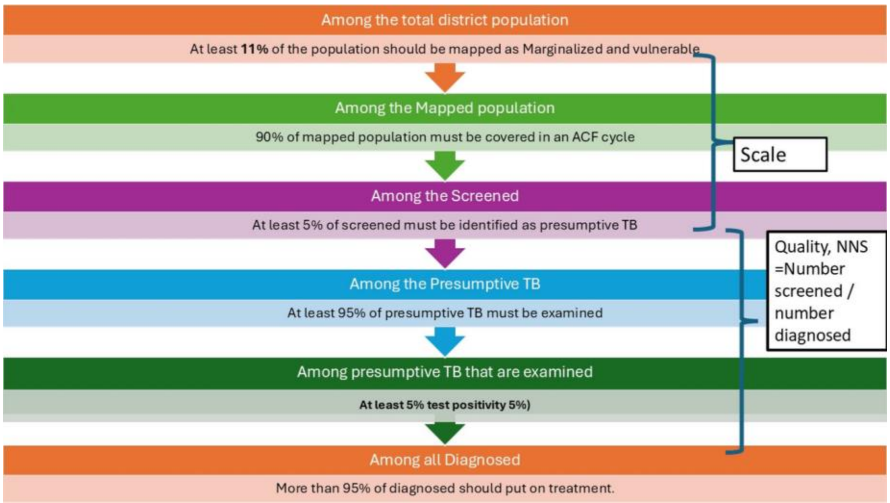

Abbreviations: ACF, active case-finding; TB, tuberculosis

~~Reprinted with permission from Shewade HD et al [17] under a CC BY license~~

Reprinted with permission from Shewade HD et al [26] under a CC BY license

FIGURE 2. Scale and quality indicators in the TB ACF cycle conducted from January to September 2023 in 30 randomly sampled NTEP districts of India, overall and stratified by specific high-risk groups [a]

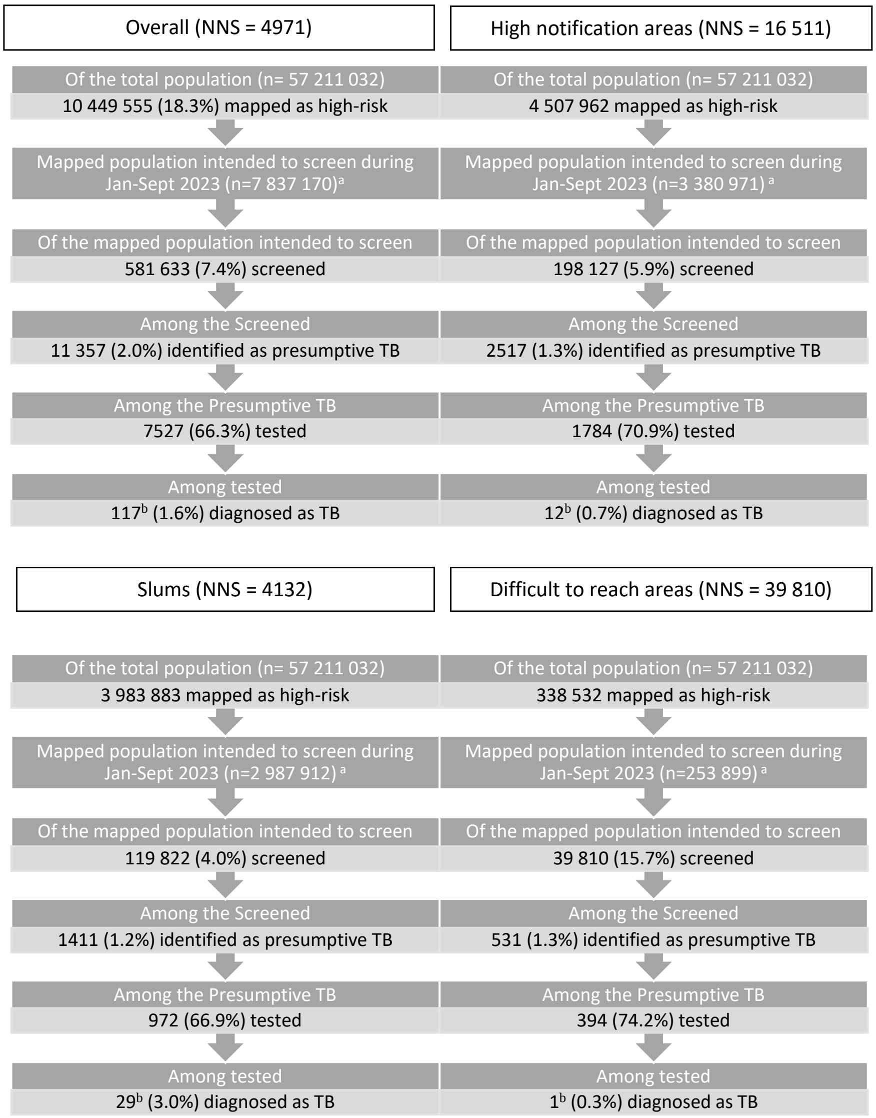

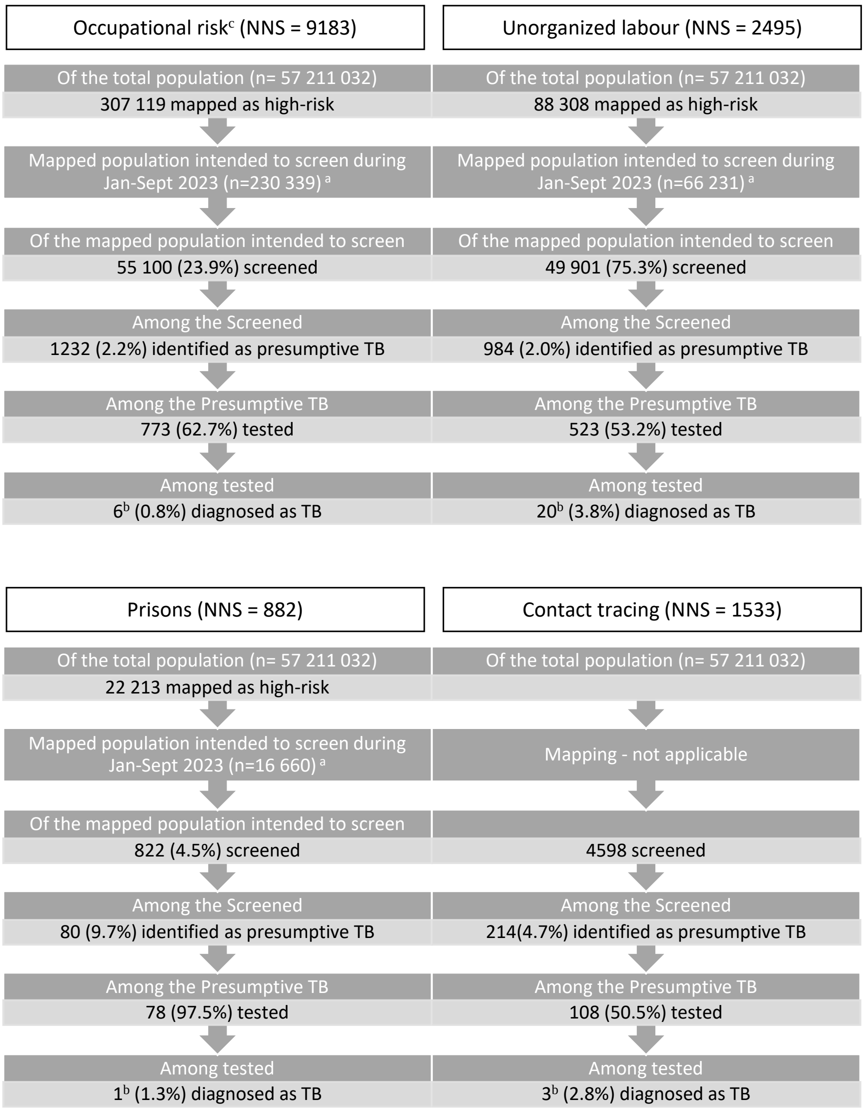

Abbreviations: ACF, active case-finding; TB, tuberculosis; NNS, number needed to screen (number screened divided by number diagnosed)

[a]75% of the mapped population was used as denominator as we captured ACF data for three-quarters of the year (one year=one ACF cycle); [a]Among screened, specific high-risk groups with 2.5% or higher contribution have been depicted. Data of prisons and contact screening has been included due to their importance; [b]all the ACF-detected TB were initiated on treatment; [c]occupation included construction site workers, cotton mill workers, mine workers, stone crushers, tea garden workers, weaving glass industrial workers, poultry workers, beedi workers

TABLE 1. Specific high-risk populations for active case-finding for TB classified[a] into three broad categories

**Clinical**	**Socioeconomic**	**Geographical (related to health access)**
• Contacts of people with TB<br>• Undernutrition<br>• HIV<br>• High risk groups for HIV identified by the program<br>• Noncommunicable diseases [Diabetes, hypertension, other cardiovascular diseases, chronic lung/liver/renal disease and their risk factors (alcohol, smoking, drug addiction, others)]<br>• People who have recovered from COVID19<br>• Immunocompromised (cancer), on palliative care<br>• Occupational risk with TB (construction site workers, weaving/glass industry workers, miners, cotton mill workers, stone crushers, beedi rolling, poultry workers, tea garden workers, health care workers)<br>• Elderly	• Prisons<br>• Old age homes, night shelters, home for destitute, orphanages, refugee camps, asylums<br>• Street children / homeless<br>• Unorganized labour<br>• Migrant population<br>• Slums<br>• Areas with high level of poverty / people living ~~the~~ below the poverty line<br>• Populations known to drink raw milk/uncooked meat<br>• Indigenous/ tribal population<br>• Sanitary workers	• Hard to reach areas<br>• Villages seeking care from traditional healers<br>• Areas with clustering of ~~cases~~TB<br>• Areas with low TB notification (than expected)

Abbreviations: TB, tuberculosis; HIV, human immunodeficiency virus; COVID19, corona virus disease 2019
[a]these specific high-risk groups may also be classified into facility-based and population-based, facility-based included orphanages, prison, refugee camps, homes for the destitute, tribal school homes, old age homes, night shelters, construction site workers, cotton mill workers, mine workers, stone crushers, tea garden workers, weaving, glass workers, industrial workers, poultry workers, beedi workers

TABLE 2. Quality indicators in the TB ACF cycle conducted from January to September 2023 in 30 randomly sampled NTEP districts of India, overall and stratified by gender, age, residence, and type of high-risk groups (broad categories)

**Category**		**Screened**		**Presumptive TB**		**Tested**		**Diagnosed[a]**		**NNS**
		**n**	**(%)[c]**	**n**	**(%)[r]**	**n**	**(%)[r]**	**n**	**(%)[r]**	
**Total**		**581 633**	**(100.0)**	**11 357**	**(2.0)**	**7527**	**(66.3)**	**117[a]**	**(1.6)**	**4971**
Gender	Male	293260	(50.4)	5798	(2.0)	3961	(68.3)	90	(2.3)	3258
	Female	284612	(48.9)	5526	(1.9)	3549	(64.4)	27	(0.8)	9208
	Other	48	(<0.1)	1	(2.1)	1	(100.0)	0	(0.0)	-
	Missing	3713	(0.6)	32	(0.9)	16	(50.0)	0	(0.0)	-
Elderly	Yes (>60y)	63645	(10.9)	3311	(5.2)[b]	2258	(68.2)	32	(1.4)	1989
	No	512 127	(88.0)	8007	(1.6)[~~b~~ref]	5239	(65.4)	85	(1.6)	6025
	Missing	5861	(1.0)	30	(0.5)	30	(100.0)	0	(0.0)	-
Residence	Rural	349300	(60.1)	7590	(2.2)	4994	(65.8)	57	(1.1)	6128
	Urban	232300	(39.9)	3766	(1.6)	2532	(67.2)	60	(2.4)	3872
	Missing	33	(<0.1)	1	(3.0)	1	(100.0)	0	(0.0)	-
Type of high-risk population										
	Socioeconomically	188 961	(32.5)	2893	(1.5)	1910	(66.0)	56	(2.9)[b]	3374
	Clinically	88 804	(15.3)	3621	(4.1)[b]	2504	(69.2)	26	(1.0)	3416
	Geographically	238 297	(41.0)	3049	(1.3)[~~b~~ref]	2178	(71.4)	13	(0.6)[~~b~~ref]	18331
	Not ~~classifiable~~classifiable[c]	63 011	(10.8)	1794	(2.8)	935	(52.1)	22	(2.4)	2864
Facility-based high-risk group										
	Yes	55 743	(9.6)	1399	(2.5)	915	(65.4)	8	(0.9)	6968
	No	501 619	(86.2)	8832	(1.8)	5976	(67.7)	93	(1.6)	5394
	Not classifiable	24 271	(4.2)	1126	(4.6)	636	(56.5)	16	(2.5)	1517

Abbreviations: TB, tuberculosis; ACF, active case-finding; NNS, number needed to screen

[a]all the ACF-detected TB were initiated on treatment

[b]public health wise significant difference (at least 10% for proportion of presumptive tested, at least 2% for other proportions) which was statistically significant ($p<0.05$ using chi squared test) was considered

Formatted: Font color: Black

[c]there were many instances where a mapped population could be classified into multiple specific high-risk population types and these specific high-risk population types could be from more than one broad high-risk population types

[c]column percentage

; [r]row percentage

; ®[ref]rReference

TABLE 3. Use of rapid molecular diagnostic test among ACF-detected presumptive TB who were tested in 30 randomly sampled NTEP districts of India, January to September 2023, overall and stratified by gender, age, residence and type of high-risk groups (broad categories)

**Category**		**Tested**	**Rapid molecular diagnostic test**	
		**N**	**n**	**(%)**[r]
**Total**		**7527**	**1989**	**(26.4)**
Gender	Male	3961	1150	(29.0)
	Female	3549	835	(23.5)
	Other	1	1	(100.0)
	Missing	16	3	(18.8)
Elderly	Yes (>60y)	2258	657	(29.1)
	No	5239	1329	(25.4)
	Missing	30	3	(10.0)
Residence	Rural	4994	902	(18.1) [a]
	Urban	2532	1087	(42.9)[ref]
	Missing	1	0	(0.0)
Type of high-risk population				
	Socioeconomically	1910	784	(41.0)[ref]
	Clinically	2504	783	(31.3)[a]
	Geographically	2178	114	(5.2) [a]
	Not ~~classifiable~~classifiable[b]	935	308	(32.9)
Facility-based high-risk group				
	Yes	915	395	(43.2)[ref]
	No	5976	1340	(22.4)[a]
	Not classifiable	636	254	(39.9)

Abbreviations: TB, tuberculosis; ACF, active case-finding; NNS, number needed to screen
[a]difference in proportion of at least 10% and statistically significant (p<0.05) using chi-squared test
[b]there were many instances where a mapped population could be classified into multiple specific high-risk population types and these specific high-risk population types could be from more than one broad high-risk population types
[r]row percentage
; [ref]~~r~~Reference

PGPH-D-25-01505
Active case-finding for TB in India: Assessment of scale and quality benchmarks, time taken and use of rapid molecular diagnostic tests

PLOS Global Public Health

Sub: Submission of revised manuscript

Dear Editor,
We would like to express our sincere gratitude for the constructive comments from you and the reviewers. We found them very helpful for improving the quality of our manuscript. We have made every effort to address each comment as carefully as we can.
All revised parts in the manuscript are in track change. Please find our point-by-point responses below. **The line numbers mentioned in our responses below are the line numbers from the "Revised Manuscript with Track Changes".** We have also taken this opportunity to make some editorial changes for better ease of reading. If there are any further clarifications, we would be very glad to address them,

We have also submitted a **"Manuscript.docx"** that is clean revised manuscript.

With regards,
Dr Hemant D Shewade

****
EDITOR COMMENTS

Additional Editor Comments (if provided):

COMMENTS

The manuscript is based on nationally representative individual data on Active Case Finding (ACF) strategy and its implementation. It is important. But it showed that despite massive effort, the ACF implementation is not detecting any significant number of TB cases. On the contrary, observations in their both cycles showed that the Passive Case Finding (PCF) is superior over the ACF. Considering this evidence and limited resources available for the NTEP, ideally ACF should be discouraged by the NTEP. Authors are advocating for the ACF and making some generic recommendations like monitoring, additional resource investments, proper implementation etc which is somehow paradoxical. The evidence clearly suggests that the NTEP should continue with passive case finding which is a more feasible and cost effective strategy. Authors may plan to undertake a major revision especially for discussion and conclusion sections in the light of the above observations. This is a fundamental point for revision.

RESPONSE

Thank you for the comment.

There are three ways in which ACF can be assessed.

One, looking at the cycle per say, the annual frequency, the scale and quality of each cycle, the delays and the extent of use chest radiograph as screening and rapid molecular tests as a screening/ diagnostic test.

Two, context specific studies to identify high-risk populations where ACF could be targeted and the ACF algorithm to be used

Third, assessing the benefit of ACF in the form of i) improvement in case detection in short term in areas where ACF is conducted followed by long term reduction in cases if ACF is conducted at sufficient frequency, scale and quality. ii) reduction in pre-treatment delays and costs due to TB diagnosis and treatment iii) reducing the societal / economic impact of TB

We intended to assess the scale and quality of ACF cycle in 2023 (using individual level prospective secondary data) and the previous ACF cycle in 2021 (using aggregate retrospective secondary data). The purpose of this specific exercise per say is not to assess the superiority of one strategy over the other and this is beyond the scope of this manuscript and the overall national level ACF evaluation study of which this manuscript is one of the outputs. In fact, ACF is recommended as an add-on strategy by WHO in high-risk populations to detect the missing TB. National level evaluations of ACF conducted by existing health workforce in routine health system setting like this help us understand where we are so that improvements can be made.

In addition to the 2023 and 2021 assessments of frequency, scale and quality (2021 assessment is published: https://pubmed.ncbi.nlm.nih.gov/37732993/), in 2023, we also completed a qualitative systematic enquiry among stakeholders to explore the why and how of these quantitative findings and have come up with 6 tips to improve ACF implementation. The findings are published in PLOS ONE https://dx.plos.org/10.1371/journal.pone.0309750. In 2023, we also completed the assessment as to whether ACF detects people with TB earlier when compared to PCF (manuscript under review).

Based on the scale and quality of ACF implemented in routine health system setting using existing workforce, the program can take a call on how best to put to use the resources into existing ACF initiatives.

In fact, there are modelling studies (cost benefit) that are available that suggest ACF has the benefits, especially if implemented well (at sufficient frequency, scale and quality) over long term (not just one or two years). The question is how to do in real world setting by the existing workforce in routine health system settings. This manuscript is in taking us in that direction.

I hope this is fine. We have made modifications in the manuscript in line with your comments that has significantly improved the manuscript.

Additionally, in the lines 467-77 of revised manuscript with track changes (reproduced below), we discuss the need for involvement of NGOs in ACF.

“Third, our findings provide evidence in favour of using an ACF tool for individual level data collection in the field (see Supplementary Annex) so that reliable aggregate numbers can be reported in Ni-kshay. These can be randomly cross-checked. Additionally, ACF tool may be of use to disburse quality-control linked incentives [31]. Quality-control linked incentives should be for every household visited and every ‘sputum collection and transport’ (and not linked to number of ACF-detected person with TB) [34,35]. To initiate release of incentives for household visit and sputum collection and transport for a period, as recommended in phase two of the project, completed ACF tool should be submitted, with verification of a certain percentage of data (includes verifying all ACF-detected were detected as a result of ACF) and meeting a conservative test positivity or NNS cut-off [31]. This was successfully done in project Axshya (before 2017), where ACF was implemented by non-governmental organizations in India [34]. Decisions about the use of such approaches must be considered in the planning phase [35].”

We have also added the following line 567-71 and important reference in the last paragraph under implications (discussion section)

As correctly stated by MacPherson P et al, while *ACF cannot and should not be a substitute for equitable access to responsive, affordable, accessible primary care services for all*, with *careful planning and substantial investment, ACF for TB can be an impactful approach to accelerating progress towards ending TB in high burden countries*.

Reference

MacPherson P, Shanaube K, Phiri MD, Rickman HM, Horton KC, Feasey HRA, et al. Community-based active-case finding for tuberculosis: navigating a complex minefield. BMC Glob Public Heal. 2024;2. doi:10.1186/s44263-024-00042-9

COMMENTS

Other comments are as below
1. Line 51: Specify the name of the program: NTEP

RESPONSE

Thank you. We have incorporated the suggestion.

COMMENTS

2. Line 52: Algorithm of what?

RESPONSE

We have clarified that is the TB ACF algorithm

COMMENTS

3. Lines 196-197: Please clarify the unit for these numbers

RESPONSE

The numbers in the parenthesis are nothing but the composite TB score measuring "NTEP performance and TB burden". To improve clarity, we have added the word score in the parenthesis. We hope this is fine.

COMMENTS

4. Lines 205-206: "During the national/state/district level orientation and re-orientation meetings and in writing, it was clarified that individual level data available from the ACF tool used in field (see Supplementary Annex) will be considered as ACF data. "Who claimed?

RESPONSE

It was clarified by the investigator team. We have made the necessary edits to clarify this. Please refer to lines 274-76 of revised manuscript with track changes.

COMMENTS

5. ACF diagnosed 1.6 % of cases and Passive case Finding (PCF) diagnosed 4.5% cases and use of rapid molecular tests were almost similar. It showed that PCF is superior than the ACF due to high yield and low resource requirement.

RESPONSE

As per the NTEP's ACF guidance, the expectation of test positivity among ACF detected presumptive TB is at least 5%. This is one of the ACF quality indicators. The reason for low test positivity could range from, not targeting high risk populations, not identifying presumptive TB correctly in the field, poor quality of sputum collected among ACF-detected presumptive TB in the field, delay in testing of sputum specimen and so on. The reason for suboptimal quality of ACF has explored in the qualitative phase of this national level TB ACF evaluation project. The manuscript from qualitative phase is published in PLOS ONE (https://dx.plos.org/10.1371/journal.pone.0309750). This has been discussed in lines 499-503, 545-555, of revised manuscript with track changes.

As mentioned before, the purpose of this national level ACF evaluation study is to determine, i) the frequency, scale and quality of TB ACF implemented by existing health workforce in routine NTEP settings including the use of rapid molecular diagnostic tests for ACF-detected presumptive TB ii) exploring the why and how of TB ACF scale and quality iii) whether ACF is resulting in early TB detection.

We intended to assess the scale and quality of ACF cycle in 2023 (using individual level prospective secondary data) and the previous ACF cycle in 2021 (using aggregate retrospective secondary data). The purpose of this specific exercise per say is not to assess the superiority of one strategy over the other and this is beyond the scope of this manuscript and the overall national level ACF evaluation project of which this manuscript is one of the outputs. In fact, ACF is recommended as an add-on strategy by WHO in high-risk populations to detect the missing TB. National level evaluations of ACF conducted by existing health workforce in routine health system setting like this help us understand where we are so that improvements can be made.

Based on our findings, the program can take a call on how best to put to use the resources into existing ACF initiatives. In fact, there are modelling studies (cost benefit) that are available that suggest ACF has the benefits, especially if implemented well (at sufficient frequency, scale and quality) over long term (not just one or two years). The question is how to do in real world setting by the existing workforce in routine health system settings. This manuscript is in taking us in that direction.

COMMENTS

6. The suggestions are generic and not based on findings of the study.

RESPONSE

We have revised the discussion in line with the comments (see lines 496-571 of revised manuscript with track changes). We have made every possible attempt to provide suggestions (in discussion section under the sub-section 'implications for India's NTEP') that are in line with the findings of the study.

COMMENTS

7. The manuscript requires significant editing to make it publishable.

RESPONSE

In line with the comments of the editor and the reviewers, we have gone through them carefully and to the best of our understanding, addressed the comments. We have also made use of this opportunity where two authors have gone through the manuscript again and made editorial changes to improve clarity and readability. We hope the revised manuscript is now satisfactory for publication.

****

REVIEWER COMMENTS

REVIEWER 1

COMMENT

Thank you for sharing the manuscript based on analysis of ACF data. It highlights an important aspect of significant strategy for case finding through analyzing secondary data. The manuscript is well drafted and covers important issues in implementing ACF strategies. However, there are few points to consider to further improve the manuscript.

RESPONSE

Thank you.

COMMENT

1. Introduction section
Strengths:
This study fills a vital evidence gap by providing nationally representative, prospectively collected, individual-level data on TB active case finding (ACF) a major upgrade from earlier reliance on local or retrospective aggregate data.

RESPONSE

Thank you.

COMMENT

The focus on scale and quality benchmarks and inclusion of rapid molecular diagnostic test usage makes it timely and highly relevant in the post-COVID TB control landscape.

RESPONSE

Thank you

COMMENT

Suggestion for improvement:
While the novelty is high, the research question is largely evaluative and programmatic rather than conceptual or hypothesis-driven. There is a scope for adding relevant modeling to make it theoretically robust.

RESPONSE

Thank you for your comment. This was a Central TB Division commissioned national level TB ACF evaluation project. The research questions and objectives were finalised after brainstorming between officers of Central TB Division and Scientists from ICMR-National Institute of Epidemiology.

The purpose of this national level ACF evaluation study was to determine, i) the frequency, scale and quality of TB ACF implemented by existing health workforce in routine NTEP settings including the use of rapid molecular diagnostic tests for ACF-detected presumptive TB ii) exploring the why and how of TB ACF scale and quality iii) whether ACF is resulting in early TB detection.

Addition of modelling is beyond the scope of this study. However, the information generated from this study from TB ACF done by existing workforce in routine health system settings can guide future modelling studies on TB ACF.

COMMENT

Introduction section need to be more tightened, the references need to be carefully sited

RESPONSE

Thank you for the comment. We have carefully gone though the introduction again, tightened it (from 745 to 659 words) and while doing so, ensured the references are correctly cited.

COMMENT

Make the objectives more self-explanatory.

RESPONSE

The ACF care cascade and the corresponding ACF scale and quality indicators (and their operational definitions) have already been clarified in the narrative text in introduction (before objectives) and in Figure 1. Hence, to avoid repetition and considering the multiple number of indicators, we have retained the objectives with some minor edits to improve clarity. We have not made major edits in the objectives. The authors feel that in the context of the preceding text in the introduction, the objectives are self-explanatory. For extent of use of rapid molecular tests, we have made edit starting that this is among ACF-detected people with presumptive TB. We hope this is fine.

COMMENT
2. Methodology
Strengths:
The design—a prospective cohort study using standardized tools (paper/mobile-based ACF tool)—ensures a high degree of data fidelity.

RESPONSE

Thank you.

COMMENT
The sampling strategy is robust, covering 30 districts across nine Indian states, stratified by performance tiers and selected by probability proportionate to size.

Suggestions:
There is limited explanation of how quality assurance was maintained across the data collection chain—especially given the scale and reliance on district-level staff.

RESPONSE

Thank you. We have clarified how quality assurance was maintained in lines 274-95 of revised manuscript with track changes.

COMMENT
ACF coverage was calculated using an assumption (75% of mapped population during Jan–Sept)—this operational workaround may skew the interpretation of scale.

RESPONSE

As we captured ACF data from January to September 2023 (75% of one year) and the districts aimed to implement one ACF cycle in 2023, 75% of the mapped high-risk population (aggregate number) was considered as intended for screening (denominator) during the study period. This has been clarified in lines 306-08 of revised manuscript with track changes.

What we have done, the authors believe is the right thing to do. It is not fair to use the complete (100%) high risk population as denominator when number screened is being taken from January – September period. We hope this is fine.

Hypothetically (for the sake of argument, this did not happen), if a district completed one ACF cycle for the year during January to September, then we should have taken 100% mapped high-risk population as the denominator. In this scenario, the scale (coverage mentioned by reviewer) would be even lower than currently estimated, with no major change in our inference or public health implications.

COMMENT

Though high-risk group has been explained in table but it needs to be explained in the text as well. Suggesting to add details about quality assurance.

RESPONSE

Thank you. We have explained the high-risk populations at first use under settings and cited Table 1 (see lines 211-12 of revised manuscript with track changes). We have clarified how quality assurance was maintained in lines 274-95 of revised manuscript with track changes.

COMMENT

3. Results and Interpretation
Strengths:
• The results are detailed, well-tabulated, and stratified across multiple high-risk groups—providing granular insights.
• The number needed to screen (NNS) calculation is a practical composite measure summarizing ACF efficiency.

RESPONSE

Thank you. We have used the word ACF quality (instead of efficiency) in line with National ACF guidance of India NTEP.

COMMENT
Suggestions:
• The paper rightly critiques the low performance but stops short of offering quantitative modeling of the potential impact of scale-up or quality improvement on TB notifications or prevalence.

RESPONSE

Thank you. This was a Central TB Division commissioned national level TB ACF evaluation project. The research questions were finalised after brainstorming between officers of Central TB Division and Scientists from ICMR-National Institute of Epidemiology.

The purpose of this national level ACF evaluation study was to determine, i) the frequency, scale and quality of TB ACF implemented by existing health workforce in routine NTEP settings including the use of rapid molecular diagnostic tests for ACF-detected presumptive TB ii) exploring the why and how of TB ACF scale and quality iii) whether ACF is resulting in early TB detection.

Addition of modelling is beyond the scope of this study. However, the information generated from this study from TB ACF done by existing workforce in routine health system settings can guide future modelling studies on TB ACF. Hence, we have limited the discussion to critiquing on the low performance and recommendation to improve it.

Regarding how improving scale and quality of ACF will have an effect on TB transmission, we have mentioned in the introduction section that conducting ACF at optimal frequency, high scale and quality (along with targeting high-risk populations and using highly sensitive screening and confirmatory tests) will result in high yield of ACF which will in turn have an impact on TB transmission (see lines of revised manuscript with track changes). We did not want to repeat this again in discussion. We hope this is fine.

COMMENT
• Adding more information about qualitative indicators can make it more robust if available.

RESPONSE

Thank you.

Pardon us, but we are not clear what the reviewer intends to mean here. If the reviewer intends to convey qualitative data collection and the themes emerging out of it, then following is our response.

In addition to the 2023 and 2021 assessments of frequency, scale and quality (2021 assessment is published: https://pubmed.ncbi.nlm.nih.gov/37732993/), in 2023, we also completed a qualitative systematic enquiry among stakeholders to explore the why and how of these quantitative findings and have come up with 6 tips to improve ACF implementation. The findings are published in PLOS ONE https://dx.plos.org/10.1371/journal.pone.0309750. The same has also been discussed and referenced in lines 499-503, 545-555 of revised manuscript with track changes.

COMMENT

4. Discussion and Policy Implications
Suggestions:
• Some conclusions repeat content from the results and could be more concise by adding more concluding statements.

RESPONSE

Thank you for the comment. We have revised this section by ensuring we do not repeat the content from results. We have ensured that the policy implications and conclusion section do not repeat the figures from results.

In some aspects of the discussion, as have to compare the findings of 2023 assessment (individual level data based, prospective) with 2021 (aggregate data based, retrospective), we had to repeat some of the result figures for comparison. We hope this is fine.

COMMENT
• Conclusion need to be re-written making it more conclusive and interpretative in terms of presenting the results.

RESPONSE

Thank you. We reviewed the conclusion section again. Considering the reviewer comment, we revised it to improve clarity, making it more conclusive and interpretative in terms of presenting the results (conclusion from revised manuscript copy and pasted below). We hope this is fine.

While writing the conclusion section, we have followed the principle of answering these three questions: what we did? What we found? What we recommend?

What we did? (copy pasting here from the revised manuscript)

In India, using prospective individual level data of TB ACF among high-risk populations in 2023, this study provided nationally representative estimates of all ACF scale and quality indicators, time taken and use of rapid molecular diagnostic tests.

What we found? (copy pasting here from the revised manuscript)

This study confirmed the previous findings of one cycle per year and ACF quality being below the benchmark (2021) [26]. Additionally, low scale of ACF was confirmed in our study. Individual level data collection of those screened facilitates implementation monitoring of routinely reported aggregate data in Ni-kshay ACF module, resulting in reliable indicators. Therefore, the findings of this study are more reliable and supersede the findings of 2021 that were based on retrospective secondary aggregate data [26]. The use of rapid molecular tests among ACF-detected presumptive TB was similar to PCF-detected presumptive TB.

What we recommend? (copy pasting here from the revised manuscript)

To influence TB transmission, at least one ACF cycle a year should be implemented at scale and with good quality. More effective planning and resources including access to rapid molecular diagnostic tests are recommended.

COMMENT
Overall comments

The author list is extensive (possibly over 60 authors)—which raises questions about authorship criteria, especially given the emphasis on field staff in acknowledgments.

RESPONSE

This is a national level evaluation of ACF that is implemented in routine health system settings involving 30 districts from 9 states. This requires involvement of all key stakeholders from NTEP at various levels from centre, state and district. This study would not have happened without their support. All those mentioned have fulfilled the ICMJE authorship criteria and their contribution have been mentioned in the contributors' section in the tail section of the manuscript (before references). Regarding field staff, the project field staff who fulfilled ICMJE authorship criteria have been included as co-authors. Those who did not fulfil the ICMJE criteria, have been acknowledged. We hope this is fine.

COMMENT

• The abstract has become quite descriptive and could be shortened by removing some redundant statistics to enhance clarity.

RESPONSE

We are not clear as to what the redundant statistics the reviewer is referring to. In the abstract, we have provided limited descriptive statistics only: two scale indicators, four quality indicators (NNS to summarize the four quality indicators into one indicator), extent of use of rapid molecular tests and the average time taken from screening to testing in days. These are all in line with the objectives. We hope this is fine.

COMMENT

Recommendations for Improvement

1. Add statistical modeling or sensitivity analyses to project the potential impact of meeting benchmark targets (e.g., if ACF achieved 90% coverage).

RESPONSE

Thank you. This was a Central TB Division commissioned national level TB ACF evaluation project. The research questions were finalised after brainstorming between officers of Central TB Division and Scientists from ICMR-National Institute of Epidemiology. Addition of modelling is beyond the scope of this study. However, the information generated from this study from TB ACF done by existing workforce in routine health system settings can guide future modelling studies on TB ACF. Hence, we have limited the discussion to critiquing on the low performance and recommendation to improve it.

Regarding how improving scale and quality of ACF will have an effect on TB transmission, we have already mentioned in the introduction section that conducting ACF at optimal frequency, high scale and quality (along with targeting high-risk populations and using highly sensitive screening and confirmatory tests) will result in high yield of ACF which will in turn have an impact on TB transmission (see lines 148-54 of revised manuscript with track changes). We did not want to repeat this again in discussion. We hope this is fine.

COMMENT
2. Include more discussion on implementation barriers, especially in low-resource districts—could be based on data from the second qualitative phase.

RESPONSE

Thank you for the comments.

In line with the reviewer comments, in lines 522-25 of revised manuscript with track changes, we have mentioned that in low-resource settings, districts may only target high-risk populations like contacts and prisons that have the highest burden of TB and therefore high NNS if ACF is implemented at optimal quality. In other words, instead of targeting 90% scale among all high-risk populations, low-resource districts may target prisons and contacts only.

Regarding implementation barriers that need to be addressed in line with data from second qualitative phase, we have highlighted these in lines 499-503, 545-555 of revised manuscript with track changes.

COMMENT

3. Reframe implications for global health programs—tie in learnings from other high-burden countries or WHO strategies.

RESPONSE

Thank you. We have reframed implications for global health programs of other high-burden countries. Please see lines 556-571 of revised manuscript with track changes.

We have also added the following important reference

MacPherson P, Shanaube K, Phiri MD, Rickman HM, Horton KC, Feasey HRA, et al. Community-based active-case finding for tuberculosis: navigating a complex minefield. BMC Glob Public Heal. 2024;2. doi:10.1186/s44263-024-00042-9

COMMENT

4. Clarify authorship rationale, particularly the roles of field-level personnel who contributed significantly but are listed outside the author list.

RESPONSE

This is a national level evaluation of ACF that is implemented in routine health system settings involving 30 districts from 9 states. This requires involvement of all key stakeholders from NTEP at various levels from centre, state and district. This study would not have happened without their support. All those mentioned have fulfilled the ICMJE authorship criteria and their contribution have been mentioned in the contributors' section in the tail section of the manuscript (before references). Regarding field staff, the project field staff who fulfilled ICMJE authorship criteria have been included as co-authors. Those who did not fulfil the ICMJE criteria, have been acknowledged. We hope this is fine.

****

REVIEWER 2

COMMENT

This is a well-timed study that provides detailed assessment of the ACF approach—highlighting specific areas for improvement—and proposes actionable, evidence-based suggestions to enhance its effectiveness.

RESPONSE

Thank you.

COMMENT

Overall comment: You may want to include how this recommendations can be taken forward within the TB programme. what strategy do you envision for integrating this recommendation into TB programme planning and execution?) Please refer to the specific in-text comments in the manuscript for details.

RESPONSE

Thank you. We have modified the manuscript in line with the in-text comments.

******* We received two manuscript file pdfs, both with same file name but different set of comments. Given the information provided to us were not able to differentiate which one was by reviewer 1 and which one by reviewer 2. *********

REVIEWER COMMENTS IN FIRST PDF – REVIEWER 1???

COMMENT

Line 88 - Make starting statement on a positive note

RESPONSE

Thank you. We have now begun the introduction on a positive note.

COMMENT

Line 105 - Mention the region & details of the studies

RESPONSE

In the preceding text, we have referred to four systematic reviews. This line the reviewer is referring to is an author inference of these studies mentioned in the systematic reviews "Most studies assessing ACF care cascade focus on quality (NNS) and number needed to test (test-positivity). There are limited studies that describe frequency, scale and yield."

COMMENT

Line 142-44 - Suggesting to make it more explanatory, add modeling perspective

RESPONSE

The reviewer is referring to the objectives.

The ACF care cascade and the corresponding ACF scale and quality indicators (and their operational definitions) have already been clarified in the narrative text in introduction (before objectives) and in Figure 1. Hence, to avoid repetition and considering the multiple number of indicators, we have not made major edits in the objectives. The authors feel that in the context of the preceding text in the introduction, the objectives are self-explanatory. For extent of use of rapid molecular tests, we have made edit starting that this is among ACF-detected people with presumptive TB. We hope this is fine.

Regarding modelling, this is beyond the scope of this study. This was a Central TB Division commissioned national level TB ACF evaluation project. The research questions were finalised after brainstorming between officers of Central TB Division and Scientists from ICMR-National Institute of Epidemiology. We have generated perhaps for the first time, national level estimates of scale and quality of ACF implemented by existing workforce in routine health system settings. Moving forward, this is vital information to guide ACF. Other high TB burden countries should also generate such estimates. The information generated from this study from TB ACF done by existing workforce in routine health system settings can guide future modelling studies on TB ACF.

COMMENT

Line 190 - Defined as?

RESPONSE

The reviewer is referring to high-risk population in study population. This term and its definition and the corresponding table 1 (that provides further details on high risk populations) have already been introduced under the settings section (see lines 211-13 of revised manuscript with track changes) that is before the study population section. We did not want to repeat the definition.

COMMENT

Line 248-50 - Pl write about how quality assurance was done

RESPONSE

Please see lines 274-95 of revised manuscript with track changes that has information on quality assurance.

COMMENT

Line 263-65 - "10 449 555 (18.3%) were mapped as high risk" - Including??

RESPONSE

This term 'high-risk population' and its definition and the corresponding table 1 (that provides further details on high risk populations) have already been introduced under the settings section (see lines 211-13 of revised manuscript with track changes) that is before the study population section.

The denominator and the care cascade among key specific high-risk population categories is provided in Figure 2. To ensure the flow of information does not break, we decided to not mention it at this stage in the results narrative. We hope this is fine.

COMMENT

Line 280 - Any particular high-risk category?

RESPONSE

The denominator and the care cascade among key specific high-risk population categories (high notification areas, geographically difficult to access) are provided in Figure 2. To ensure the flow of information does not break, we decided to not mention it at this stage in the results narrative. We hope this is fine.

COMMENT

Line 303-04 - Most of these were from two districts of a state – Incomplete statement

RESPONSE

Thank you. We have revised it as "Of the high-risk population screened, a total of 9321 (1.6%) underwent upfront chest radiograph as a screening test and most of these people (n=8854, 95%) were from two districts of a state."

COMMENT

Line 368 - NNS – need full form

RESPONSE

NNS was used first in the introduction section and the full form was provided at first use. We hope this is fine.

COMMENT

Line 377-78 - Is this a finding? What do we interpret from this?

RESPONSE

The reviewer is referring to the following line from the last para of the sub-section 'inference of our findings' from the discussion section

"When compared to routine case-finding within NTEP (most being PCF), the test positivity in ACF care cascade was lower than PCF (1.6% versus 4.5%) [13]. The extent of use of rapid molecular diagnostic tests among ACF- and PCF-detected presumptive TB appears to be similar (26% versus 23%)"

1.6% is a finding. It was already mentioned in results section. Here in discussion section, we are comparing this with 4.5% in PCF (not a study finding) and provided a reference for it. We are comparing test positivity among presumptive examination in ACF and PCF setting.

Again, 26% is a finding. It was already mentioned in results section. Here in discussion section, we are comparing this with 23% in PCF (not a study finding) and provided a reference for it. We are comparing extent of use of rapid molecular test in ACF and PCF setting. The extent of use of rapid molecular diagnostic test is almost similar in ACF and PCF setting. Considering the resources involved in ACF, rapid molecular diagnostic tests should be prioritized in ACF setting. The

implication of same on increase in resources and effective planning required has been discussed later in the discussion section.

We hope this clarifies.

COMMENT

Line 407-09 - This was not reported in the results

RESPONSE

In addition to the 2023 and 2021 assessments of frequency, scale and quality (2021 assessment is published: https://pubmed.ncbi.nlm.nih.gov/37732993/), in 2023, we also completed a qualitative systematic enquiry among stakeholders to explore the why and how of these quantitative findings and have come up with 6 tips to improve ACF implementation. The findings are published in PLOS ONE https://dx.plos.org/10.1371/journal.pone.0309750. Hence, these are not reported in the results of this manuscript. We have cited this qualitative study in discussion.

COMMENT

Line 424 – The findings of this study are more reliable

Seems like a biased statement. Suggesting to rephrase this

RESPONSE

The statement the reviewer is referring to is from conclusion

"The findings of this study are more reliable and therefore supersede the findings of 2021 that were based on retrospective secondary aggregate data"

The findings are more reliable than 2021. This is understood from the above statement. Why the findings on ACF scale and quality of this 2023 assessment (prospective, based on individual level data of those screened) are more reliable than 2021 assessment (retrospective, based on aggregate numbers) in introduction and discussion section. In fact, to generate more reliable estimates, was one of the justifications of this 2023 study. Please see lines 178-84, 274-83, 543-555 of revised manuscript with track changes.

COMMENT

Line 428-29 - This is more of a finding than the conclusion. Suggesting to add interpretive statement

RESPONSE

Thank you. The interpretive comment has been added in the next line. Please see lines 587-590 of revised manuscript with track changes.

****

REVIEWER COMMENTS IN SECOND PDF – REVIEWER 2???

COMMENT

Abstract summary - Can improvements in TB ACF scale and quality be achieved through planning and resource allocation alone, or do they also require deeper system-level strategies?

RESPONSE

Thank you for your comment. We completely agree with the reviewer and the same is reflected in the published findings from the qualitative phase (not part of this manuscript) of this national TB ACF evaluation project. Considering this is an abstract and has to be standalone, we did not include it in the abstract. We have discussed the findings of the qualitative phase in the discussion section of the main text. We hope this is fine.

COMMENT

Line 147 - Is this a cohort study? It uses individual level secondary data to understand scale and quality and not tracking individuals for any of the outcomes.

RESPONSE

The author group is clear that this is a cohort study. There are multiple outcomes in the care cascade, from getting identified as presumptive to getting tested and to getting diagnosed with TB. We present these results by various exposure (age, gender, rural/urban, high-risk population categories).

COMMENT

Line 184 - Did the guidance leave this out or refer to something else instead?

RESPONSE

The reviewer is referring to the statement "The 2019 ACF guidance did not mention the use of this paper-based ACF tool in the field" in methods section. The 2017 guidance had mentioned the use of an ACF tool, while 2019 guidance did not mention it. 2019 guidance also did not mention the use of something else instead of the ACF tool.

COMMENT

Line 205 - Any specific format ??? (guidelines, SOPs etc.)

RESPONSE

This was done through video calls and email communications. Same was re-iterated in person by the Research Assistant placed in the district TB cells (one research assistant per district TB cell).

COMMENT

Line 248-50 - Suggest simplifying this sentence for easy understanding.

RESPONSE

Thank you. We have simplified this. Please refer to lines 323-27 of revised manuscript with track changes, reproduced below:

“It was not possible to provide the two ACF scale indicator estimates by broad high-risk population types. The reason being there were many instances where a mapped population could be classified into multiple specific high-risk population types and these specific high-risk population types could be from more than one broad high-risk population types (see Table 1).”

COMMENT

Line 302 - Please clarify if it is- of 581633 high-risk population.

RESPONSE

Thank you. We have incorporated this.

COMMENT

If 9321 underwent upfront chest radio-graph, what happened to others?

RESPONSE

Others did not undergo upfront chest radio-graph as a screening test. They underwent the symptom-based screening only.

COMMENT

Line 429-31 - Specifying the planning and resource-related components will make this recommendation more actionable and credible.

RESPONSE

The planning and resource-related components have already been clarified in under “Implications for India’s NTEP” sub-section under discussion section. We did not want to repeat the specifics under conclusion. The conclusion has been drafted with an attempt to following this structure. What we did? What we found? What we recommend? While drafting we also ensured that we do not repeat the contents/ lines from the discussion section. We hope this is fine.

COMMENT

Line 473 - Is there any need for declaring competing interests of the authors who belong to Central TB Division, State TB cells and funding agencies?

RESPONSE

In the revised manuscript, under the competing interest statement we have clearly mentioned "There are no undeclared conflicts of interest."

In other words, we have declared in the title page that we have authors from USAID and John Snow International (these two names are also mentioned in the funding statement) and from Central TB Division.

In the Contributors statement, we have clearly mentioned what role each author (including) those from USAID and John Snow International and Central TB Division had to play. Hence, we have disclosed all the information in the article and we have no undeclared conflict of interest.

Beyond this we see no conflict of interest as there is no involvement of a “for-profit” funding agency, none of the authors received any compensation and there are no financial interest involved.

COMMENT

Table 1. Suggest specify which cases (TB??). Suggest adding "TB" notification. Change this to: below the poverty line

RESPONSE

Thank you for pointing this minor but important typo errors. We have made these corrections.

COMMENT

Table 2. Suggest including a brief explanation for Not classifiable

RESPONSE

Thank you. We have added a footnote in Table 2 and Table 3
