# Supplementary material for: Active case-finding for TB in India: Assessment of scale and quality benchmarks, time taken and use of rapid molecular diagnostic tests
Source: PLOS Glob Public Health. 2025 Oct 30;5(10):e0005103. doi: 10.1371/journal.pgph.0005103 (PMC12574901; doi:10.1371/journal.pgph.0005103)
Supplement: Attachment — Submitted filename: Response to comments_v2.docx [file pgph.0005103.s007.docx]

**PGPH-D-25-01505**
Active case-finding for TB in India: Assessment of scale and quality benchmarks, time taken and use of rapid molecular diagnostic tests

PLOS Global Public Health

Sub: Submission of revised manuscript

**Dear Editor,**

We would like to express our sincere gratitude for the constructive comments from you and the reviewers. We found them very helpful for improving the quality of our manuscript. We have made every effort to address each comment as carefully as we can.

All revised parts in the manuscript are in track change. Please find our point-by-point responses below. **The line numbers mentioned in our responses below are the line numbers from the “Revised Manuscript with Track Changes”.** We have also taken this opportunity to make some editorial changes for better ease of reading. If there are any further clarifications, we would be very glad to address them,

We have also submitted a **“Manuscript.docx”** that is clean revised manuscript.

With regards,

Dr Hemant D Shewade

*********

**EDITOR COMMENTS**

Additional Editor Comments (if provided):

COMMENTS

The manuscript is based on nationally representative individual data on Active Case Finding (ACF) strategy and its implementation. It is important. But it showed that despite massive effort, the ACF implementation is not detecting any significant number of TB cases. On the contrary, observations in their both cycles showed that the Passive Case Finding (PCF) is superior over the ACF. Considering this evidence and limited resources available for the NTEP, ideally ACF should be discouraged by the NTEP. Authors are advocating for the ACF and making some generic recommendations like monitoring, additional resource investments, proper implementation etc which is somehow paradoxical. The evidence clearly suggests that the NTEP should continue with passive case finding which is a more feasible and cost effective strategy. Authors may plan to undertake a major revision especially for discussion and conclusion sections in the light of the above observations. This is a fundamental point for revision.

RESPONSE

Thank you for the comment.

There are three ways in which ACF can be assessed.

One, looking at the cycle per say, the annual frequency, the scale and quality of each cycle, the delays and the extent of use chest radiograph as screening and rapid molecular tests as a screening/ diagnostic test.

Two, context specific studies to identify high-risk populations where ACF could be targeted and the ACF algorithm to be used

Third, assessing the benefit of ACF in the form of i) improvement in case detection in short term in areas where ACF is conducted followed by long term reduction in cases if ACF is conducted at sufficient frequency, scale and quality. ii) reduction in pre-treatment delays and costs due to TB diagnosis and treatment iii) reducing the societal / economic impact of TB

We intended to assess the scale and quality of ACF cycle in 2023 (using individual level prospective secondary data) and the previous ACF cycle in 2021 (using aggregate retrospective secondary data). The purpose of this specific exercise per say is not to assess the superiority of one strategy over the other and this is beyond the scope of this manuscript and the overall national level ACF evaluation study of which this manuscript is one of the outputs. In fact, ACF is recommended as an add-on strategy by WHO in high-risk populations to detect the missing TB. National level evaluations of ACF conducted by existing health workforce in routine health system setting like this help us understand where we are so that improvements can be made.

In addition to the 2023 and 2021 assessments of frequency, scale and quality (2021 assessment is published: https://pubmed.ncbi.nlm.nih.gov/37732993/), in 2023, we also completed a qualitative systematic enquiry among stakeholders to explore the why and how of these quantitative findings and have come up with 6 tips to improve ACF implementation. The findings are published in PLOS ONE <https://dx.plos.org/10.1371/journal.pone.0309750>. In 2023, we also completed the assessment as to whether ACF detects people with TB earlier when compared to PCF (manuscript under review).

Based on the scale and quality of ACF implemented in routine health system setting using existing workforce, the program can take a call on how best to put to use the resources into existing ACF initiatives.

In fact, there are modelling studies (cost benefit) that are available that suggest ACF has the benefits, especially if implemented well (at sufficient frequency, scale and quality) over long term (not just one or two years). The question is how to do in real world setting by the existing workforce in routine health system settings. This manuscript is in taking us in that direction.

I hope this is fine. We have made modifications in the manuscript in line with your comments that has significantly improved the manuscript.

Additionally, in the lines 467-77 of revised manuscript with track changes (reproduced below), we discuss the need for involvement of NGOs in ACF.

“Third, our findings provide evidence in favour of using an ACF tool for individual level data collection in the field (see Supplementary Annex) so that reliable aggregate numbers can be reported in Ni-kshay. These can be randomly cross-checked. Additionally, ACF tool may be of use to disburse quality-control linked incentives [31]. Quality-control linked incentives should be for every household visited and every ‘sputum collection and transport’ (and not linked to number of ACF-detected person with TB) [34,35]. To initiate release of incentives for household visit and sputum collection and transport for a period, as recommended in phase two of the project, completed ACF tool should be submitted, with verification of a certain percentage of data (includes verifying all ACF-detected were detected as a result of ACF) and meeting a conservative test positivity or NNS cut-off [31]. This was successfully done in project Axshya (before 2017), where ACF was implemented by non-governmental organizations in India [34]. Decisions about the use of such approaches must be considered in the planning phase [35].”

We have also added the following line 567-71 and important reference in the last paragraph under implications (discussion section)

As correctly stated by MacPherson P et al, while *ACF cannot and should not be a substitute for equitable access to responsive, affordable, accessible primary care services for all*, with *careful planning and substantial investment, ACF for TB can be an impactful approach to accelerating progress towards ending TB in high burden countries*.

Reference

MacPherson P, Shanaube K, Phiri MD, Rickman HM, Horton KC, Feasey HRA, et al. Community-based active-case finding for tuberculosis: navigating a complex minefield. BMC Glob Public Heal. 2024;2. doi:10.1186/s44263-024-00042-9

COMMENTS

Other comments are as below
1. Line 51: Specify the name of the program: NTEP

RESPONSE

Thank you. We have incorporated the suggestion.

COMMENTS

2. Line 52: Algorithm of what?

RESPONSE

We have clarified that is the TB ACF algorithm

COMMENTS

3. Lines 196-197: Please clarify the unit for these numbers

RESPONSE

The numbers in the parenthesis are nothing but the composite TB score measuring “NTEP performance and TB burden”. To improve clarity, we have added the word score in the parenthesis. We hope this is fine.

COMMENTS

4. Lines 205-206: "During the national/state/district level orientation and re-orientation meetings and in writing, it was clarified that individual level data available from the ACF tool used in field (see
Supplementary Annex) will be considered as ACF data. "Who claimed?

RESPONSE

It was clarified by the investigator team. We have made the necessary edits to clarify this. Please refer to lines 274-76 of revised manuscript with track changes.

COMMENTS

5. ACF diagnosed 1.6 % of cases and Passive case Finding (PCF) diagnosed 4.5% cases and use of rapid molecular tests were almost similar. It showed that PCF is superior than the ACF due to high yield and low resource requirement.

RESPONSE

As per the NTEP’s ACF guidance, the expectation of test positivity among ACF detected presumptive TB is at least 5%. This is one of the ACF quality indicators. The reason for low test positivity could range from, not targeting high risk populations, not identifying presumptive TB correctly in the field, poor quality of sputum collected among ACF-detected presumptive TB in the field, delay in testing of sputum specimen and so on. The reason for suboptimal quality of ACF has explored in the qualitative phase of this national level TB ACF evaluation project. The manuscript from qualitative phase is published in PLOS ONE (<https://dx.plos.org/10.1371/journal.pone.0309750>). This has been discussed in lines 499-503, 545-555, of revised manuscript with track changes.

As mentioned before, the purpose of this national level ACF evaluation study is to determine, i) the frequency, scale and quality of TB ACF implemented by existing health workforce in routine NTEP settings including the use of rapid molecular diagnostic tests for ACF-detected presumptive TB ii) exploring the why and how of TB ACF scale and quality iii) whether ACF is resulting in early TB detection.

We intended to assess the scale and quality of ACF cycle in 2023 (using individual level prospective secondary data) and the previous ACF cycle in 2021 (using aggregate retrospective secondary data). The purpose of this specific exercise per say is not to assess the superiority of one strategy over the other and this is beyond the scope of this manuscript and the overall national level ACF evaluation project of which this manuscript is one of the outputs. In fact, ACF is recommended as an add-on strategy by WHO in high-risk populations to detect the missing TB. National level evaluations of ACF conducted by existing health workforce in routine health system setting like this help us understand where we are so that improvements can be made.

Based on our findings, the program can take a call on how best to put to use the resources into existing ACF initiatives. In fact, there are modelling studies (cost benefit) that are available that suggest ACF has the benefits, especially if implemented well (at sufficient frequency, scale and quality) over long term (not just one or two years). The question is how to do in real world setting by the existing workforce in routine health system settings. This manuscript is in taking us in that direction.

COMMENTS

6. The suggestions are generic and not based on findings of the study.

RESPONSE

We have revised the discussion in line with the comments (see lines 496-571 of revised manuscript with track changes). We have made every possible attempt to provide suggestions (in discussion section under the sub-section ‘implications for India’s NTEP’) that are in line with the findings of the study.

COMMENTS

7. The manuscript requires significant editing to make it publishable.

RESPONSE

In line with the comments of the editor and the reviewers, we have gone through them carefully and to the best of our understanding, addressed the comments. We have also made use of this opportunity where two authors have gone through the manuscript again and made editorial changes to improve clarity and readability. We hope the revised manuscript is now satisfactory for publication.

*****************
**REVIEWER COMMENTS**

**REVIEWER 1**

COMMENT

Thank you for sharing the manuscript based on analysis of ACF data. It highlights an important aspect of significant strategy for case finding through analyzing secondary data. The manuscript is well drafted and covers important issues in implementing ACF strategies. However, there are few points to consider to further improve the manuscript.

RESPONSE

Thank you.

COMMENT

1. Introduction section
Strengths:
This study fills a vital evidence gap by providing nationally representative, prospectively collected, individual-level data on TB active case finding (ACF) a major upgrade from earlier reliance on local or retrospective aggregate data.

RESPONSE

Thank you.

COMMENT

The focus on scale and quality benchmarks and inclusion of rapid molecular diagnostic test usage makes it timely and highly relevant in the post-COVID TB control landscape.

RESPONSE

Thank you

COMMENT

Suggestion for improvement:
While the novelty is high, the research question is largely evaluative and programmatic rather than conceptual or hypothesis-driven. There is a scope for adding relevant modeling to make it theoretically robust.

RESPONSE

Thank you for your comment. This was a Central TB Division commissioned national level TB ACF evaluation project. The research questions and objectives were finalised after brainstorming between officers of Central TB Division and Scientists from ICMR-National Institute of Epidemiology.

The purpose of this national level ACF evaluation study was to determine, i) the frequency, scale and quality of TB ACF implemented by existing health workforce in routine NTEP settings including the use of rapid molecular diagnostic tests for ACF-detected presumptive TB ii) exploring the why and how of TB ACF scale and quality iii) whether ACF is resulting in early TB detection.

Addition of modelling is beyond the scope of this study. However, the information generated from this study from TB ACF done by existing workforce in routine health system settings can guide future modelling studies on TB ACF.

COMMENT

Introduction section need to be more tightened, the references need to be carefully sited

RESPONSE

Thank you for the comment. We have carefully gone though the introduction again, tightened it (from 745 to 659 words) and while doing so, ensured the references are correctly cited.

COMMENT

Make the objectives more self-explanatory.

RESPONSE

The ACF care cascade and the corresponding ACF scale and quality indicators (and their operational definitions) have already been clarified in the narrative text in introduction (before objectives) and in Figure 1. Hence, to avoid repetition and considering the multiple number of indicators, we have retained the objectives with some minor edits to improve clarity. We have not made major edits in the objectives. The authors feel that in the context of the preceding text in the introduction, the objectives are self-explanatory. For extent of use of rapid molecular tests, we have made edit starting that this is among ACF-detected people with presumptive TB. We hope this is fine.

COMMENT
2. Methodology
Strengths:
The design—a prospective cohort study using standardized tools (paper/mobile-based ACF tool)—ensures a high degree of data fidelity.

RESPONSE

Thank you.

COMMENT
The sampling strategy is robust, covering 30 districts across nine Indian states, stratified by performance tiers and selected by probability proportionate to size.
Suggestions:
There is limited explanation of how quality assurance was maintained across the data collection chain—especially given the scale and reliance on district-level staff.

RESPONSE

Thank you. We have clarified how quality assurance was maintained in lines 274-95 of revised manuscript with track changes.

COMMENT
ACF coverage was calculated using an assumption (75% of mapped population during Jan–Sept)—this operational workaround may skew the interpretation of scale.

RESPONSE

As we captured ACF data from January to September 2023 (75% of one year) and the districts aimed to implement one ACF cycle in 2023, 75% of the mapped high-risk population (aggregate number) was considered as intended for screening (denominator) during the study period. This has been clarified in lines 306-08 of revised manuscript with track changes.

What we have done, the authors believe is the right thing to do. It is not fair to use the complete (100%) high risk population as denominator when number screened is being taken from January – September period. We hope this is fine.

Hypothetically (for the sake of argument, this did not happen), if a district completed one ACF cycle for the year during January to September, then we should have taken 100% mapped high-risk population as the denominator. In this scenario, the scale (coverage mentioned by reviewer) would be even lower than currently estimated, with no major change in our inference or public health implications.

COMMENT

Though high-risk group has been explained in table but it needs to be explained in the text as well.
Suggesting to add details about quality assurance.

RESPONSE

Thank you. We have explained the high-risk populations at first use under settings and cited Table 1 (see lines 211-12 of revised manuscript with track changes). We have clarified how quality assurance was maintained in lines 274-95 of revised manuscript with track changes.

COMMENT

3. Results and Interpretation
Strengths:
• The results are detailed, well-tabulated, and stratified across multiple high-risk groups—providing granular insights.
• The number needed to screen (NNS) calculation is a practical composite measure summarizing ACF efficiency.

RESPONSE

Thank you. We have used the word ACF quality (instead of efficiency) in line with National ACF guidance of India NTEP.

COMMENT
Suggestions:
• The paper rightly critiques the low performance but stops short of offering quantitative modeling of the potential impact of scale-up or quality improvement on TB notifications or prevalence.

RESPONSE

Thank you. This was a Central TB Division commissioned national level TB ACF evaluation project. The research questions were finalised after brainstorming between officers of Central TB Division and Scientists from ICMR-National Institute of Epidemiology.

The purpose of this national level ACF evaluation study was to determine, i) the frequency, scale and quality of TB ACF implemented by existing health workforce in routine NTEP settings including the use of rapid molecular diagnostic tests for ACF-detected presumptive TB ii) exploring the why and how of TB ACF scale and quality iii) whether ACF is resulting in early TB detection.

Addition of modelling is beyond the scope of this study. However, the information generated from this study from TB ACF done by existing workforce in routine health system settings can guide future modelling studies on TB ACF. Hence, we have limited the discussion to critiquing on the low performance and recommendation to improve it.

Regarding how improving scale and quality of ACF will have an effect on TB transmission, we have mentioned in the introduction section that conducting ACF at optimal frequency, high scale and quality (along with targeting high-risk populations and using highly sensitive screening and confirmatory tests) will result in high yield of ACF which will in turn have an impact on TB transmission (see lines of revised manuscript with track changes). We did not want to repeat this again in discussion. We hope this is fine.

COMMENT
• Adding more information about qualitative indicators can make it more robust if available.

RESPONSE

Thank you.

Pardon us, but we are not clear what the reviewer intends to mean here. If the reviewer intends to convey qualitative data collection and the themes emerging out of it, then following is our response.

In addition to the 2023 and 2021 assessments of frequency, scale and quality (2021 assessment is published: https://pubmed.ncbi.nlm.nih.gov/37732993/), in 2023, we also completed a qualitative systematic enquiry among stakeholders to explore the why and how of these quantitative findings and have come up with 6 tips to improve ACF implementation. The findings are published in PLOS ONE <https://dx.plos.org/10.1371/journal.pone.0309750>. The same has also been discussed and referenced in lines 499-503, 545-555 of revised manuscript with track changes.

COMMENT

4. Discussion and Policy Implications
Suggestions:
• Some conclusions repeat content from the results and could be more concise by adding more concluding statements.

RESPONSE

Thank you for the comment. We have revised this section by ensuring we do not repeat the content from results. We have ensured that the policy implications and conclusion section do not repeat the figures from results.

In some aspects of the discussion, as have to compare the findings of 2023 assessment (individual level data based, prospective) with 2021 (aggregate data based, retrospective), we had to repeat some of the result figures for comparison. We hope this is fine.

COMMENT
• Conclusion need to be re-written making it more conclusive and interpretative in terms of presenting the results.

RESPONSE

Thank you. We reviewed the conclusion section again. Considering the reviewer comment, we revised it to improve clarity, making it more conclusive and interpretative in terms of presenting the results (conclusion from revised manuscript copy and pasted below). We hope this is fine.

While writing the conclusion section, we have followed the principle of answering these three questions: what we did? What we found? What we recommend?

What we did? (copy pasting here from the revised manuscript)

In India, using prospective individual level data of TB ACF among high-risk populations in 2023, this study provided nationally representative estimates of all ACF scale and quality indicators, time taken and use of rapid molecular diagnostic tests.

What we found? (copy pasting here from the revised manuscript)

This study confirmed the previous findings of one cycle per year and ACF quality being below the benchmark (2021) [26]. Additionally, low scale of ACF was confirmed in our study. Individual level data collection of those screened facilitates implementation monitoring of routinely reported aggregate data in Ni-kshay ACF module, resulting in reliable indicators. Therefore, the findings of this study are more reliable and supersede the findings of 2021 that were based on retrospective secondary aggregate data [26]. The use of rapid molecular tests among ACF-detected presumptive TB was similar to PCF-detected presumptive TB.

What we recommend? (copy pasting here from the revised manuscript)

To influence TB transmission, at least one ACF cycle a year should be implemented at scale and with good quality. More effective planning and resources including access to rapid molecular diagnostic tests are recommended.

COMMENT
Overall comments
The author list is extensive (possibly over 60 authors)—which raises questions about authorship criteria, especially given the emphasis on field staff in acknowledgments.

RESPONSE

This is a national level evaluation of ACF that is implemented in routine health system settings involving 30 districts from 9 states. This requires involvement of all key stakeholders from NTEP at various levels from centre, state and district. This study would not have happened without their support. All those mentioned have fulfilled the ICMJE authorship criteria and their contribution have been mentioned in the contributors’ section in the tail section of the manuscript (before references). Regarding field staff, the project field staff who fulfilled ICMJE authorship criteria have been included as co-authors. Those who did not fulfil the ICMJE criteria, have been acknowledged. We hope this is fine.

COMMENT
• The abstract has become quite descriptive and could be shortened by removing some redundant statistics to enhance clarity.

RESPONSE

We are not clear as to what the redundant statistics the reviewer is referring to. In the abstract, we have provided limited descriptive statistics only: two scale indicators, four quality indicators (NNS to summarize the four quality indicators into one indicator), extent of use of rapid molecular tests and the average time taken from screening to testing in days. These are all in line with the objectives. We hope this is fine.

COMMENT
Recommendations for Improvement
1. Add statistical modeling or sensitivity analyses to project the potential impact of meeting benchmark targets (e.g., if ACF achieved 90% coverage).

RESPONSE

Thank you. This was a Central TB Division commissioned national level TB ACF evaluation project. The research questions were finalised after brainstorming between officers of Central TB Division and Scientists from ICMR-National Institute of Epidemiology. Addition of modelling is beyond the scope of this study. However, the information generated from this study from TB ACF done by existing workforce in routine health system settings can guide future modelling studies on TB ACF. Hence, we have limited the discussion to critiquing on the low performance and recommendation to improve it.

Regarding how improving scale and quality of ACF will have an effect on TB transmission, we have already mentioned in the introduction section that conducting ACF at optimal frequency, high scale and quality (along with targeting high-risk populations and using highly sensitive screening and confirmatory tests) will result in high yield of ACF which will in turn have an impact on TB transmission (see lines 148-54 of revised manuscript with track changes). We did not want to repeat this again in discussion. We hope this is fine.

COMMENT
2. Include more discussion on implementation barriers, especially in low-resource districts—could be based on data from the second qualitative phase.

RESPONSE

Thank you for the comments.

In line with the reviewer comments, in lines 522-25 of revised manuscript with track changes, we have mentioned that in low-resource settings, districts may only target high-risk populations like contacts and prisons that have the highest burden of TB and therefore high NNS if ACF is implemented at optimal quality. In other words, instead of targeting 90% scale among all high-risk populations, low-resource districts may target prisons and contacts only.

Regarding implementation barriers that need to be addressed in line with data from second qualitative phase, we have highlighted these in lines 499-503, 545-555 of revised manuscript with track changes.

COMMENT

3. Reframe implications for global health programs—tie in learnings from other high-burden countries or WHO strategies.

RESPONSE

Thank you. We have reframed implications for global health programs of other high-burden countries. Please see lines 556-571 of revised manuscript with track changes.

We have also added the following important reference

MacPherson P, Shanaube K, Phiri MD, Rickman HM, Horton KC, Feasey HRA, et al. Community-based active-case finding for tuberculosis: navigating a complex minefield. BMC Glob Public Heal. 2024;2. doi:10.1186/s44263-024-00042-9

COMMENT

4. Clarify authorship rationale, particularly the roles of field-level personnel who contributed significantly but are listed outside the author list.

RESPONSE

This is a national level evaluation of ACF that is implemented in routine health system settings involving 30 districts from 9 states. This requires involvement of all key stakeholders from NTEP at various levels from centre, state and district. This study would not have happened without their support. All those mentioned have fulfilled the ICMJE authorship criteria and their contribution have been mentioned in the contributors’ section in the tail section of the manuscript (before references). Regarding field staff, the project field staff who fulfilled ICMJE authorship criteria have been included as co-authors. Those who did not fulfil the ICMJE criteria, have been acknowledged. We hope this is fine.

********************

**REVIEWER 2**

COMMENT

This is a well-timed study that provides detailed assessment of the ACF approach—highlighting specific areas for improvement—and proposes actionable, evidence-based suggestions to enhance its effectiveness.

RESPONSE

Thank you.

COMMENT

Overall comment: You may want to include how this recommendations can be taken forward within the TB programme. what strategy do you envision for integrating this recommendation into TB programme planning and execution?) Please refer to the specific in-text comments in the manuscript for details.

RESPONSE

Thank you. We have modified the manuscript in line with the in-text comments.

******* We received two manuscript file pdfs, both with same file name but different set of comments. Given the information provided to us were not able to differentiate which one was by reviewer 1 and which one by reviewer 2. *********

**REVIEWER COMMENTS IN FIRST PDF – REVIEWER 1???**

COMMENT

Line 88 - Make starting statement on a positive note

RESPONSE

Thank you. We have now begun the introduction on a positive note.

COMMENT

Line 105 - Mention the region & details of the studies

RESPONSE

In the preceding text, we have referred to four systematic reviews. This line the reviewer is referring to is an author inference of these studies mentioned in the systematic reviews “Most studies assessing ACF care cascade focus on quality (NNS) and number needed to test (test-positivity). There are limited studies that describe frequency, scale and yield.”

COMMENT

Line 142-44 - Suggesting to make it more explanatory, add modeling perspective

RESPONSE

The reviewer is referring to the objectives.

The ACF care cascade and the corresponding ACF scale and quality indicators (and their operational definitions) have already been clarified in the narrative text in introduction (before objectives) and in Figure 1. Hence, to avoid repetition and considering the multiple number of indicators, we have not made major edits in the objectives. The authors feel that in the context of the preceding text in the introduction, the objectives are self-explanatory. For extent of use of rapid molecular tests, we have made edit starting that this is among ACF-detected people with presumptive TB. We hope this is fine.

Regarding modelling, this is beyond the scope of this study. This was a Central TB Division commissioned national level TB ACF evaluation project. The research questions were finalised after brainstorming between officers of Central TB Division and Scientists from ICMR-National Institute of Epidemiology. We have generated perhaps for the first time, national level estimates of scale and quality of ACF implemented by existing workforce in routine health system settings. Moving forward, this is vital information to guide ACF. Other high TB burden countries should also generate such estimates. The information generated from this study from TB ACF done by existing workforce in routine health system settings can guide future modelling studies on TB ACF.

COMMENT

Line 190 - Defined as?

RESPONSE

The reviewer is referring to high-risk population in study population. This term and its definition and the corresponding table 1 (that provides further details on high risk populations) have already been introduced under the settings section (see lines 211-13 of revised manuscript with track changes) that is before the study population section. We did not want to repeat the definition.

COMMENT

Line 248-50 - Pl write about how quality assurance was done

RESPONSE

Please see lines 274-95 of revised manuscript with track changes that has information on quality assurance.

COMMENT

Line 263-65 - “10 449 555 (18.3%) were mapped as high risk” - Including??

RESPONSE

This term ‘high-risk population’ and its definition and the corresponding table 1 (that provides further details on high risk populations) have already been introduced under the settings section (see lines 211-13 of revised manuscript with track changes) that is before the study population section.

The denominator and the care cascade among key specific high-risk population categories is provided in Figure 2. To ensure the flow of information does not break, we decided to not mention it at this stage in the results narrative. We hope this is fine.

COMMENT

Line 280 - Any particular high-risk category?

RESPONSE

The denominator and the care cascade among key specific high-risk population categories (high notification areas, geographically difficult to access) are provided in Figure 2. To ensure the flow of information does not break, we decided to not mention it at this stage in the results narrative. We hope this is fine.

COMMENT

Line 303-04 - Most of these were from two districts of a state – Incomplete statement

RESPONSE

Thank you. We have revised it as “Of the high-risk population screened, a total of 9321 (1.6%) underwent upfront chest radiograph as a screening test and most of these people (n=8854, 95%) were from two districts of a state.”

COMMENT

Line 368 - NNS – need full form

RESPONSE

NNS was used first in the introduction section and the full form was provided at first use. We hope this is fine.

COMMENT

Line 377-78 - Is this a finding? What do we interpret from this?

RESPONSE

The reviewer is referring to the following line from the last para of the sub-section ‘inference of our findings’ from the discussion section

“When compared to routine case-finding within NTEP (most being PCF), the test positivity in ACF care cascade was lower than PCF (1.6% versus 4.5%) [13]. The extent of use of rapid molecular diagnostic tests among ACF- and PCF-detected presumptive TB appears to be similar (26% versus 23%)”

1.6% is a finding. It was already mentioned in results section. Here in discussion section, we are comparing this with 4.5% in PCF (not a study finding) and provided a reference for it. We are comparing test positivity among presumptive examination in ACF and PCF setting.

Again, 26% is a finding. It was already mentioned in results section. Here in discussion section, we are comparing this with 23% in PCF (not a study finding) and provided a reference for it. We are comparing extent of use of rapid molecular test in ACF and PCF setting. The extent of use of rapid molecular diagnostic test is almost similar in ACF and PCF setting. Considering the resources involved in ACF, rapid molecular diagnostic tests should be prioritized in ACF setting. The implication of same on increase in resources and effective planning required has been discussed later in the discussion section.

We hope this clarifies.

COMMENT

Line 407-09 - This was not reported in the results

RESPONSE

In addition to the 2023 and 2021 assessments of frequency, scale and quality (2021 assessment is published: https://pubmed.ncbi.nlm.nih.gov/37732993/), in 2023, we also completed a qualitative systematic enquiry among stakeholders to explore the why and how of these quantitative findings and have come up with 6 tips to improve ACF implementation. The findings are published in PLOS ONE <https://dx.plos.org/10.1371/journal.pone.0309750>. Hence, these are not reported in the results of this manuscript. We have cited this qualitative study in discussion.

COMMENT

Line 424 – The findings of this study are more reliable

Seems like a biased statement. Suggesting to rephrase this

RESPONSE

The statement the reviewer is referring to is from conclusion

“The findings of this study are more reliable and therefore supersede the findings of 2021 that were based on retrospective secondary aggregate data”

The findings are more reliable than 2021. This is understood from the above statement. Why the findings on ACF scale and quality of this 2023 assessment (prospective, based on individual level data of those screened) are more reliable than 2021 assessment (retrospective, based on aggregate numbers) in introduction and discussion section. In fact, to generate more reliable estimates, was one of the justifications of this 2023 study. Please see lines 178-84, 274-83, 543-555 of revised manuscript with track changes.

COMMENT

Line 428-29 - This is more of a finding than the conclusion. Suggesting to add interpretive statement

RESPONSE

Thank you. The interpretive comment has been added in the next line. Please see lines 587-590 of revised manuscript with track changes.

**********

**REVIEWER COMMENTS IN SECOND PDF – REVIEWER 2???**

COMMENT

Abstract summary - Can improvements in TB ACF scale and quality be achieved through planning and resource allocation alone, or do they also require deeper system-level strategies?

RESPONSE

Thank you for your comment. We completely agree with the reviewer and the same is reflected in the published findings from the qualitative phase (not part of this manuscript) of this national TB ACF evaluation project. Considering this is an abstract and has to be standalone, we did not include it in the abstract. We have discussed the findings of the qualitative phase in the discussion section of the main text. We hope this is fine.

COMMENT

Line 147 - Is this a cohort study? It uses individual level secondary data to understand scale and quality and not tracking individuals for any of the outcomes.

RESPONSE

The author group is clear that this is a cohort study. There are multiple outcomes in the care cascade, from getting identified as presumptive to getting tested and to getting diagnosed with TB. We present these results by various exposure (age, gender, rural/urban, high-risk population categories).

COMMENT

Line 184 - Did the guidance leave this out or refer to something else instead?

RESPONSE

The reviewer is referring to the statement “The 2019 ACF guidance did not mention the use of this paper-based ACF tool in the field” in methods section. The 2017 guidance had mentioned the use of an ACF tool, while 2019 guidance did not mention it. 2019 guidance also did not mention the use of something else instead of the ACF tool.

COMMENT

Line 205 - Any specific format ??? (guidelines, SOPs etc.)

RESPONSE

This was done through video calls and email communications. Same was re-iterated in person by the Research Assistant placed in the district TB cells (one research assistant per district TB cell).

COMMENT

Line 248-50 - Suggest simplifying this sentence for easy understanding.

RESPONSE

Thank you. We have simplified this. Please refer to lines 323-27 of revised manuscript with track changes, reproduced below:

“It was not possible to provide the two ACF scale indicator estimates by broad high-risk population types. The reason being there were many instances where a mapped population could be classified into multiple specific high-risk population types and these specific high-risk population types could be from more than one broad high-risk population types (see Table 1).”

COMMENT

Line 302 - Please clarify if it is- of 581633 high-risk population.

RESPONSE

Thank you. We have incorporated this.

COMMENT

If 9321 underwent upfront chest radio-graph, what happened to others?

RESPONSE

Others did not undergo upfront chest radio-graph as a screening test. They underwent the symptom-based screening only.

COMMENT

Line 429-31 - Specifying the planning and resource-related components will make this recommendation more actionable and credible.

RESPONSE

The planning and resource-related components have already been clarified in under “Implications for India’s NTEP” sub-section under discussion section. We did not want to repeat the specifics under conclusion. The conclusion has been drafted with an attempt to following this structure. What we did? What we found? What we recommend? While drafting we also ensured that we do not repeat the contents/ lines from the discussion section. We hope this is fine.

COMMENT

Line 473 - Is there any need for declaring competing interests of the authors who belong to Central TB Division, State TB cells and funding agencies?

RESPONSE

In the revised manuscript, under the competing interest statement we have clearly mentioned "There are no undeclared conflicts of interest."

In other words, we have declared in the title page that we have authors from USAID and John Snow International (these two names are also mentioned in the funding statement) and from Central TB Division.

In the Contributors statement, we have clearly mentioned what role each author (including) those from USAID and John Snow International and Central TB Division had to play. Hence, we have disclosed all the information in the article and we have no undeclared conflict of interest.

Beyond this we see no conflict of interest as there is no involvement of a “for-profit” funding agency, none of the authors received any compensation and there are no financial interest involved.

COMMENT

Table 1. Suggest specify which cases (TB??). Suggest adding "TB" notification. Change this to: below the poverty line

RESPONSE

Thank you for pointing this minor but important typo errors. We have made these corrections.

COMMENT

Table 2. Suggest including a brief explanation for Not classifiable

RESPONSE

Thank you. We have added a footnote in Table 2 and Table 3
