# Supplementary material for: Active case-finding for TB in India: Assessment of scale and quality benchmarks, time taken and use of rapid molecular diagnostic tests
Source: PLOS Glob Public Health. 2025 Oct 30;5(10):e0005103. doi: 10.1371/journal.pgph.0005103 (PMC12574901; doi:10.1371/journal.pgph.0005103)
Supplement: Attachment — Submitted filename: Response to comments.docx [file pgph.0005103.s009.docx]

**PGPH-D-25-01505**
Active case-finding for TB in India: Assessment of scale and quality benchmarks, time taken and use of rapid molecular diagnostic tests

PLOS Global Public Health

Sub: Submission of revised manuscript (R2)

**Dear Editor,**

We would like to express our sincere gratitude for the constructive comments from you and the reviewers. We found them very helpful for improving the quality of our manuscript. We have made every effort to address each comment as carefully as we can.

Please find our point-by-point responses below. We have also taken this opportunity to make some editorial changes for better ease of reading. If there are any further clarifications, we would be very glad to address them,

We have submitted a **“Manuscript.docx”** that is clean revised manuscript and a **“Revised manuscript with track changes.docx”**

With regards,

Dr Hemant D Shewade

*********

**EDITOR COMMENTS**

Additional Editor Comments (if provided):

COMMENTS

All comments are addressed.

RESPONSE

Thank you.

In line with reviewer 1 comments, some edits have been made in first para of introduction to improve clarity. As a result of these edits, we had to delete two references. the total references have now reduced from 36 to 34. As a result of this deletion of two references, all other references were reorganized by Mendeley in the Manuscript.docx (revised clean manuscript).

We generated the revised manuscript with track changes using the compare function in MS word. By looking at the revised manuscript with track changes it seems that major changes have been made. Due to changes in in text citations after the first para of introduction, many paragraphs have been highlighted in track changes. If one ignores this, in fact, the edits are minor in line with the reviewer comments. The specific edits have been described point by point in response to reviewer comments, quoting the lines in Manuscript.docx (revised clean manuscript).

*****************
**REVIEWER COMMENTS**

**REVIEWER 1**

COMMENT

Revise "Introduction section" especially the first paragraph which has a few unclear statements. The discussion section needs to be more structured.

RESPONSE

Thank you.

We have made some edits in first paragraph of introduction to improve clarity. Please see lines 89-97 in Manuscript.docx (revised clean manuscript). As a result of these edits, the total references have now reduced from 36 to 34.

We have made the discussion more structured following the reviewer suggestion of headings Summary of key findings → Comparison with previous evidence → Strengths/limitations → Policy/program implications → Broader/global relevance.

We have used this opportunity to improve the clarity of first para which basically begins with a line to market the study followed by summary of key findings (without repeating the numbers from results narrative).

We hope this is now fine.

COMMENT

First para of introduction – which other strategies? This was the comment made by reviewer against introduction (track changes) in the attachment shared

RESPONSE

In the revised first para of the introduction, we have clarified that these strategies are enhanced and active case finding.

COMMENT

Need to be tightened. At present it includes irrelevant details and definitions - comment made by reviewer against study settings (track changes) in the attachment shared

RESPONSE

We have now divided the setting into general and specific setting and tightened both these subsections. Specifically, the irrelevant sentences from general setting have been deleted.

COMMENT

Summary of key findings → Comparison with previous evidence → Strengths/limitations → Policy/program implications → Broader/global relevance—will improve readability -- comment made by reviewer against discussion section (track changes) in the attachment shared

RESPONSE

We have made the discussion more structured following the reviewer suggestion of headings Summary of key findings → Comparison with previous evidence → Strengths/limitations → Policy/program implications → Broader/global relevance

We have used this opportunity to improve the clarity of first para which basically begins with a line to market the study followed by summary of key findings (without repeating the numbers from results narrative).

We hope this is now fine.

********************

**REVIEWER 2**

COMMENT

Thank you for all the revisions. All my previous comments have been addressed well. I only have a small suggestion regarding the first sentence of the abstract.

Line no: 50-51-Since 2017, tuberculosis active case-finding (TB ACF) in India is being implemented in routine national TB elimination program settings. You may consider revising this sentence to make it easier to read- Since 2017, tuberculosis active case-finding (TB ACF) has been implemented within the routine framework of India’s national TB elimination program.RESPONSE

RESPONSE

Thank you. The suggested change has been incorporated into the first line of abstract.
